# Small-molecule ionic liquid-based adhesive with strong room-temperature adhesion promoted by electrostatic interaction

**DOI:** 10.1038/s41467-022-32997-4

**Published:** 2022-09-05

**Authors:** Jun Zhang, Wenxiang Wang, Yan Zhang, Qiang Wei, Fei Han, Shengyi Dong, Dongqing Liu, Shiguo Zhang

**Affiliations:** 1 College of Materials Science and Engineering, Hunan University, Changsha, 410082 China; 2 College of Chemistry and Chemical Engineering, Hunan University, Changsha, 410082 China; 3 Science and Technology on Advanced Ceramic Fibers and Composites Laboratory, College of Aerospace Science and Engineering, National University of Defense Technology, Changsha, 410073 China

**Keywords:** Organic molecules in materials science, Polymers, Organic molecules in materials science

## Abstract

Low-molecular-weight adhesives (LMWAs) possess many unique features compared to polymer adhesives. However, fabricating LMWAs with adhesion strengths higher than those of polymeric materials is a significant challenge, mainly because of the relatively weak and unbalanced cohesion and interfacial adhesion. Herein, an ionic liquid (IL)-based adhesive with high adhesion strength is demonstrated by introducing an IL moiety into a Y-shaped molecule replete with hydrogen bonding (H-bonding) interactions. The IL moieties not only destroyed the rigid and ordered H-bonding networks, releasing more free groups to form hydrogen bonds (H-bonds) at the substrate/adhesive interface, but also provided electrostatic interactions that improved the cohesion energy. The synthesized IL-based adhesive, Tri-HT, could directly form thin coatings on various substrates, with high adhesion strengths of up to 12.20 MPa. Advanced adhesives with electrical conductivity, self-healing behavior, and electrically-controlled adhesion could also be fabricated by combining Tri-HT with carbon nanotubes.

## Abstract (web summary)

Low-molecular-weight adhesives (LMWAs) possess many unique features compared to polymer adhesives but fabrication of LMWAs with adhesion strengths higher exceeding those of polymeric materials is a significant challenge. Here, the authors propose an ionic liquid based thin coating adhesive with high adhesion strength.

## Introduction

Adhesives play a prominent role in various industries, including automotive, electronics, medical, construction, labeling, and packaging. Many novel adhesives, including catechol-based materials, have been developed over the past few decades^1^. Generally, polymeric structures with intra-chain covalent bonds and inter-chain entanglement networks are essential for achieving a high adhesion strength^2^. However, polymeric materials usually exhibit high viscosities at high temperatures, which reduces their ability to penetrate target substrates, especially porous substrates. The high viscosities also lead to thick adhesion coatings, which not only causes adhesive waste but also limits their application in flexible devices. Even though organic solvents can be used to dissolve polymeric materials and reduce the layer thickness, this leads to volatile organic compound emissions, unwanted bubbles, and even adhesion failure. The repeated use of polymer adhesives also causes fatigue of molecular components^3–6^.

In contrast to polymer adhesives, low-molecular-weight adhesives (LMWAs) have many advantages, such as the ease of synthesis and processing, in-situ formation of adhesive layers, adaptive adhesion, and sufficient molecule–interface interactions, which may address the above-mentioned issues^7^. However, to date, only a limited number of LMWAs has been reported, and their adhesion strengths are usually in the range of several megapascals, with some of them even in the order of kilopascals (Supplementary Table 1). The low adhesion strength is ascribed to a lack of intermolecular covalent interactions in the LMWAs. A common approach to overcome this obstacle is to introduce abundant non-covalent interactions into LMWAs to obtain supramolecular adhesives with enhanced cohesive and interfacial adhesive energy. A majority of studies are based on macrocycle-involved host−guest interactions, van der Waals forces, π − π interactions, and particularly, H-bonding^7–11^. For example, 2-amino-4-hydroxy-6-methylpyrimidine motif-based LMWAs exhibit strong adhesion via multivalent H-bonding interactions^12^. However, the very high-density and strong H-bonding interactions are liable to form ordered and rigid structures because of the directional attraction with saturation, particularly for low-molecular-weight compounds, such as polysaccharides and urea derivatives^13,14^. It may also give rise to less non-complexed H-bonding donors (HBD)/acceptors (HBA) available for interfacial adhesion, resulting in brittle materials at room temperature without significant affinity for the target surfaces. Therefore, the balance between the degree of cohesion and interfacial adhesion should be carefully controlled to achieve an optimal interplay for strong adhesion.

Ionic bonds formed through electrostatic interactions between oppositely charged ions are much stronger than H-bonds. Recently, a few studies have proved that electrostatic interactions in mussel adhesives, polyampholyte hydrogels, and ionoelastomers contribute to enhanced adhesion^15–20^. For example, an alkyl ammonium group can interact with the catechol moieties to synergistically promote adhesion on the mica surface, while catechols alone are insufficient to breach the hydrated salt layer on mica^17^. Nevertheless, the effects of electrostatic interactions on adhesion are still not clearly understood and underutilized. Ionic liquids (ILs) with a strong intrinsic electric field and high cohesive energy are interesting examples involving ionic bonds^21,22^. ILs are salts of organic cations and inorganic or organic anions and have attracted significant attention owing to their non-flammability, low vapor pressure, and high chemical, thermal, and electrochemical stability as well as structural diversity^23^. Considering the non-directional and unsaturated nature of electrostatic interactions as well as the noteworthy properties of ILs, it can be envisioned that the presence of bulky and asymmetric IL structures may destroy the rigid and highly ordered networks in hydrogen-bonding-rich molecules, leaving more free HBD/HBA moieties at the surface. The high binding energy resulting from electrostatic interactions is also expected to contribute to the cohesion energy.

To verify this hypothesis, a Y-shaped IL (Tri-HT) containing an ionic moiety, urea, and dihydroxy groups in the tri-branched units was designed. The coexistence of electrostatic interactions and multiple dynamic H-bonding interactions endowed Tri-HT with significant temperature-sensitive viscoelasticity, which was beneficial for adhesion. Tri-HT could be used as a hot-melt LMWA without the need for any external solvent, and it demonstrated a high degree of adhesion on various substrates with a maximum adhesion strength of up to 12.20 MPa, which is among the highest values for LMWAs reported thus far. Owing to the high compatibility between ILs and multi-walled carbon nanotubes (MWCNTs), multifunctional composite adhesives with electrical conductivity, self-healing behavior, and electrically-controlled adhesion were also fabricated.

## Results

### Synthesis and properties

**Fig. 1:**
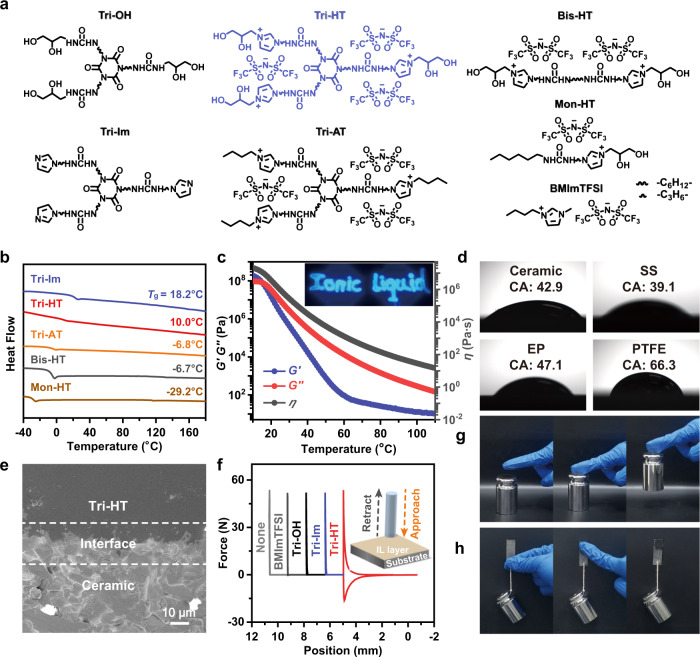
Structures and properties of the Tri-HT and reference compounds. **a** Chemical structures and **b** DSC curves. **c** Temperature-dependent rheological test of Tri-HT (angular frequency = 10 rad s^−1^, strain = 1%). Inset shows the room-temperature photograph of Tri-HT on PTFE under ultraviolet light irradiation after being extruded from a syringe at a high temperature. **d** Contact angles of molten Tri-HT on various substrates at 100 °C. **e** SEM image of the interface between Tri-HT and the ceramic substrate. (**f**) Probe-tack test of Tri-HT and reference compounds at 25 °C. **g**, **h** Room-temperature macroscopic adhesion (500 g weight) of Tri-HT on rubber gloves or plastic sheets by finger pressing.

Tri-HT and reference compounds (Fig. 1a) were synthesized (see Supplementary Information), and their chemical structures were confirmed by nuclear magnetic resonance (NMR), and Fourier transform infrared (FT-IR) spectroscopy (Supplementary Figs. 1–16). Thermogravimetric analysis (TGA) showed that Tri-HT had no appreciable weight loss until 300 °C, implying higher thermal stability than non-ionic derivatives (Tri-OH and Tri-Im) and non-branched ILs (Bis-HT and Mon-HT) (Supplementary Figs. 17 and 18). Similar to conventional ILs, Tri-HT was non-flammable even when exposed to a flame for 20 s, while the reference compound, Tri-OH, could be ignited in less than 3 s (Supplementary Fig. 19 and Supplementary Movie 1). Significantly, non-ionic Tri-OH was obtained in brittle powder form at room temperature, and X-ray diffraction (XRD) measurements showed several sharp crystallization peaks (Supplementary Fig. 20), suggesting its crystalline structure. Considering the presence of abundant urea and dihydroxy groups in addition to alkyl chains in the branched units, the crystallization of Tri-OH molecules is closely related to the multiple and strong hydrogen bonding interactions, rather than the weak van der Waals forces^24^. Such strong interactions were indeed verified by the gelation phenomenon in organic solvents, such as dimethyl formamide (DMF) (Supplementary Fig. 21)^25^. However, Tri-HT was amorphous, as its XRD pattern showed only broad peaks (Supplementary Fig. 20). Tri-HT can be liquefied and flowable at high temperature (e.g., 100 °C) but could be restored to a gel-like solid upon cooling to room temperature (Supplementary Fig. 22). This difference was also confirmed by a variety of other techniques, including differential scanning calorimetry (DSC), scanning electron microscopy (SEM), light microscopy (LM), and polarizing microscopy (Fig. 1b and Supplementary Fig. 23). Tri-HT exhibited a low glass transition temperature (*T*_g_) of 10.0 °C, while Tri-OH possessed a very high melting point (*T*_m_) of 170.2 °C (Fig. 1b and Supplementary Fig. 24).

Tri-HT exhibited clear temperature-sensitive rheological behavior. As shown in Fig. 1c, as the temperature increased from 10 to 100 °C, the storage moduli (*G´*), loss moduli (*G´´*), and complex viscosity (*η*) substantially decreased by 6–7 orders of magnitude. Such temperature-dependent viscoelasticity behavior has rarely been reported for traditional polymer adhesives and is significant even when compared with other reported supramolecular network polymers or LMWAs (Supplementary Table 2)^9^. The very high *G*´, *G*´´, and *η* at low temperatures reflected intensive intermolecular interaction for strong adhesion under ambient conditions, while the low η at high temperatures made Tri-HT flowable and deformable for solvent-free processability. As shown in the inset of Fig. 1c, molten Tri-HT could be easily extruded from a syringe into shapes onto a polytetrafluoroethylene (PTFE) substrate. The contact angle test showed that molten Tri-HT could wet various substrates, including ceramic, stainless steel (SS), epoxy (EP), and even PTFE with a low surface energy (Fig. 1d). The low viscosity and efficient wetting behavior of Tri-HT enabled it to penetrate the substrates and ensured an intimate contact between the adhesive and surfaces and minimized the defects at the interface regions (Fig. 1e).

### Adhesion performance

Tri-HT could be directly used as a pressure-sensitive adhesive (PSA) for room-temperature adhesion. As shown in Fig. 1f and Supplementary Fig. 25, no retraction force was observed in the force-position curves from the probe-tack test for non-ionic derivatives (Tri-OH and Tri-Im) and conventional IL (BMImTFSI) while Tri-HT exhibited an obvious adhesive feature at room temperature, and the observed force was repeatable and sensitive to the preloading force. The 180° peeling test also confirmed that Tri-HT possessed a high peel strength comparable to other PSAs (Supplementary Fig. 26)^26^. Macroscopic adhesion tests at room temperature showed that Tri-HT with an adhesion area of 1 cm^2^ could support a weight of 500 g by simple finger pressing (Fig. 1g, h). Damaged balloons could be repaired using Tri-HT and subsequently blown up without any leakage. The Tri-HT-adhered broken glass or plastic bottles could sustain water blasting with a hydraulic pressure of ~0.20 MPa (Supplementary Fig. 27, a–c and Supplementary Movie 2). Such processes could also be readily carried out underwater because of the hydrophobicity of Tri-HT (Supplementary Fig. 27d and Supplementary Movie 3).

**Fig. 2:**
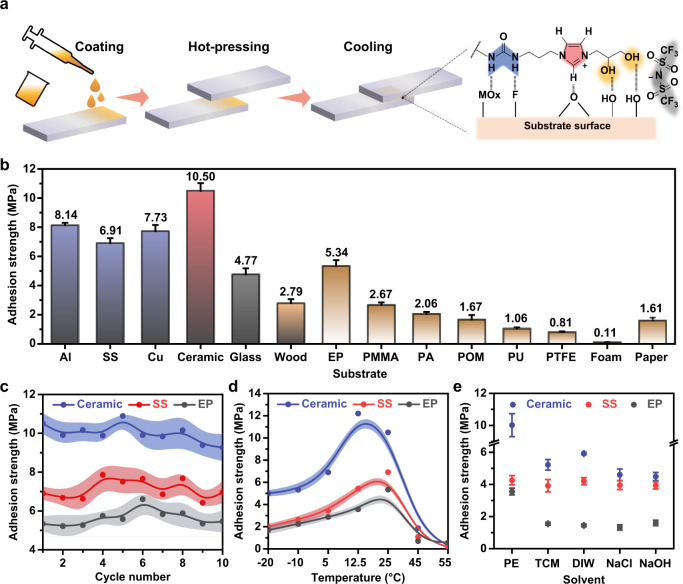
Measurements of adhesion strength. **a** Schematic of the adhesion procedure. **b** Adhesion strengths of Tri-HT on various substrates under ambient conditions. **c** Successive cycling tests and **d** temperature-dependent adhesion strengths of Tri-HT on various substrates. **e** Adhesion strengths of Tri-HT after soaking in various solutions for 24 h. For **b**, **c** and **e**, tests were carried out at 25 °C. Error bars correspond to the standard deviation of 3–5 measurements for each analysis.

Without any organic solvent or complicated re-solidification process, a very thin (~5 μm) adhesive layer (~1.7 mg cm^−2^) of Tri-HT could be obtained by depositing it on one substrate followed by hot pressing using another substrate (Fig. 2a and Supplementary Fig. 28). The thin Tri-HT layer exhibited a strong adhesion. For example, an adhesion area of 12 cm^2^ on ceramic obtained by hot melting pre-treatment could support a weight of 66 kg without detachment (Supplementary Fig. 29 and Supplementary Movie 4). Quantitative lap-shear tests revealed a substrate-dependent adhesive performance. The average adhesion strengths of Tri-HT on metal and metal oxide surfaces were much higher than those on other substrates, such as glass, wood, EP, polymethylmethacrylate (PMMA), polyamide (PA), polyformaldehyde (POM), polyurethane (PU), PTFE, foam, and paper (Fig. 2b). In particular, the adhesion strength on the ceramic substrate was as high as 10.50 MPa at room temperature. The observed adhesion performance was superior to that of commercial adhesives (for example, silicone, double-sided tape, polyurethane resin, and several hot-melt adhesives) on investigated substrates (Supplementary Fig. 30 and 31). In addition, Tri-HT showed a good reproducibility of adhesion in 10 adhesion–separation cycles (Fig. 2c). Because of its special rheological properties, the adhesion behavior of Tri-HT was temperature-sensitive. Upon heating from −20 to 55 °C, the adhesion strength on the ceramic substrate initially increased to 12.20 MPa at 12.5 °C, which was near its *T*_g_ of 10 °C, and then decreased to 0.30 MPa (Fig. 2d). Note that even at the respective sweet-spot temperature, the maximum adhesion strength of Tri-HT is obviously higher than that of reference compounds and most commercial adhesives, and even comparable with that of traditional polymer holt melt adhesives, such as poly(ethylene-co-vinyl acetate) (EVA), PA, polyolefins (PO) (Fig. 2b and Supplementary Fig. 32). Tri-HT also worked well under harsh conditions. No apparent attenuation of adhesion was observed for Tri-HT even when stored under ultraviolet radiation or vacuum for one month (Supplementary Fig. 33), which was advantageous compared to hydrogel adhesives and water-based supramolecular adhesives^27^. Tri-HT was also insoluble in many organic solvents, such as petroleum ether (PE), trichloromethane (TCM), deionized water (DIW), and aqueous solutions containing NaCl or NaOH. The adhesion strengths of Tri-HT on ceramic, SS, and EP substrates could retain at least 50% of their initial values after soaking in these solutions for 24 h (Fig. 2e). Even after soaking in DIW for 4 weeks, adhesion failure was not observed (Supplementary Fig. 34).

The adhesion performance was highly impacted by the chemical structure. Controlled experiments with a series of derivatives (Fig. 1a) showed that the adhesion strength distinctly decreased in the order Tri-HT > Tri-Im > Tri-AT on all substrates (Supplementary Fig. 35), suggesting the indispensable role of the ionic linker and terminal dihydroxy group in strong adhesion. It was impossible to test the adhesion strength of non-ionic Tri-OH because of its very high *T*_m_ and decomposition before melting (Supplementary Figs. 24 and 36). The highly branched structure was also important in determining the adhesion strength, as observed from the very low adhesion strength (0.11 MPa, only 1/10th of Tri-HT) of Bis-HT and the non-adhesive feature of Mon-HT (Supplementary Fig. 35). This was mainly because both samples possessed low *T*_g_ values (−29.2 and −6.7 °C) and were flowable (*G´´* > *G´*, Supplementary Figs. 37–40).

### Application to advanced composite adhesives

**Fig. 3:**
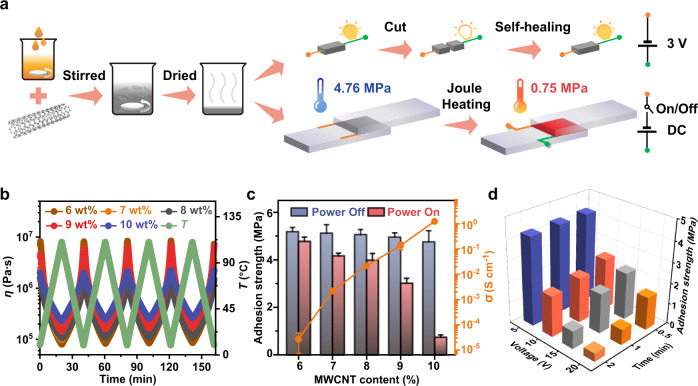
Application of Tri-HT adhesive materials. **a** Schematic of preparation of multifunctional Tri-HT/MWCNTs. During joule heating, copper foil tapes were attached to the edge of the substrates for electrical connection. The direction of current is distinguished by different colors. **b** Temperature-dependent viscosity of Tri-HT/MWCNTs. **c** Adhesion strengths and electronic conductivity of Tri-HT/MWCNTs on glass substrate as a function of the MWCNT content and triggered by Joule-heating effect (20 V, 1 min). **d** Adhesion strengths of Tri-HT/MWCNTs (10 wt%) as a function of voltage and time. All tests were carried out at 25 °C. Error bars correspond to the standard deviation of 3–5 measurements for each analysis.

Electrically conductive composite adhesives were fabricated by mixing Tri-HT and MWCNTs (Fig. 3a and Supplementary Figs. 41 and 42). SEM and LM images revealed the homogeneous distribution of MWCNTs in the adhesive matrix (Supplementary Figs. 43–45). With the increasing MWCNT content from 6 to 10 wt%, the resultant electrical conductivity could be enhanced from 2.72 × 10^−5^ to 1.24 S cm^−1^ (Fig. 3c). The temperature-sensitive viscoelastic features of Tri-HT were preserved in the composite adhesives, while a distinct gel-like behavior with *G´* > *G´´* was observed for Tri-HT/MWCNTs (Fig. 3b and Supplementary Figs. 46–50). This was possibly due to the good compatibility and special interactions between Tri-HT and MWCNTs, as suggested by the appearance of a new glass transition for all composites at 114–118 °C in addition to the *T*_g_ of Tri-HT (Supplementary Fig. 51)^28,29^. The tensile strength of Tri-HT/MWCNTs significantly increased from 0.75 to 2.00 MPa with the increasing MWCNT content from 7 to 10 wt%, while Tri-HT itself and composite adhesives containing 6 wt% MWCNTs exhibited a weak mechanical strength and could not be shaped for stress-strain testing (Supplementary Fig. 52a). The composite materials exhibited self-healing properties. Without any solvent or external force, the fractures of Tri-HT/MWCNTs (10 wt%) could be re-connected within 24 h, yielding a tensile strength of 0.96 MPa (Supplementary Fig. 52b). Even after exposing the fracture surface to air for a long time (for example, 150 s), electrical healing phenomena were observed at room temperature (Supplementary Fig. 53)^30^.

Furthermore, Tri-HT/MWCNT composites were explored as responsive adhesives by coupling the temperature-dependent adhesion of Tri-HT with the Joule-heating effect of MWCNTs (Supplementary Fig. 54)^31,32^. The local temperature of Tri-HT/MWCNTs (10 wt%) increased to 142 °C within 2 min on applying a voltage of 20 V and considerably dropped to room temperature when the power was turned off (Supplementary Fig. 55). The Joule-heating effect was enhanced by increasing the MWCNT content from 6 to 10 wt%, while the corresponding adhesion strength of Tri-HT/MWCNTs decreased from 5.19 to 4.76 MPa (Fig. 3c and Supplementary Figs. 55–58). Therefore, 10 wt% MWCNT was the optimal composition for testing. On applying a voltage of 20 V for 1 min, a significant decrease (84.2%) in the initial adhesion strength was observed for Tri-HT/MWCNTs (10 wt%), which was much higher than those of the other samples (Fig. 3c, d). The macroscopic adhesion test revealed that the glass substrates adhered by this composite adhesive could sustain a weight of 6 kg, while on-demand adhesion failure occurred after Joule-heating for 1 min (Supplementary Fig. 59 and Supplementary Movie 5). Significantly, the electrically-controlled adhesion was highly reversible and was applicable to other substrates as well (Supplementary Figs. 60 and 61).

### Proposed mechanism for adhesion

**Fig. 4:**
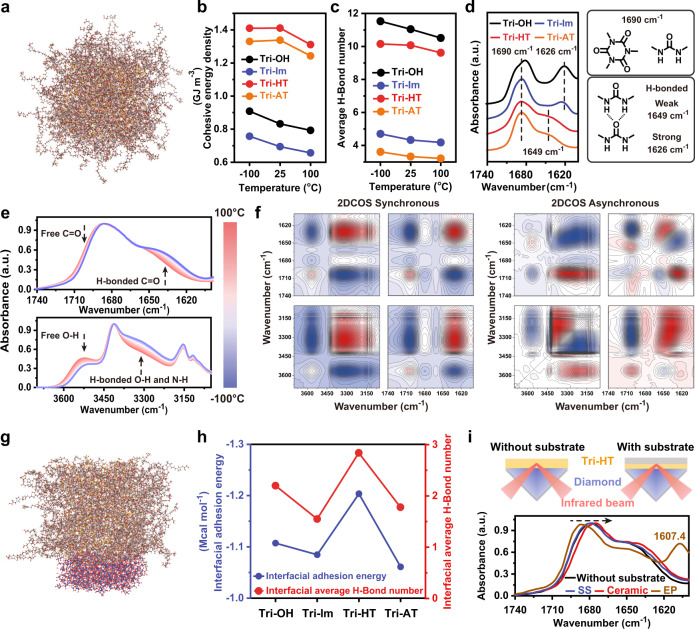
Internal and external interactions of Tri-HT. **a** Most stable conformation with a minimum energy of Tri-HT without the polyhydric substrate. **b** CED and **c** average H-bond number of Tri-HT and reference compounds at different temperatures. **d** FT-IR spectra showing the difference of C = O characteristic groups in Tri-HT and reference compounds. **e** Temperature-dependent FT-IR spectra of Tri-HT upon cooling from 100 to −100 °C (interval: 20 °C). **f** Two-dimensional COS synchronous and asynchronous spectra generated from **e**, wherein red and blue colors are defined as positive and negative intensity, respectively. **g** Most stable conformation with a minimum energy of Tri-HT on the polyhydric substrate surface. **h** IAE and interfacial average H-bond number of Tri-HT and reference compounds. **i** ATR-IR spectra of Tri-HT with or without substrate (ceramic, SS, and EP).

Based on the established adhesion theory, the strong adhesion of Tri-HT was attributed to the high cohesive energy density (CED) and interfacial adhesion energy (IAE)^33,34^. Molecular dynamic (MD) simulation revealed that the CED of ionic Tri-HT (1.41 GJ m^−3^) and Tri-AT (1.34 GJ m^−3^) at room temperature were much higher than those of non-ionic Tri-OH (0.83 GJ m^−3^) and Tri-Im (0.69 GJ m^−3^) (Fig. 4a–b and Supplementary Table 3). Cohesive energy of Tri-HT and reference compounds mainly results from van der Waals forces, H-bonding, and electrostatic interactions, wherein the contributions of van der Waals forces were comparable for all these samples (Supplementary Fig. 62). In contrast, the average number of H-bonds in the ILs (Tri-HT and Tri-AT) was always lower than those of the non-ionic analogs (Tri-OH and Tri-Im) at all temperatures (Fig. 4c, Supplementary Figs. 63–67 and Supplementary Table 4). A closer inspection of the FT-IR spectra revealed that in addition to free C = O in the hexagonal core (1690 cm^−1^), the IR band of the C = O in the branched units shifted to 1626 cm^−1^ in Tri-OH and Tri-Im, demonstrating the presence of strong H-bonds (Fig. 4d)^35^. When imidazolium cations and bis(trifluoromethanesulfone)imide anions were incorporated, the corresponding IR band shifted to 1649 cm^−1^ in Tri-HT and Tri-AT, indicating the presence of weak H-bonds^35^. Therefore, the introduction of the IL moiety not only enhanced the CED significantly via improved electrostatic interactions but also partially inhibited the formation of strong and highly packed H-bonds. The suppressed H-bonding interactions were also reflected by X-ray photoelectron spectroscopy (XPS) analysis. As shown in Supplementary Fig. 68, the high-resolution N 1 s spectrum of Tri-HT could be fitted to H-bonded N, free N-H groups, imidazolium N, hexatomic ring N, and anionic N^36–39^. The areal percentage of H-bonded N in the first two components was 21.5% for Tri-HT, which was much lower than the 63.8% for Tri-OH.

To gain insight into the detailed H-bonding interactions in Tri-HT, variable-temperature FT-IR spectroscopy was conducted. Upon cooling from 100 to −100 °C, more H-bonded species were formed, as reflected by the attenuated intensity of the peaks at 1702 cm^−1^ (free C = O) and 3567 cm^−1^ (free O–H) along with the increased intensity of 1629 cm^−1^ (H-bonded C = O) and 3320 cm^−1^ (H-bonded O–H and N-H) peaks (Fig. 4e). As a powerful tool for extracting more subtle spectral information, two-dimensional correlation spectroscopy (2DCOS) analysis was carried out to discern the sequential thermal response of different species^40^. As shown in Fig. 4f, the auto peaks in the synchronous 2DCOS spectra at (1629, 1629 cm^−1^) and (3320, 3320 cm^−1^) are more distinct than those in the (1702, 1702 cm^−1^) and (3567, 3567 cm^−1^) region, suggesting that H-bonded groups exhibit much weaker motility and higher sensitivity to temperature than free groups when the temperature decreases^41^. The response order of the peaks in the asynchronous 2DCOS was as follows: 1702 cm^−1^ → (3567, 1675 cm^−1^)→(3567, 1629 cm^−1^)→3320 cm^−1^ → (3153, 3116 cm^−1^) (Fig. 4f, → means earlier than, and the brackets means that changes occurred simultaneously) (Supplementary Table 5)^42^, where peaks at 1675 and 1629 cm^−1^ could be ascribed to the C = O moiety with weak and strong H-bonding, respectively, while 3153 and 3116 cm^−1^ peaks could be attributed to (Im) C4/5-H and C2-H, respectively. According to Noda’s judging rule^43^, this result suggested that with the decreasing temperature, the free C = O moieties moved first and formed H-bonds with O–H groups, followed by N-H and then Im-C-H groups. Urea-based H-bonding interactions could be classified into weak and strong types; the former was observed in the 2DCOS spectra, while the latter was not detected but was likely stable even above 100 °C^44,45^. Further analysis of the perturbation correlation moving window (PCMW) spectra revealed that a significant change in these dynamic H-bonds occurred near room temperature (close to *T*_g_) (Supplementary Fig. 69), indicating that under ambient conditions, abundant H-bonding networks were formed, which resulted in the high modulus and viscosity observed in the viscoelastic properties of Tri-HT (Fig. 1c).

IAE was also calculated by MD simulation using a polyhydric surface ((HO)_3_SiOSi(OH)_4_OSi(OH)_3_) as a model substrate (Fig. 4g and Supplementary Figs. 70–73). The change of IAE (Tri-HT > Tri-AT > Tri-OH > Tri-Im) was similar to the interfacial average H-bond number (Fig. 4h and Supplementary Table 6), and a maximum IAE (−1.20 Mcal mol^−1^) was observed for Tri-HT, suggesting the dominant role of H-bonds in facilitating interfacial interaction with the substrate surface. Attenuated total reflectance infrared (ATR-IR) spectroscopy analysis in the range of 1600–1740 cm^−1^, in which the investigated substrates were IR transparent, were collected to investigate the interfacial H-bonds involving the urea C = O groups. Considering the limited penetration depth of the IR beam, the thickness of the Tri-HT coating was carefully controlled so that the characteristic absorption of both Tri-HT and the substrate could be detected. In such case, we could distinguish the interface region from the matrix. As seen from Fig. 4i and Supplementary Fig. 74, the stretching vibration of C = O evidently red-shifted in the order of ceramic > Tri-HT itself ≈ SS > EP, suggesting that the strongest interface H-bonds are between ceramic and Tri-HT, which also accounted for the high IAE and adhesion strength (Fig. 2b)^46^. The presence of an external substrate could strengthen the bulk H-bonding interactions in ILs, as revealed by the increased average H-bond numbers along with shortened bond lengths for Tri-HT and Tri-AT (Supplementary Figs. 75 and 76). In contrast, the non-ionic analogs (Tri-OH and Tri-Im) exhibited opposite changes with the polyhydric substrate. This result further demonstrated the potential of the IL moiety for facilitating an improved adhesion. Based on the above discussion, we can conclude that the presence of IL moieties resulted in loosely packed H-bonds not only in the bulk phase but also at the interface, leading to a large number of free HBA/HBD moieties that could form H-bonds at the substrate/adhesive interface, as depicted in Fig. 2a. This conclusion was confirmed by the experimentally observed room-temperature adhesion of Tri-HT without any heat treatment and was similar to that of the recent study on self-healing glassy polymers^47^. In contrast, very high-density and strong H-bonding interactions are likely to form ordered and rigid structures, yielding brittle materials. The lack of significant affinity for the target substrates at room temperature and stress concentration at the edge of the adhesive layer could deteriorate the adhesion effect^48–50^.

## Discussion

In summary, this study provided a strategy and mechanism to construct small-molecule IL-based adhesives. The low-molecular weight presents Tri-HT with highly temperature-sensitive viscoelastic properties, which were beneficial for achieving a thin coating for the efficient penetration and wetting of the substrate at high temperatures and strong adhesion at room temperature. Experimental analysis and theoretical calculations revealed that the IL moiety played multiple roles in strong adhesion. It improved the cohesive energy via strong electrostatic interactions and prevented the formation of ordered packing structures originating from high-density, multiple H-bonds, resulting in numerous free complementary moieties of H-bonding for interfacial adhesion. The simultaneously high cohesive and interfacial adhesive energy endowed Tri-HT with high-performance adhesion compared to the reference compounds and commercial adhesives. Consequently, Tri-HT as a hot-melt adhesive, exhibited strong adhesion to various substrates, including metal, glass, wood, ceramic, and polymers, with a maximum adhesion strength of up to 12.20 MPa. When combined with CNTs, electrically conductive adhesives with self-healing properties could be obtained, and the temperature-dependent adhesion was used to design composite adhesives with on-demand adhesion via the Joule-heating effect. Significantly, this study highlighted the conversion of the disadvantageous high viscosity of ILs for solvent/electrolyte applications to an advantageous point for adhesion. The IL moiety-incorporated strategy paves the way for the realization of advanced LMWAs through ionic bonds, and the physico-chemical properties can be rationally tuned by metathesized anions or functional groups in the future.

## Methods

### Synthesis of ionic liquid-based adhesives

The detail of the synthesis of ionic liquid-based adhesives can be found in Supplementary Methods.

### Chemicals, materials, and characterizations

Tri-NCO was purchased from Bayer. 3-Amino-1,2-propanediol, N-(3-aminopropyl)-imidazole, 3-chloro-1,2-propanediol, lithium bis(trifluoromethanesulfonyl)imide (LiTFSI, 99.95%), 1-bromobutane, hexamethylene diisocyanate (Bis-NCO), and hexyl isocyanate (Mon-NCO) were purchased from Aldin, China. BMImTFSI was purchased from Lanzhou Greenchem ILs (China). All reagents were commercially available and used without further purification. Multiwall carbon nanotubes (MWCNTs) powder (4–6 nm outer diameter and 10–20 µm length) was purchased from Nanjing XFNANO Co., Ltd (China). Ultra-pure purification system (Master-S15Q, Hitech Instruments Co. Ltd., Shanghai, China) was used to produce 18.2 MΩ cm^−1^ of water in all experiments.

NMR spectra were collected on a Bruker 400 MHz with TMS as the internal standard. High-resolution mass spectra were collected on a Shimadzu LCMS-IT-TOF under the specific conditions (ESI + spray voltage, 4.5 kV, or ESI − spray voltage, −3.5 kV; nebulizer gas, 1.5 L min^−1^; drying gas, 100 kPa; heat block temperature, 200 °C; CDL temperature, 200 °C; IT Area Vacuum, 1.0 × 10^−2^ Pa; TOF Area Vacuum, 5 × 10^−4^ Pa). Ion accumulation time was set to 10 ms, and the detector voltage was fixed at 1.6 kV. Fourier transform infrared (FT-IR) and attenuated total reflectance infrared (ATR-IR) spectra were obtained on a Thermo Fisher Scientific Nicolet iS50 spectrometer equipped with a diamond single reflection attenuated total reflectance (ATR) accessory. Thermogravimetric (TG) analysis was obtained on a Hitachi STA7200 Thermal Analysis System at a heating rate of 10 °C min^−1^ under an inert atmosphere of argon. X-ray diffraction (XRD) spectra were obtained on an X-ray diffractometer (Rigaku, MiniFlex600). Differential scanning calorimetry (DSC) was performed using a DSC822e Mettler-Toledo at a 10 °C min^−1^ heating/cooling rate under an inert atmosphere of nitrogen (all values of *T*_g_ were collected during the second heating cycle). X-ray photoelectron spectroscopy (XPS) was obtained on an ESCALAB 250XI photoelectron spectrometer. Morphologies of Tri-OH, Tri-HT, and Tri-HT/MWCNTs composite were characterized using a Hitachi-S4800 scanning electron microscope (SEM). All optical photographs (including reflection and transmission modes) were collected on an OptoNano 200 (Optosigma Corporation). Rheological measurements were processed on an Anton Paar MCR 92. The laminator was chosen with a diameter of 25 mm, cone angle of 1°, and a gap of 0.051 mm. The contact angles (CA) of molten Tri-HT on various substrates were obtained using an SDC-200S optical contact angle measuring instrument at 100 °C. UV-Vis transmittance spectra were collected on a Shimadzu UV-2600 spectrophotometer. Raman spectra of MWCNTs and Tri-HT/MWCNTs composites were collected using DXR Raman Microscope with a 532 nm laser source. Adhesion strengths were measured on an HY-0580 tension machine at a constant speed of 100 mm min^−1^. Unless particularly noted, each test was carried out 5 times.

### Adhesion strength tests

Molten Tri-HT was coated on the surface of the substrate using a scraper, and another substrate was covered. After pressing by a weight of 3 kg for 30 min at 100 °C, a thin adhesive layer (10 mm × 10 mm) about a thickness of 5 μm was formed, and two substrates adhered firmly.

### The cycling adhesion strength test of Tri-HT on various substrates

After the lap-shear test, separated substrates were heated (100 °C) and adhered them again. The adhesion area was pressed again at 100°C. Each test was carried out at least 3 times.

### Preparation of Tri-HT/MWCNTs composite with electrically conductive, self-healing, and controlled adhesive properties

To obtain a homogeneous mixture of Tri-HT and MWCNTs, the solvent of DMF was used. A certain amount of Tri-HT and MWCNTs was dissolved or dispersed in DMF, respectively. Then, the two solutions were mixed and stirred for 12 h. After being dried at 100 °C for 15 h, the composite was obtained. The electronic conductivity measurements of the Tri-HT/MWCNTs composite were measured with a standard four-probe method on an RTS-8 four-probe instrument. All stress-strain curves of the Tri-HT/MWCNTs composites were measured at room temperature at a constant speed of 100 mm min^−1^. Samples were cut in a shape of 30 mm × 5 mm × 10 mm. The self-healing experiments were performed by bringing damaged samples together and storing them at 100 °C for 12 h. The current-voltage curves and current-time curves were measured using a CHI 660 electrochemical workstation. The procedures of the adhesion strength test were similar to the before-mentioned, except for increasing the temperature from 100 to 110 °C and the accessional copper foil tapes (for electrical connection) on the edge of the substrates. During the test, the adhesion area could be electrically circulated by copper tapes. The circulated time ranged from 0.5 to 2 min. Meanwhile, the temperature of the sample in the joule-heating process was recorded by an IR thermal imager (FLUKE TI450).

### Temperature-dependent FT-IR measurement

Molten Tri-HT was cast on a KBr tablet and heated at 100°C in a vacuum and sealed chamber with CaF_2_ window for half an hour before the test. An ultra-thin film of Tri-HT on a KBr tablet was obtained for transmission IR measurement. The spectra range was from 4000 to 400 cm^−1^ with 32 scans at a resolution of 4 cm^−1^. For temperature-controlled measurement, the samples were cooled from 100 to −100 °C with an interval of 20 °C, and each temperature holds for 10 min to reach equilibrium.

### Two-dimensional correlation spectroscopy (2DCOS)

The temperature-dependent FT-IR spectra of Tri-HT from 100 to −100 °C were used for analysis. 2DCOS and perturbation correlation moving-window (PCMW) spectroscopy were carried out using the software 2D Shige ver. 1.3 (Shigeaki Morita, Kwansei-Gakuin University, Japan, 2004–2005). In the contour maps, red colors are defined as positive intensities, and blue colors are defined as negatives. The responsive order of different groups can be judged by Noda’s rule. The PCMW spectroscopy was applied to monitor the transition temperature. The temperature with the strongest intensity in the synchronous and asynchronous spectra can be utilized to determine the transition temperature.

### Molecular dynamics simulation

In order to investigate the cohesive energy density of adhesive molecules and the interfacial interactions with polyhydric substrate surface, corresponding energies were calculated by Molecular Dynamics Simulation. In this work, molecular models of ILs (Tri-HT and Tri-AT), nonionic analogs (Tri-OH and Tri-Im) and substrate were respectively built using Materials Studio software as shown in Fig. 4a and g. Each model of ILs and nonionic analogs contain 50 and 80 molecules, respectively. As for constructing the interface adsorption models of adhesive molecules and polyhydric substrate surface, additional 80 repeated monomers (HO)_3_SiOSi(OH)_4_OSi(OH)_3_ were added to the above models, respectively. The Condensed-Phase Optimized Potentials for Atomistic Simulation Studies (COMPASS) force field was used. The COMPASS force field is commonly used to provide the atomic interactions. Then, the Ewald method and the atom-based method were employed for analyzing the Van der Waals interactions and electrostatic interactions between adhesive molecules and polyhydric substrate surface. In order to obtain a reasonable interaction configuration, a geometry optimization using smart method with an energy convergence criterion of 2.0 × 10^−5^ kcal mol^−1^ and force convergence criteria of 10^−3^ kcal mol^−1^ Å^−1^ were used to get a global minimum energy configuration. To equilibrate the model, an equilibrating process was followed under constant temperature (−100, 25, or 100 °C) and constant volume (NVT ensemble) for 10 ns. During the simulation, the Nosé-Hoover thermostat was applied to the temperature control. For the most stable conformation with minimum energy models, the restrictive geometry parameters of forming H-bonding interactions were a maximum hydrogen-acceptor distance of 2.5 Å and a minimum donor-hydrogen-acceptor angle of 90°. The cohesive energy density of adhesive molecules and interfacial adhesion energy between adhesive molecules and polyhydric substrate surface were calculated by the following formula.

***CED*** = ***E***_coh_/***V***

***E***_coh_ = −(***E***_inter_) = (***E***_intra_) − (***E***_total_), the brackets represent the time average.

***E***_coh_: The cohesive energy.

***V***: The volume of a system.

***E***_inter_: The total energy between all molecules.

***E***_intra_: The intramolecular energy.

***E***_total_: The total energy of a system.

***E***_interfacial_ (or ***IAE***) = ***E***_total_ −  (***E***_molecules_ + ***E***_substrate_)

***E***_interfacial_: Interaction energy between adhesive molecules and polyhydric substrate surface, minus means adsorption.

***E***_total_: The total potential energy of system.

***E***_molecules_: The potential energy of adhesive molecules.

***E***_substrate_: The potential energy of polyhydric substrate.

## Supplementary information

Supplementary Information

Description of Additional Supplementary Files

Supplementary Movie 1

Supplementary Movie 2

Supplementary Movie 3

Supplementary Movie 4

Supplementary Movie 5

## Data Availability

The data that support the plots within this paper and other finding of this study are available from the corresponding author upon request.
