# Supplementary material for: Small-molecule ionic liquid-based adhesive with strong room-temperature adhesion promoted by electrostatic interaction
Source: Nat Commun. 2022 Sep 5;13:5214. doi: 10.1038/s41467-022-32997-4 (PMC9445047; doi:10.1038/s41467-022-32997-4)
Supplement: Supplementary file 1 — Supplementary Information [file 41467_2022_32997_MOESM1_ESM.pdf]

Supplementary Information

Small-molecule ionic liquid-based adhesive with strong room-temperature adhesion promoted by electrostatic interaction

Jun Zhang[1], Wenxiang Wang[1], Yan Zhang[1], Qiang Wei[1], Fei Han[1], Shengyi Dong[2], Dongqing Liu[3], and Shiguo Zhang[1]*

[1]College of Materials Science and Engineering, Hunan University, Changsha 410004, China

[2]College of Chemistry and Chemical Engineering, Hunan University, Changsha 410082, China

[3]Science and Technology on Advanced Ceramic Fibers and Composites Laboratory, College of Aerospace Science and Engineering, National University of Defense Technology, Changsha 410073, China.

*Corresponding author. Email: zhangsg@hnu.edu.cn

Supplementary Methods

Synthesis of Tri-HT and other derivatives

Preparation of nonionic Tri-OH:

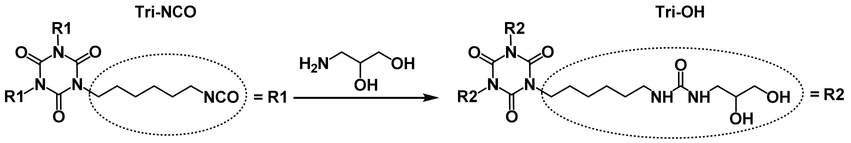

Tri-NCO (2.02 g, 4 mmol) was added into a solution of 20 ml tetrahydrofuran (THF) and 3-amino-1,2-propanediol (1.20 g, 13.2 mmol). After being stirred at room temperature for 24 h, white powders were precipitated from the solution. The product was washed with THF three times and dried under vacuum to obtain the Tri-OH (yield: 94%). ^{1}H NMR (400 MHz, DMSO-d_6) δ 6.00 (s, 1H), 5.81 (s, 1H), 4.77 (s, 1H), 4.54 (s, 1H), 3.71 (s, 2H), 3.43 – 3.12 (m, 6H), 2.93 (dd, J = 12.8, 6.9 Hz, 2H), 1.52 (s, 2H), 1.29 (d, J = 32.3 Hz, 7H). ^{13}C NMR (100 MHz, DMSO-d_6) δ 158.83, 148.93, 71.23, 63.58, 42.74, 42.22, 29.95, 27.26, 26.09, 25.98. HRMS (m/z): $[M]^+$ calcd. for $C_{33}H_{63}N_9O_{12}$, 777.4596; found, 777.4591.

Preparation of nonionic Tri-Im:

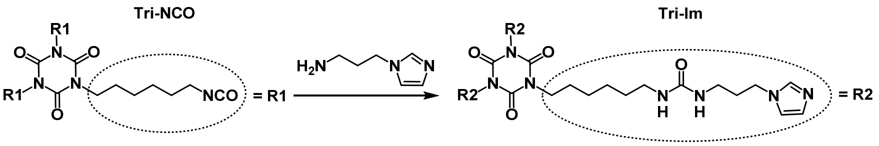

Tri-NCO (2.02 g, 4 mmol) was added into a solution of 20 ml THF and N-(3-aminopropyl)-imidazole (1.65 g, 13.2 mmol). After being stirred at room temperature for 24 h, white powders were precipitated from the solution. The product was washed with THF three times and dried under vacuum to obtain the Tri-Im (yield: 90%). ^{1}H NMR (400 MHz, DMSO-d_6) δ 7.60 (s, 1H), 7.15 (s, 1H), 6.87 (s, 1H), 5.83 (d, J = 29.8 Hz, 2H), 3.93 (s, 2H), 3.71 (s, 2H), 3.02 – 2.84 (m, 4H), 1.78 (s, 2H), 1.51 (s, 2H), 1.29 (d, J = 36.7 Hz, 6H). ^{13}C NMR (100 MHz, DMSO-d_6) δ 158.22, 148.89,

137.27, 128.35, 119.33, 43.68, 42.16, 36.49, 31.76, 29.94, 27.22, 26.06, 25.94. HRMS (m/z): $[M]^+$ calcd. for $C_{42}H_{69}N_{15}O_6$, 879.5555; found, 879.5550.

Preparation of Tri-HT:

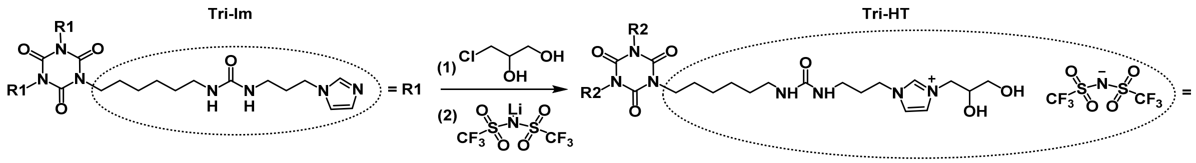

(1) Quaternization reaction: Tri-Im (4.40 g, 5 mmol) and 3-chloro-1,2-propanediol (4.97 g, 45 mmol) were combined in a 100 mL Schlenk flask. After being deoxygenated and backfilled with argon, the flask was stirred in an oil bath at 100 °C for 48 h. After cooling down, the mixture was added dropwise into an excess of ethyl acetate (EA). The precipitate was washed with EA three times and dried under vacuum to obtain the product with Cl anions (yield: 70%).

(2) Anion exchange reaction: 6.06 g of the product with Cl anions was dissolved in 100 mL of deionized water (DIW). An aqueous solution containing 6.46 g LiTFSI salt was added dropwise into the above solution. After being stirred at room temperature for 12 h, the precipitate was collected, dissolved in EA and extracted using DIW three times. The resulting solution was dried under vacuum to obtain the Tri-HT (yield: 92%). ^1H NMR (400 MHz, DMSO-d_6) δ 9.12 (s, 1H), 7.79 (s, 1H), 7.71 (s, 1H), 5.91 (d, J = 6.7 Hz, 2H), 5.34 (s, 1H), 4.94 (s, 1H), 4.30 (d, J = 16.6 Hz, 1H), 4.16 (t, J = 6.8 Hz, 2H), 4.06 (dd, J = 13.8, 8.0 Hz, 1H), 3.74 (d, J = 29.5 Hz, 4H), 3.42 (s, 1H), 3.26 (s, 1H), 2.97 (dq, J = 12.9, 6.2 Hz, 4H), 1.88 (p, J = 6.6 Hz, 2H), 1.51 (s, 2H), 1.25 (s, 6H). ^{13}C NMR (100 MHz, DMSO-d_6) δ 158.71, 149.11, 136.95, 123.50, 122.22, 118.14, 69.86, 62.91, 52.52, 46.98, 42.40, 36.18, 30.96, 30.03, 27.38, 26.24, 26.13. HRMS (m/z) ESI+: [M] calcd. for $C_{51}H_{90}N_{15}O_{12}$, 368.2292; found, 368.2271. ESI-: [M] calcd. for $C_2F_6NO_4S_2$, 279.9178; found, 279.9173.

Preparation of Tri-AT:

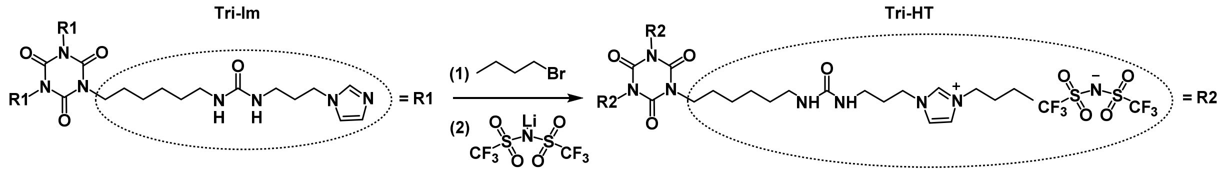

(1) Quaternization reaction: Tri-Im (4.40 g, 5 mmol), 1-bromobutane (2.47 g, 18 mmol) and 30 ml DMF were combined in a 100 mL Schlenk flask. After being deoxygenated and backfilled with argon, the flask was stirred in an oil bath at 60 °C for 24 h. After cooling down, the mixture was added dropwise into an excess of EA. The precipitate was washed with EA three times and dried under vacuum to obtain the product with Cl anions (yield: 80%).

(2) Anion exchange reaction: 6.46 g of the product with Cl anions was dissolved in 100 mL of DIW. An aqueous solution containing 6.46 g LiTFSI salt was added dropwise into the above solution. After being stirred at room temperature for 12 h, the precipitate was collected, dissolved in EA and extracted using DIW three times. The resulting solution was dried under vacuum to obtain the Tri-AT (yield: 95%). ^{1}H NMR (400 MHz, DMSO-d_6) δ 9.18 (s, 1H), 7.78 (d, J = 10.3 Hz, 2H), 5.90 (d, J = 18.2 Hz, 2H), 4.16 (t, J = 6.7 Hz, 4H), 3.72 (s, 2H), 2.99 (dd, J = 15.6, 6.2 Hz, 4H), 1.97 – 1.86 (m, 2H), 1.79 (p, J = 6.9 Hz, 2H), 1.53 (s, 2H), 1.31 (d, J = 37.8 Hz, 8H), 0.90 (t, J = 7.2 Hz, 3H). ^{13}C NMR (100 MHz, DMSO-d_6) δ 158.35, 148.91, 136.19, 122.55, 122.40, 117.96, 48.73, 46.90, 42.20, 36.04, 31.35, 30.74, 29.93, 27.24, 26.09, 25.97, 18.86, 13.20. HRMS (m/z) ESI+: [M] calcd. for $C_{54}H_{96}N_{15}O_6$, 350.2551; found, 350.2537. ESI-: [M] calcd. for $C_2F_6NO_4S_2$, 279.9178; found, 279.9173.

Preparation of Bis-HT:

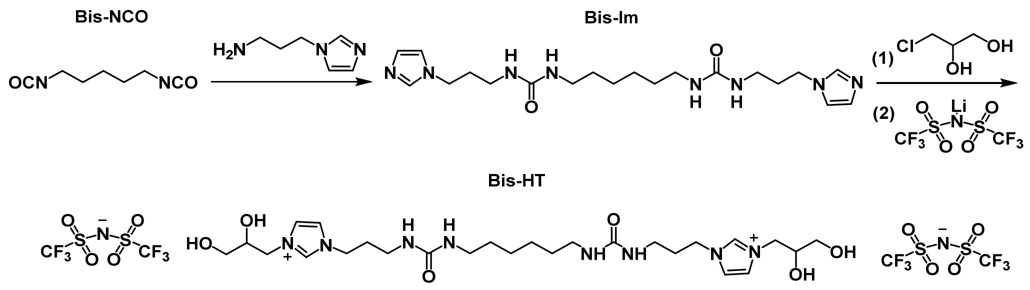

(1) Bis-NCO (0.77 g, 5 mmol) was added into a solution of 10 ml THF and N-(3-aminopropyl)-imidazole (1.38 g, 11 mmol). After being stirred at room temperature for 24 h, white powders were precipitated from the solution. The product was washed with THF three times and dried under vacuum to obtain the Bis-Im (yield: 85%).

(2) Quaternization reaction: Bis-Im (2.09 g, 5 mmol) and 3-chloro-1,2-propanediol (3.32 g, 30 mmol) were combined in a 100 mL Schlenk flask. After being deoxygenated and backfilled with argon, the flask was stirred in an oil bath at 100 °C for 48 h. After cooling down, the mixture was added dropwise into an excess of EA. The precipitate was washed with EA three times and dried under vacuum to obtain the product with Cl anions (yield: 75%).

(3) Anion exchange reaction: 3.20 g of the product with Cl anions was dissolved in 100 mL of DIW. An aqueous solution containing 4.31 g LiTFSI salt was added dropwise into the above solution. After being stirred at room temperature for 12 h, the precipitate was collected, dissolved in EA and extracted using DIW three times. The resulting solution was dried under vacuum to obtain the Bis-HT (yield: 90%). ^{1}H NMR (400 MHz, DMSO-d_6) δ 9.12 (s, 1H), 7.73 (d, J = 32.2 Hz, 2H), 5.88 (d, J = 17.5 Hz, 2H), 5.32 (s, 1H), 4.91 (s, 1H), 4.29 (dd, J = 13.8, 3.0 Hz, 1H), 4.16 (t, J = 6.9 Hz, 2H), 4.06 (dd, J = 13.8, 8.0 Hz, 1H), 3.78 (s, 1H), 3.45 – 3.22 (m, 4H), 2.97 (dt, J = 13.1, 6.4 Hz, 4H), 1.88 (p, J = 6.7 Hz, 2H), 1.29 (d, J = 46.5 Hz, 4H). ^{13}C NMR (100 MHz, DMSO-d_6) δ 136.93, 123.46, 122.17, 121.30, 69.83, 62.88, 52.48, 46.93, 36.13, 30.94, 30.13, 26.33.

HRMS (m/z) ESI+: [M] calcd. for $C_{26}H_{48}N_8O_6$, 284.1843; found, 284.1821. ESI-: [M] calcd. for $C_2F_6NO_4S_2$, 279.9178; found, 279.9173.

Preparation of Mon-HT:

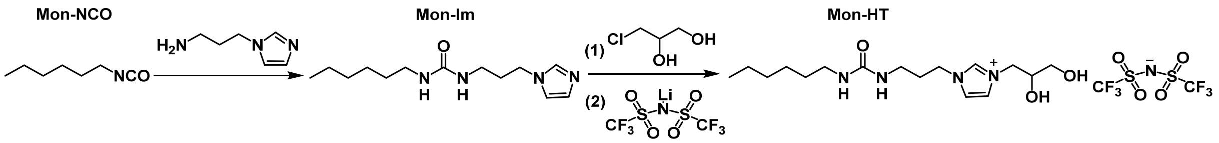

(1) Mon-NCO (0.64 g, 5 mmol) was added into a solution of 10 ml THF and N-(3-aminopropyl)-imidazole (0.69 g, 5.5 mmol). After being stirred at room temperature for 24 h, white powders were precipitated from the solution. The product was washed with THF three times and dried under vacuum to obtain the Mon-Im (yield: 80%).

(2) Quaternization reaction: Mon-Im (1.26 g, 5 mmol) and 3-chloro-1,2-propanediol (1.66 g, 15 mmol) were combined in a 100 mL Schlenk flask. After being deoxygenated and backfilled with argon, the flask was stirred in an oil bath at 100 °C for 48 h. After cooling down, the mixture was added dropwise into an excess of EA. The precipitate was washed with EA three times and dried under vacuum to obtain the product with Cl anions (yield: 70%).

(3) Anion exchange reaction: 1.81 g of the product with Cl anions was dissolved in 100 mL of DIW. An aqueous solution containing 2.16 g LiTFSI salt was added dropwise into the above solution. After being stirred at room temperature for 12 h, the precipitate was collected, dissolved in EA and extracted using DIW three times. The resulting solution was dried under vacuum to obtain the Mon-HT (yield: 80%). ^{1}H NMR (400 MHz, DMSO-d_6) δ 9.12 (s, 1H), 7.74 (d, J = 33.7 Hz, 2H), 5.88 (d, J = 17.3 Hz, 2H), 5.32 (s, 1H), 4.92 (s, 1H), 4.30 (dd, J = 13.8, 3.0 Hz, 1H), 4.17 (t, J = 6.9 Hz, 2H), 4.07 (dd, J = 13.8, 8.0 Hz, 1H), 3.82 – 3.74 (m, 1H), 3.46 – 3.25 (m, 5H), 2.99 (dt, J = 14.3, 6.3 Hz, 4H), 1.89 (s, 2H), 1.29 (d, J = 44.9 Hz, 8H), 0.86 (s, 4H). ^{13}C NMR (100 MHz, DMSO-d_6) δ 123.64, 115.17, 70.11, 36.29, 31.49, 22.50, 13.97. HRMS (m/z) ESI+: [M]

calcd. for $C_{16}H_{31}N_4O_3$, 327.2391; found, 327.2365. ESI-: [M] calcd. for $C_2F_6NO_4S_2$, 279.9178; found, 279.9173.

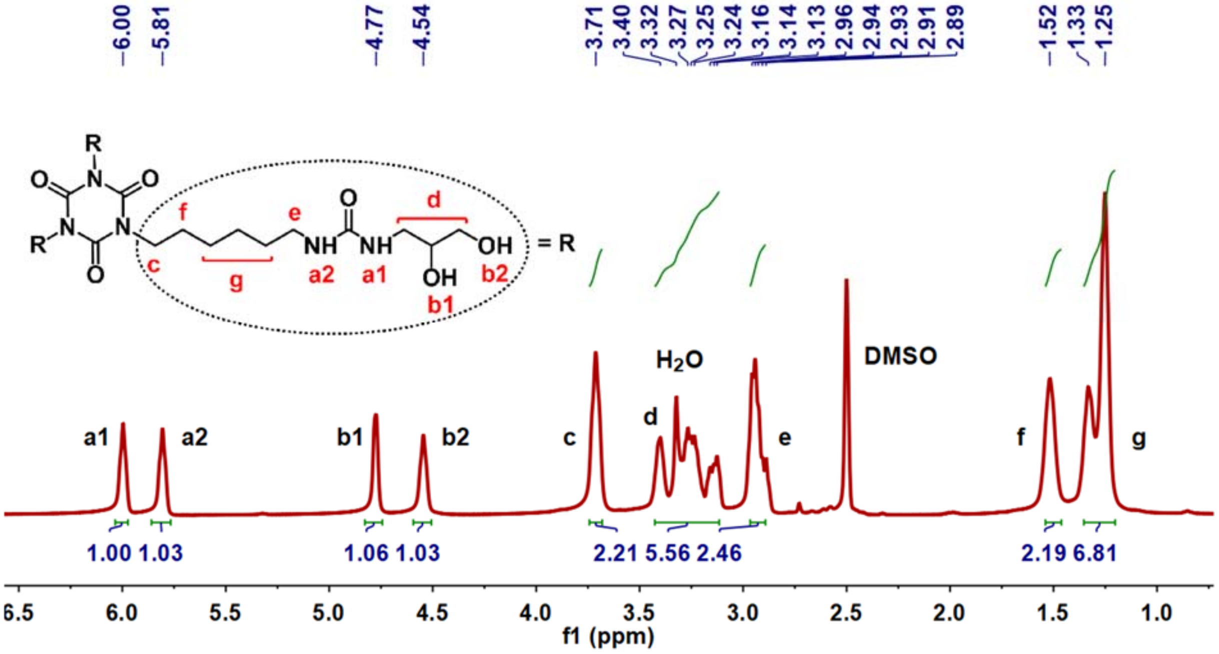

Supplementary Figure 1. ^{1}H NMR spectrum (400 MHz, 25 °C, DMSO-d_6) of Tri-OH.

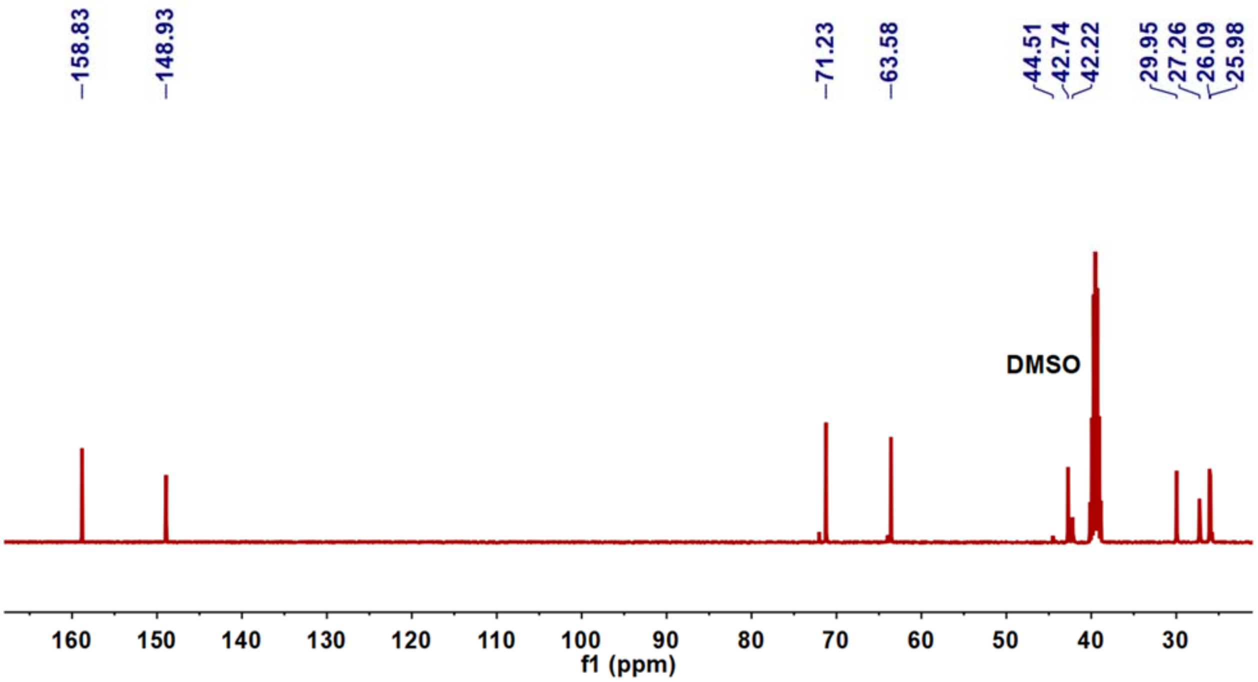

Supplementary Figure 2. ^{13}C NMR spectrum (100 MHz, 25 °C, DMSO-d_6) of Tri-OH.

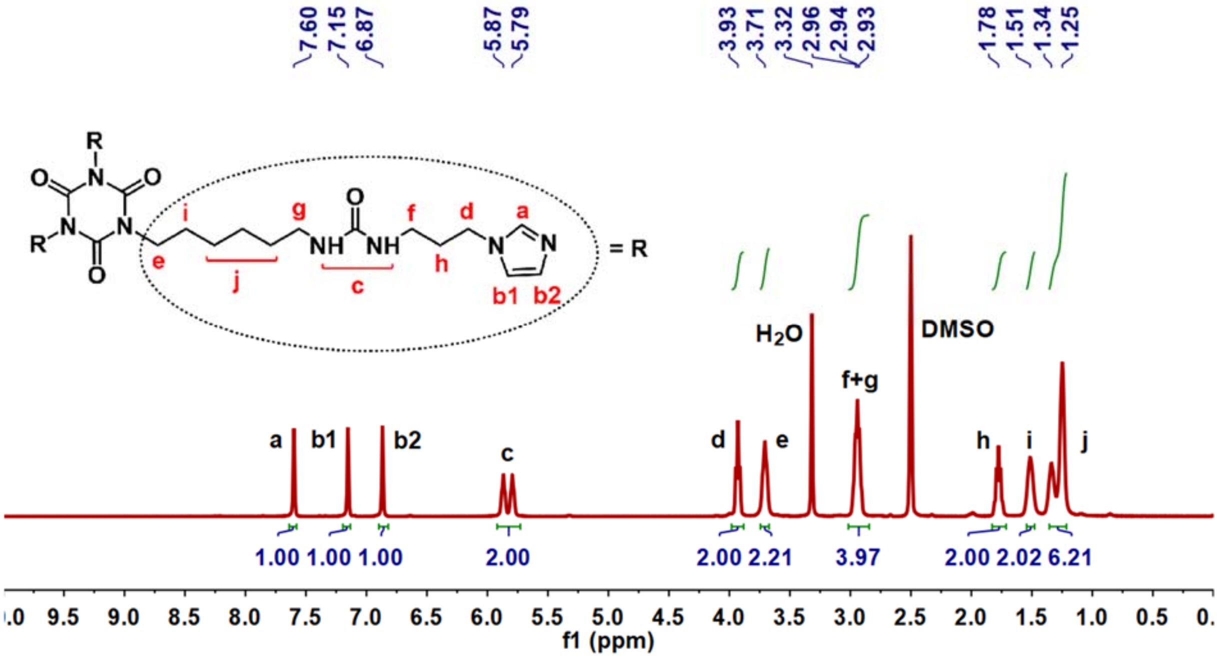

Supplementary Figure 3. ^{1}H NMR spectrum (400 MHz, 25 °C, DMSO-d_6) of Tri-Im.

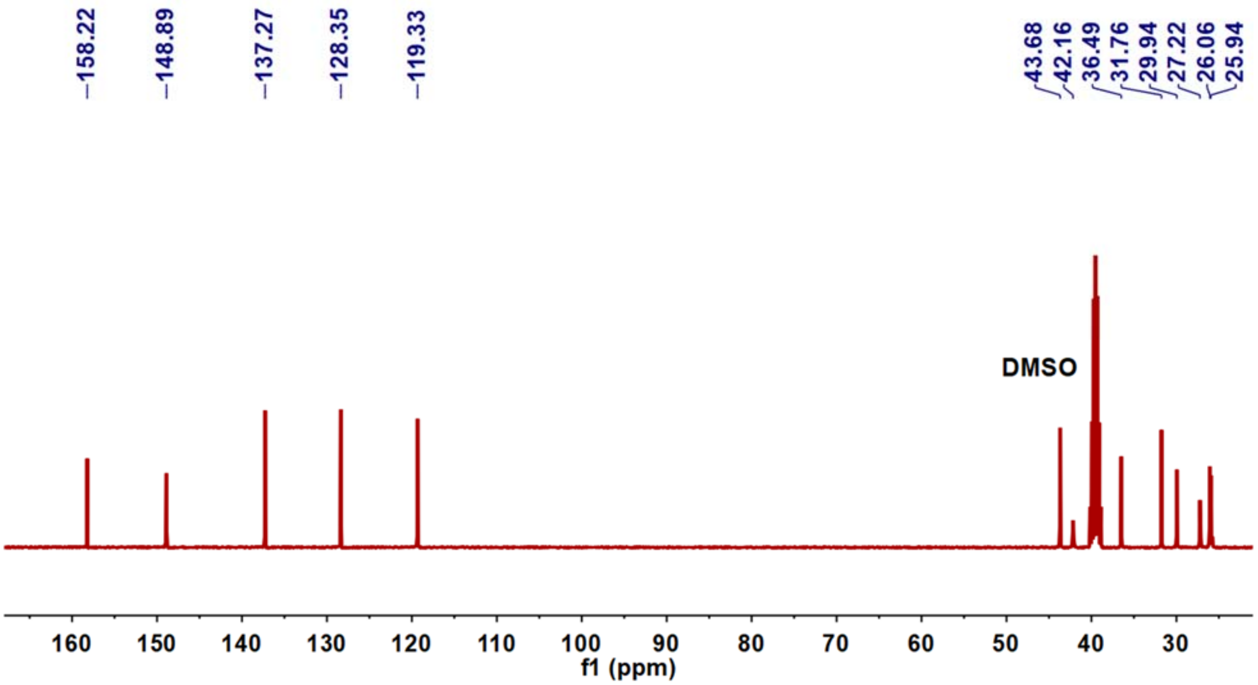

Supplementary Figure 4. ^{13}C NMR spectrum (100 MHz, 25 °C, DMSO-d_6) of Tri-Im.

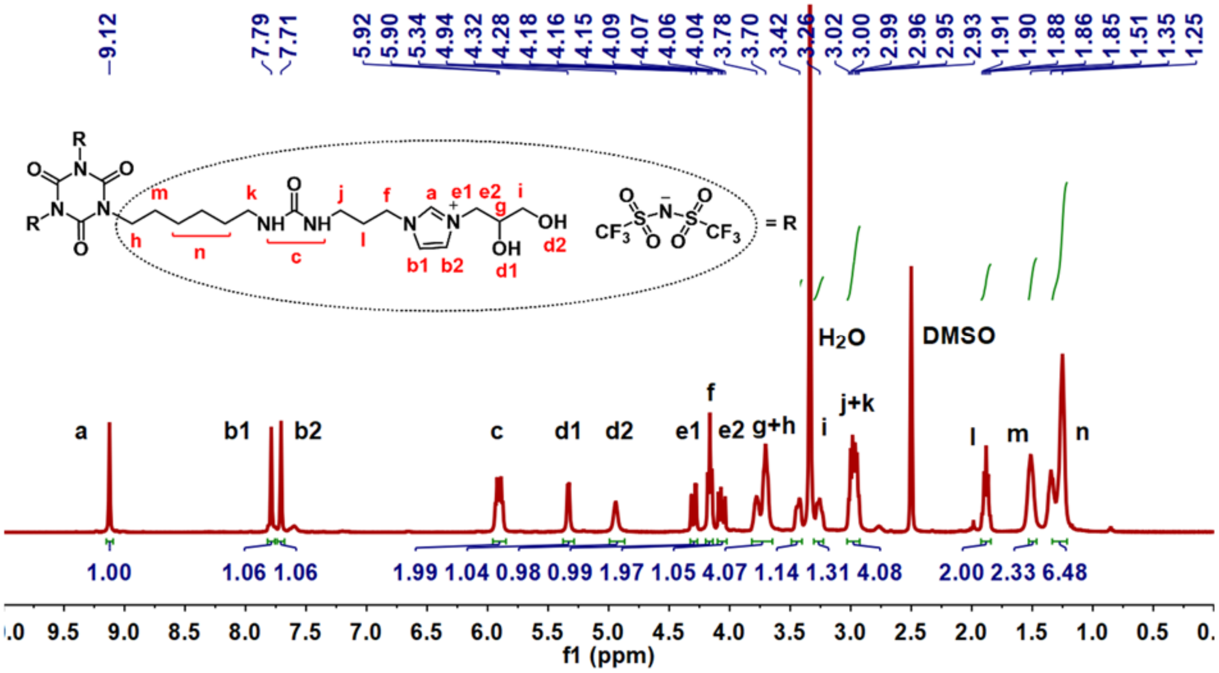

Supplementary Figure 5. ^{1}H NMR spectrum (400 MHz, 25 °C, DMSO-d_6) of Tri-HT.

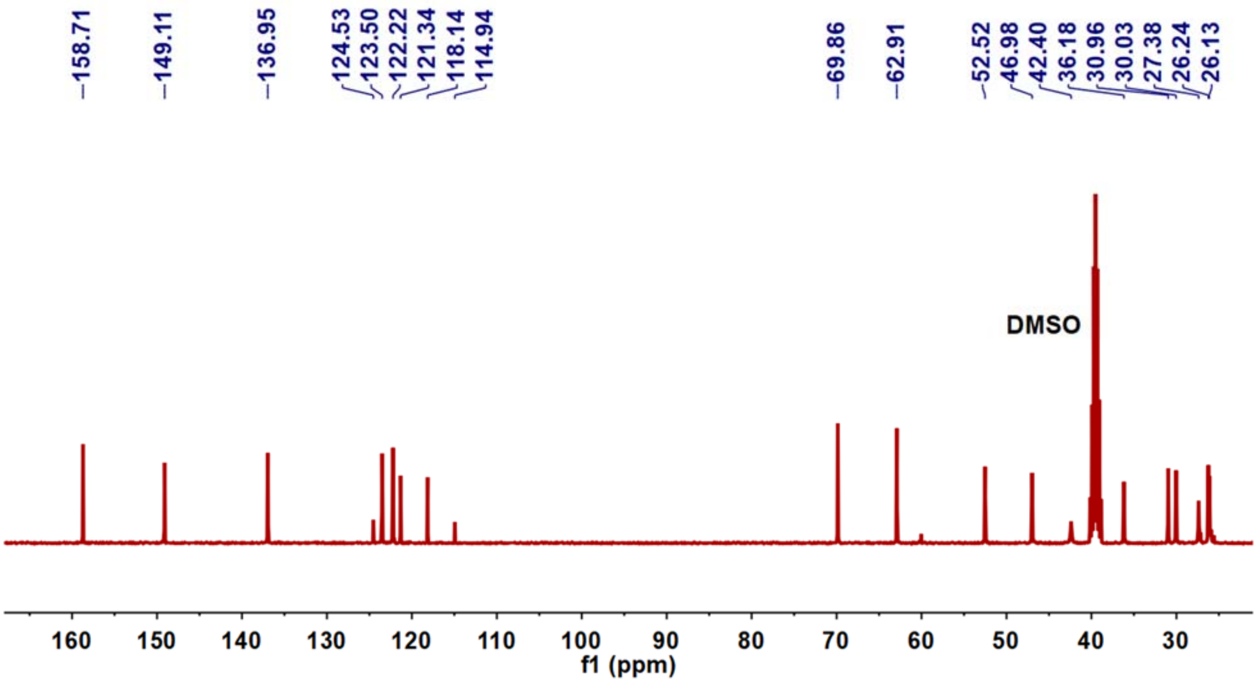

Supplementary Figure 6. ^{13}C NMR spectrum (100 MHz, 25 °C, DMSO-d_6) of Tri-HT.

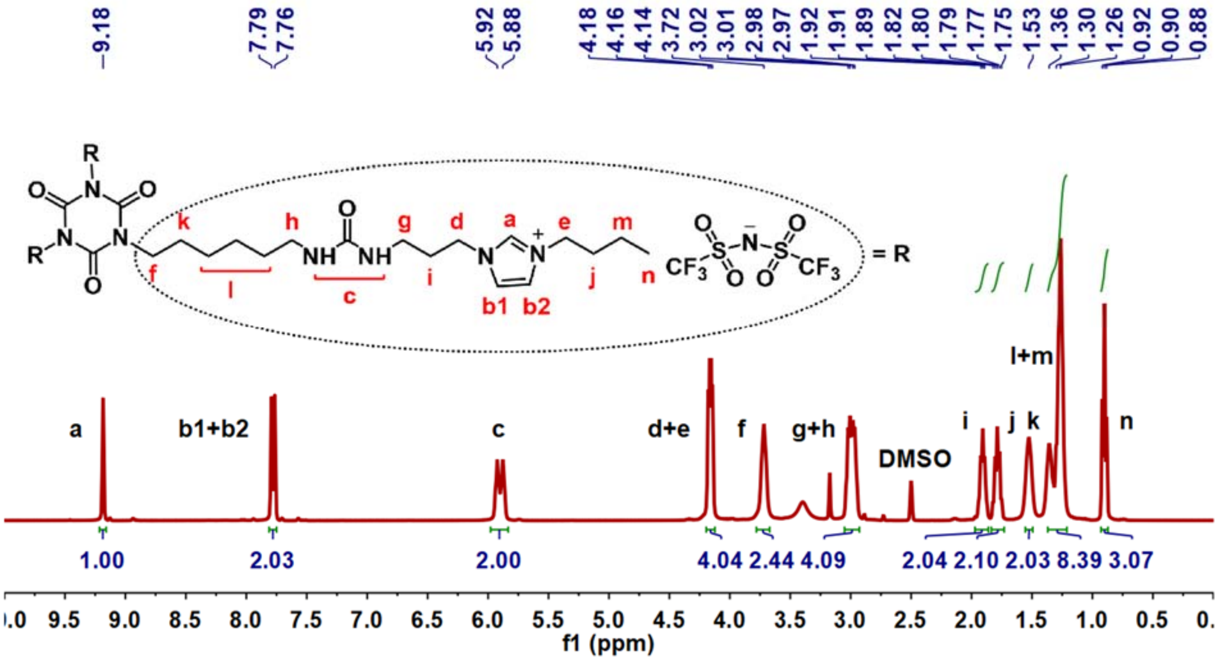

Supplementary Figure 7. ^1H NMR spectrum (400 MHz, 25 °C, DMSO-d_6) of Tri-AT.

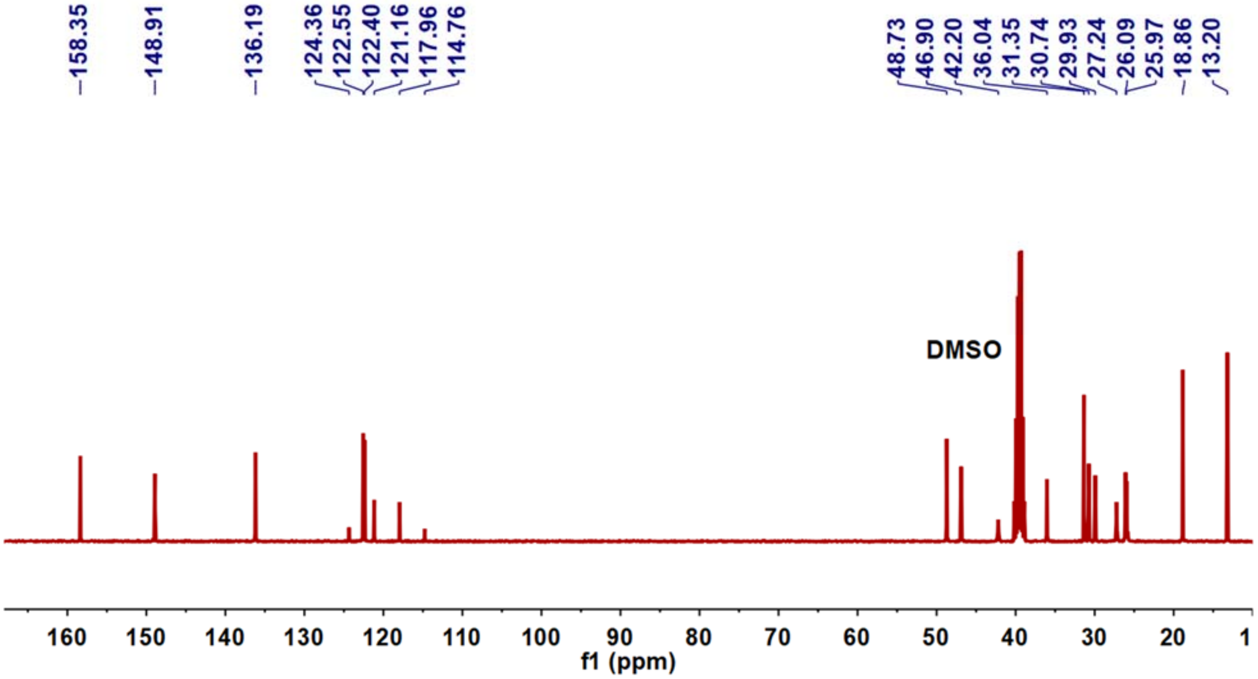

Supplementary Figure 8. ^{13}C NMR spectrum (100 MHz, 25 °C, DMSO-d_6) of Tri-AT.

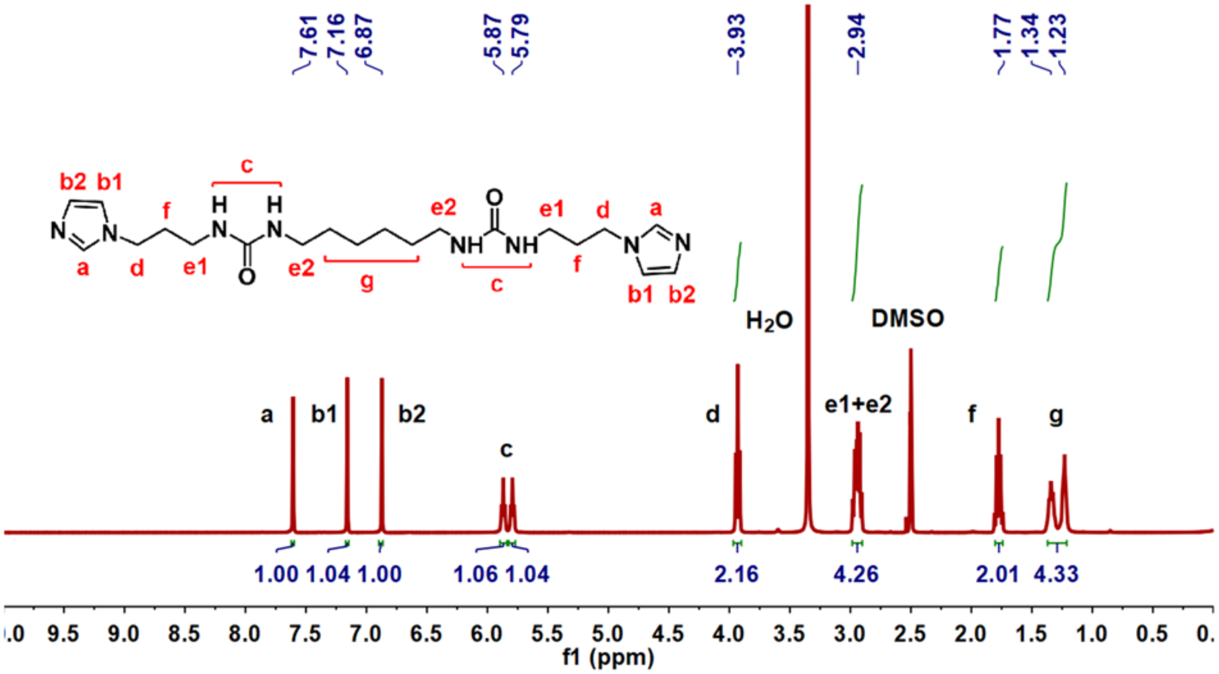

Supplementary Figure 9. ^{1}H NMR spectrum (400 MHz, 25 °C, DMSO-d_6) of Bis-Im.

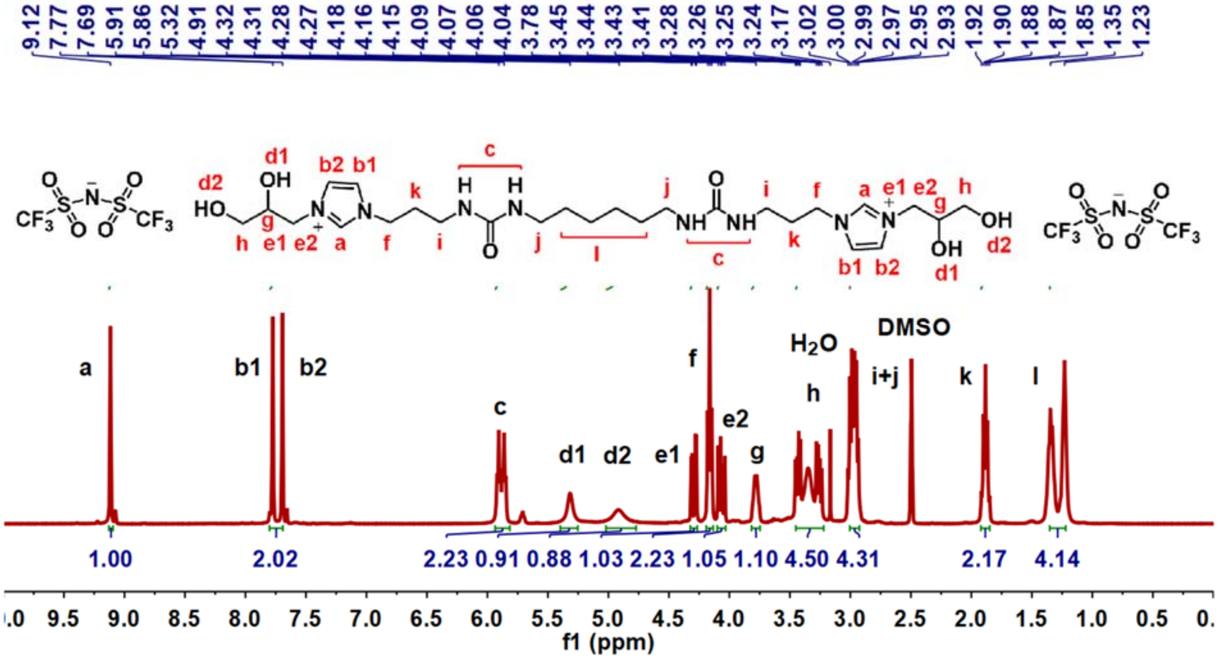

Supplementary Figure 10. ^{1}H NMR spectrum (400 MHz, 25 °C, DMSO-d_6) of Bis-HT.

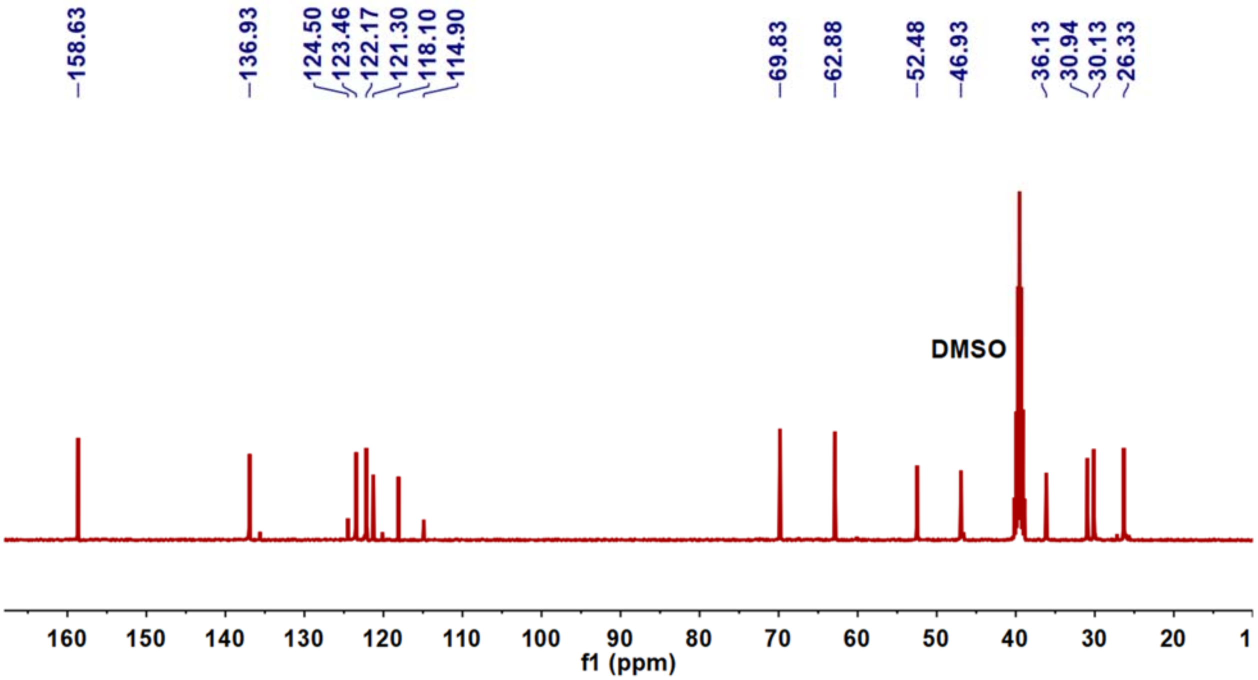

Supplementary Figure 11. ^{13}C NMR spectrum (100 MHz, 25 °C, DMSO-d_6) of Bis-HT.

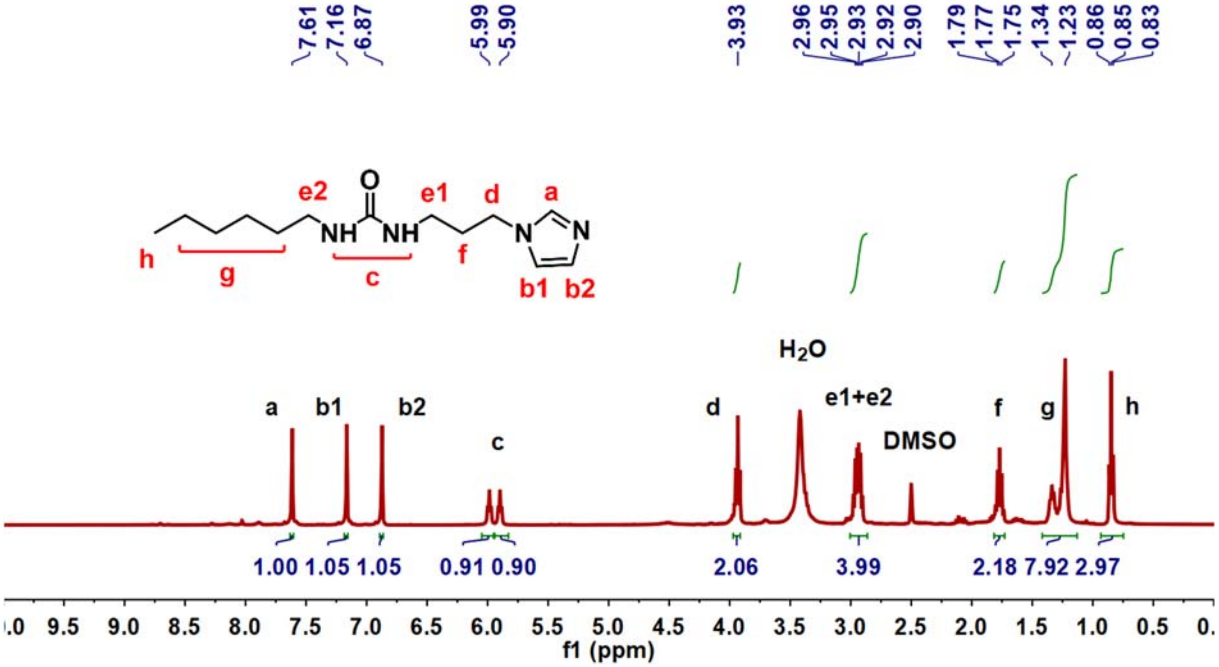

Supplementary Figure 12. ^{1}H NMR spectrum (400 MHz, 25 °C, DMSO-d_6) of Mon-Im.

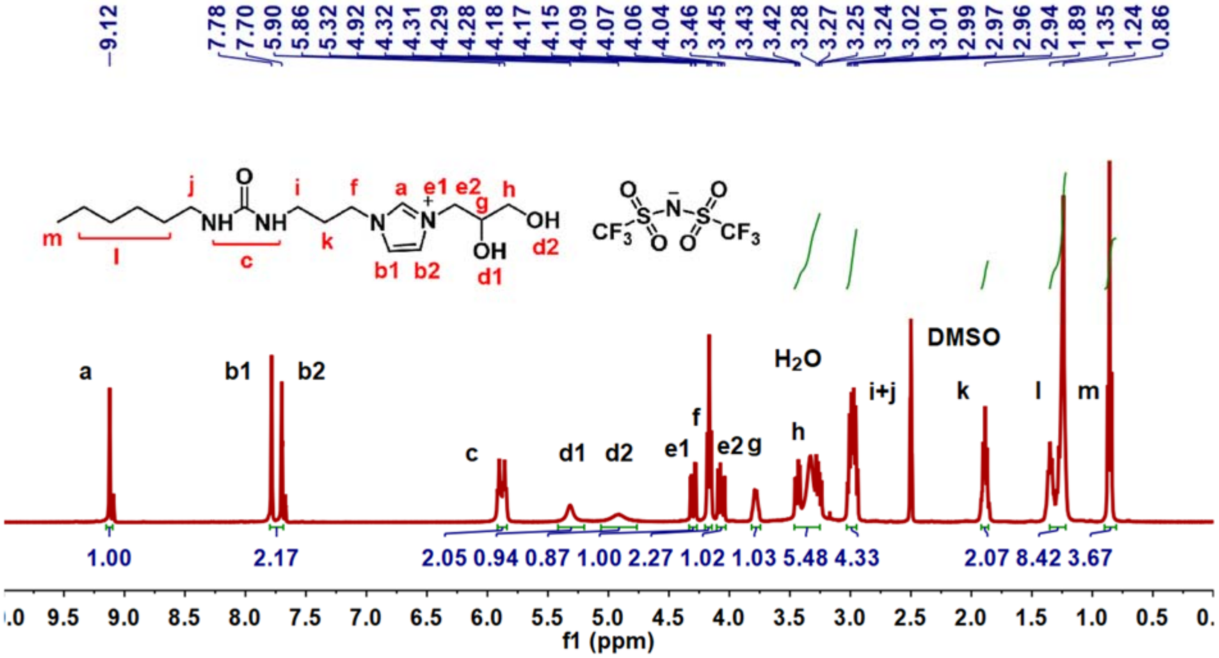

Supplementary Figure 13. ^{1}H NMR spectrum (400 MHz, 25 °C, DMSO-d_6) of Mon-HT.

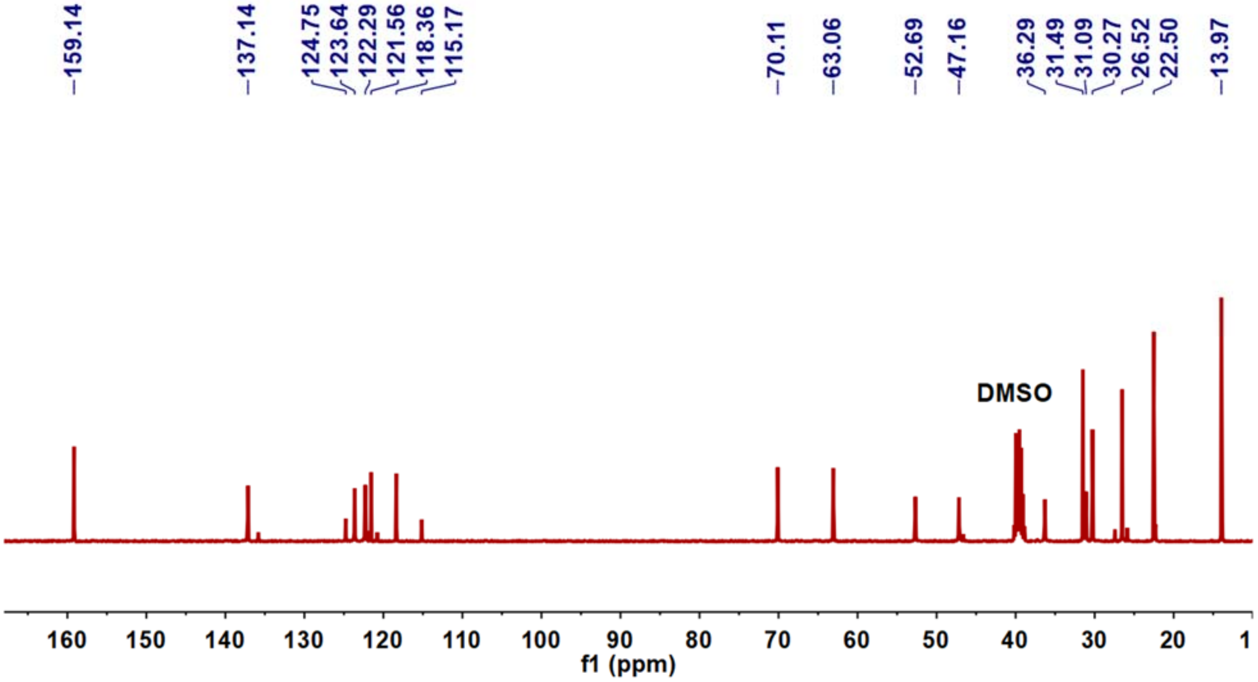

Supplementary Figure 14. ^{13}C NMR spectrum (100 MHz, 25 °C, DMSO-d_6) of Mon-HT.

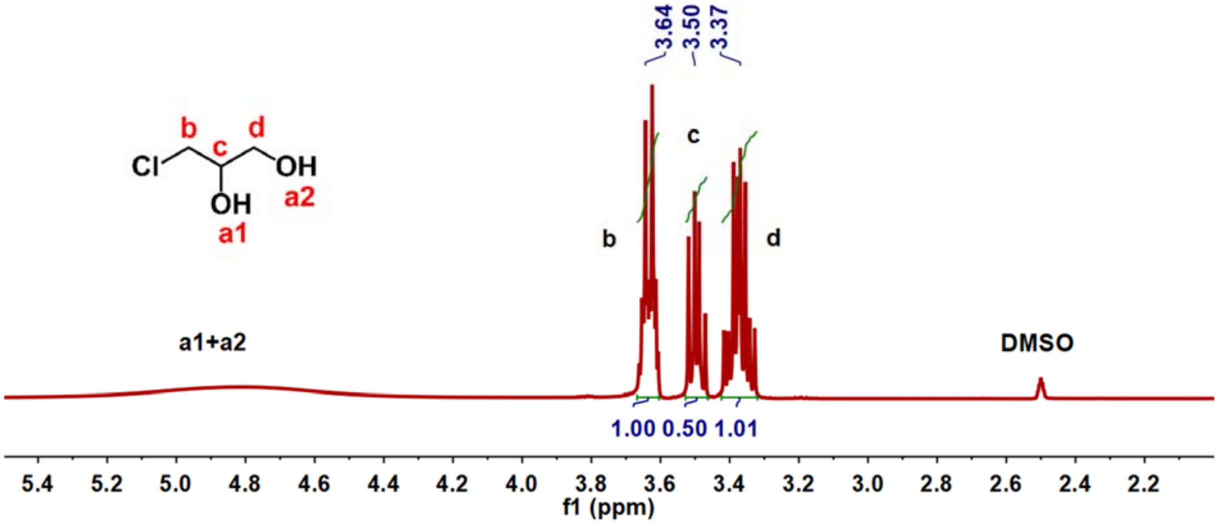

Supplementary Figure 15. ^{1}H NMR spectrum (400 MHz, 25 °C, DMSO-d_6) of 3-chloro-1, 2-propanediol.

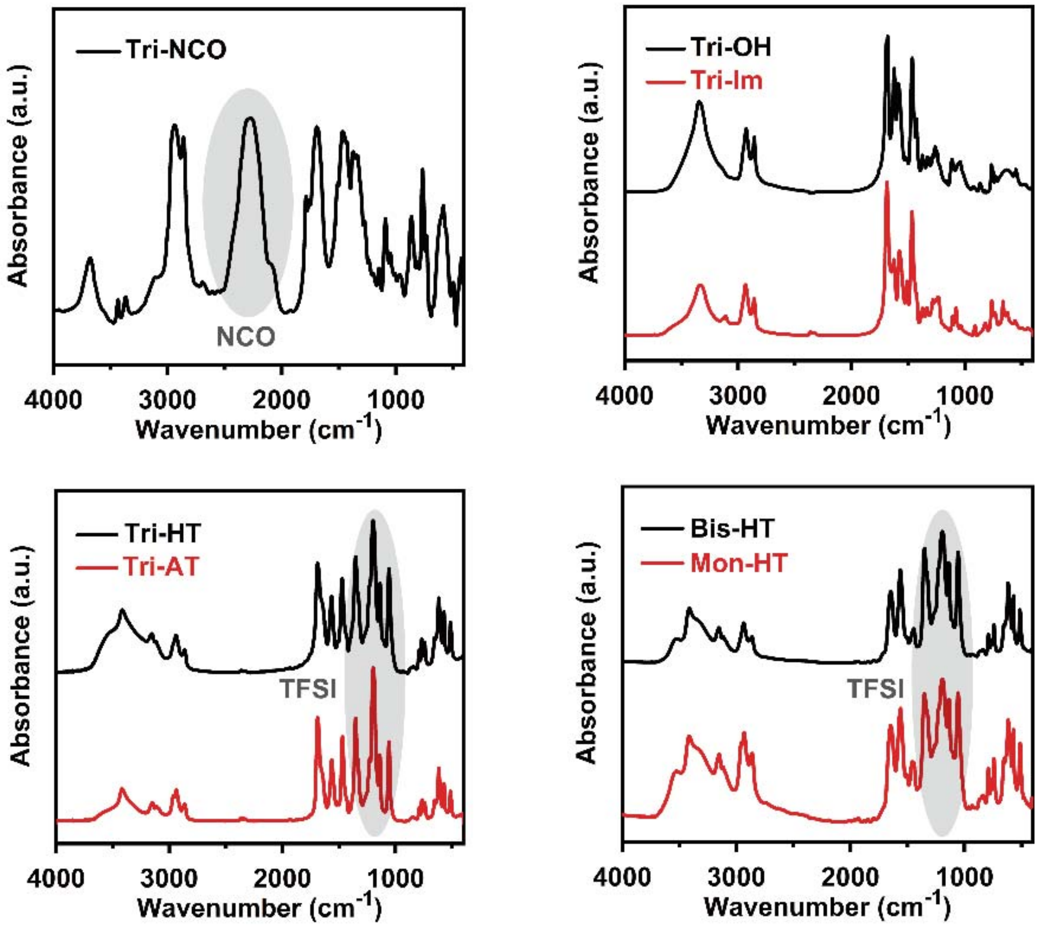

Supplementary Figure 16. FT-IR spectra of Tri-HT and reference compounds. Corresponding peaks indicated the successful synthesis of Tri-HT and reference compounds.

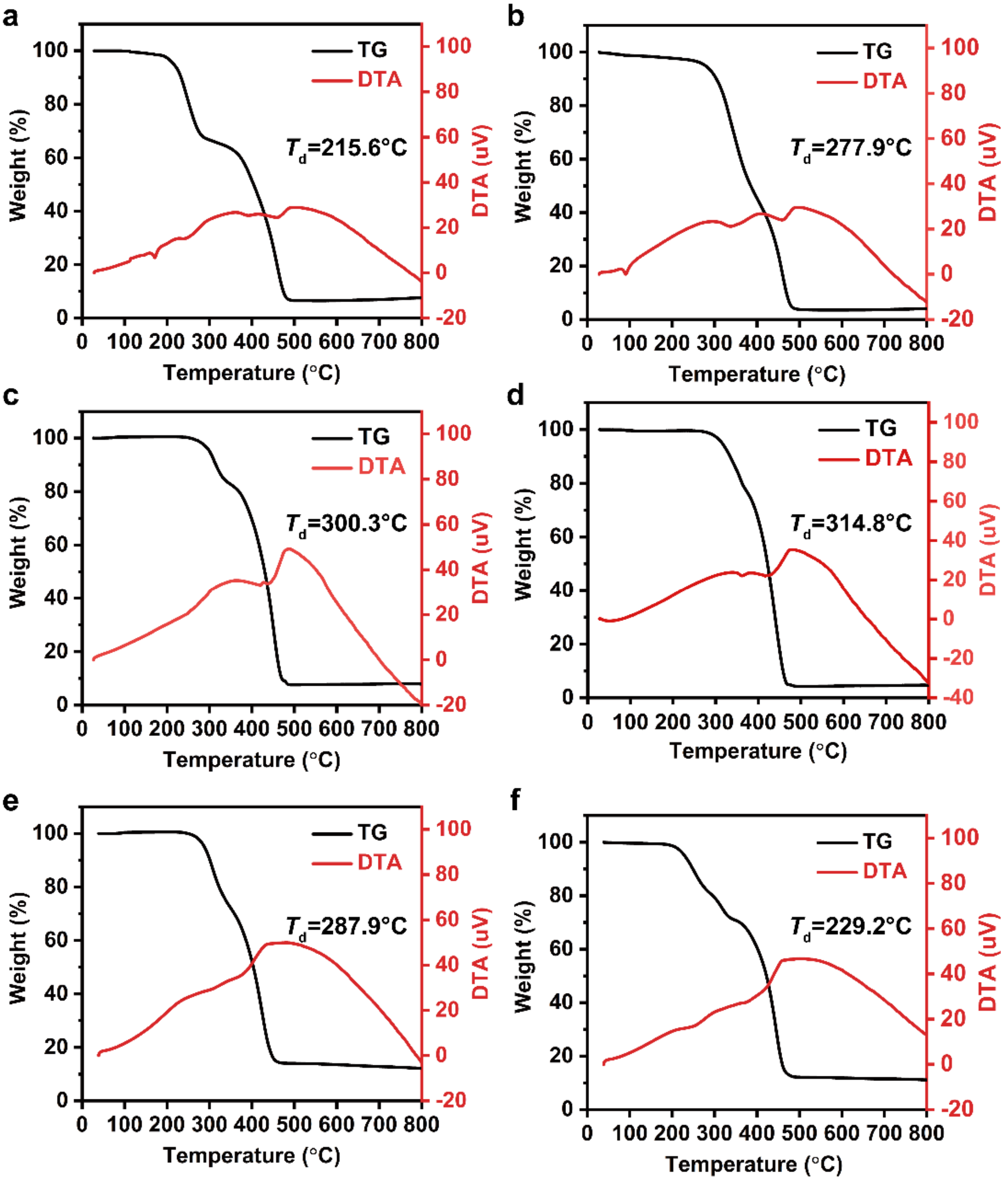

Supplementary Figure 17. TGA curves of Tri-HT and reference compounds. (a) Tri-OH, (b) Tri-Im, (c) Tri-HT, (d) Tri-AT, (e) Bis-HT, (f) Mon-HT (T_d is defined as the decomposition temperature when 5% weight loss). Compared with Mon-HT, the T_d of Tri-HT and Bis-HT were highly enhanced.

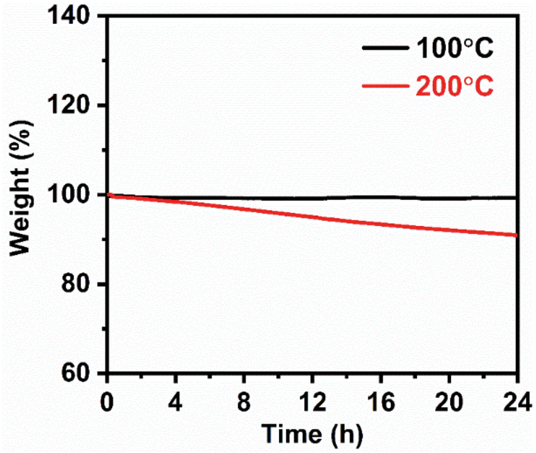

Supplementary Figure 18. Isothermal TGA curves of Tri-HT at 100 °C and 200 °C.

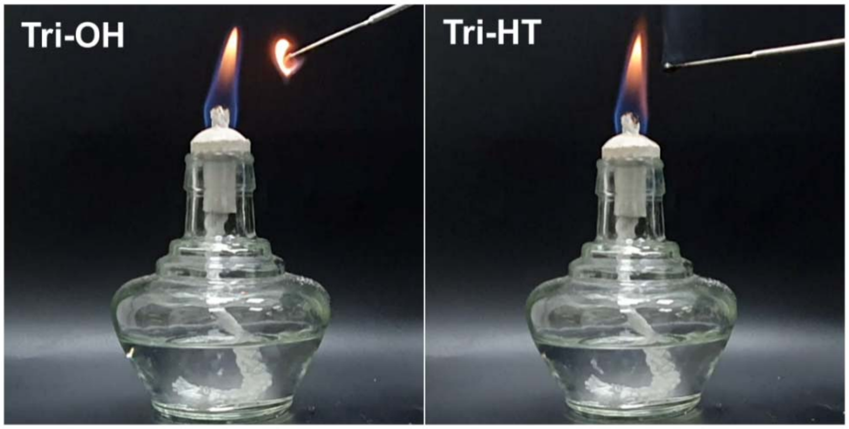

Supplementary Figure 19. Photographs of Tri-HT and Tri-OH ignited by flames.

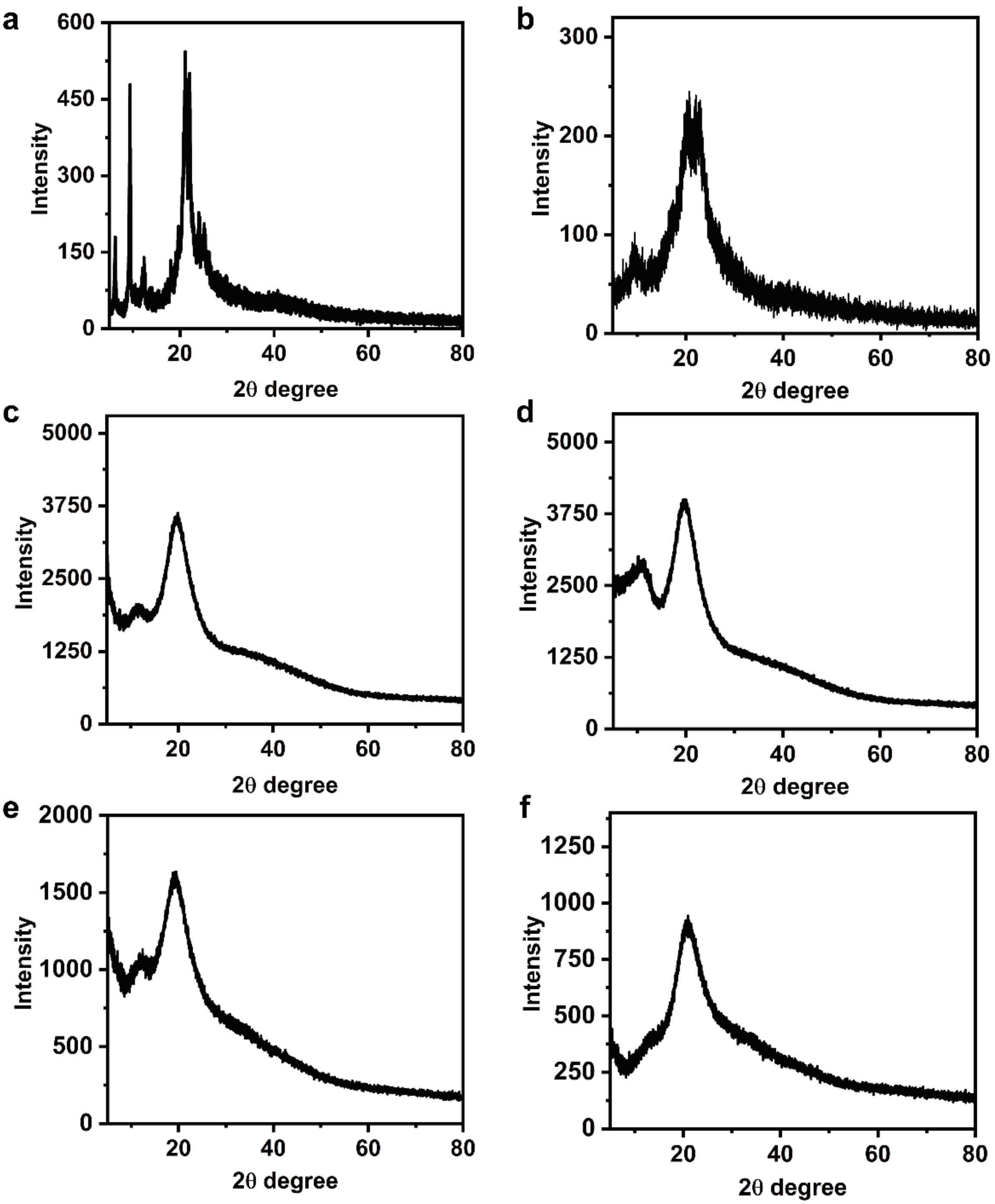

Supplementary Figure 20. XRD diffractogram of Tri-HT and reference compounds. (a) Tri-OH, (b) Tri-Im, (c) Tri-HT, (d) Tri-AT, (e) Bis-HT, (f) Mon-HT.

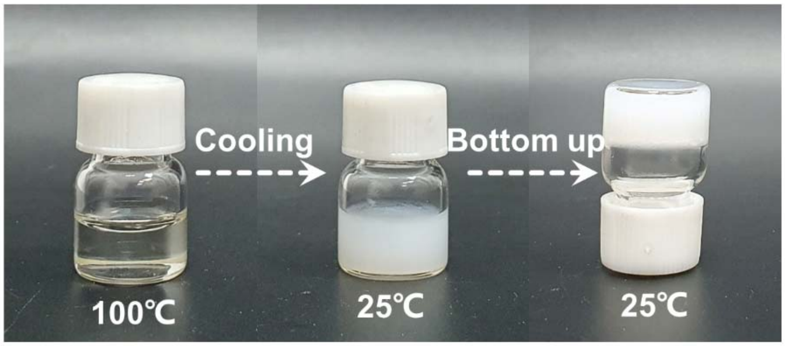

Supplementary Figure 21. Organogel forms at a gelator (Tri-OH) loading of 6 wt% (solvent: DMF). A certain amount of Tri-HT was dissolved in DMF by gentle heating. After it was dissolved and cooling down, organogel was obtained.

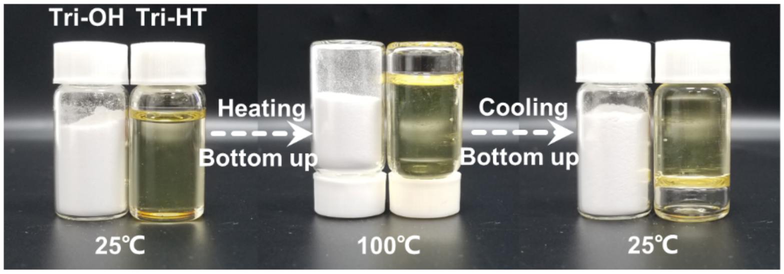

Supplementary Figure 22. Photographs of Tri-OH and Tri-HT at different temperatures. By simply using bottom-up during the heating/cooling process, it was observed that Tri-HT undergoes a process of liquefied and re-solidification. While there are no obvious state changes for Tri-OH.

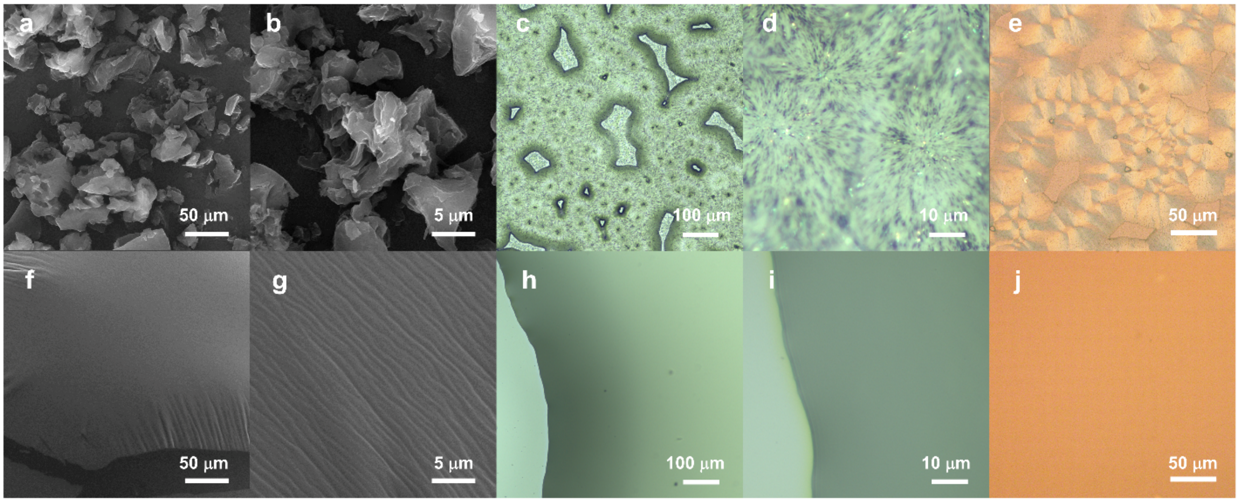

Supplementary Figure 23. Microscopic state differences of Tri-OH and Tri-HT. SEM images of (a and b) Tri-OH, (f and g) Tri-HT. LM (reflection mode) images of (c and d) Tri-OH, (h and i) Tri-HT. POM images of (e) Tri-OH, (j) Tri-HT.

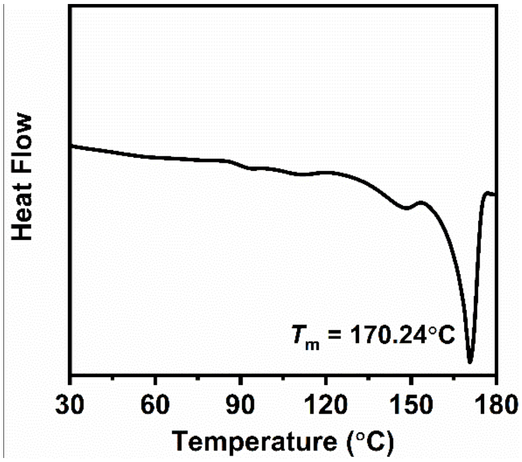

Supplementary Figure 24. DSC curve of Tri-OH.

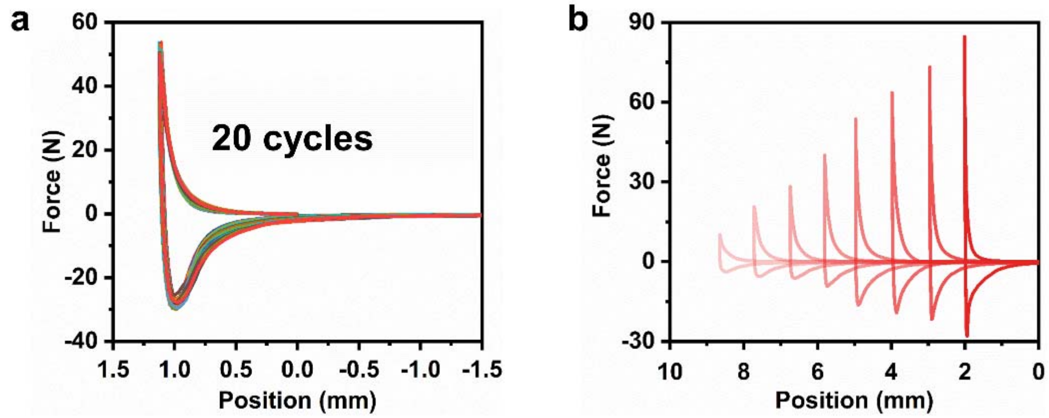

Supplementary Figure 25. Probe-tack test on the surface of Tri-HT layer. (a) 20 times successive test under a preload force of 50 N. (b) Probe-tack test under different preload forces.

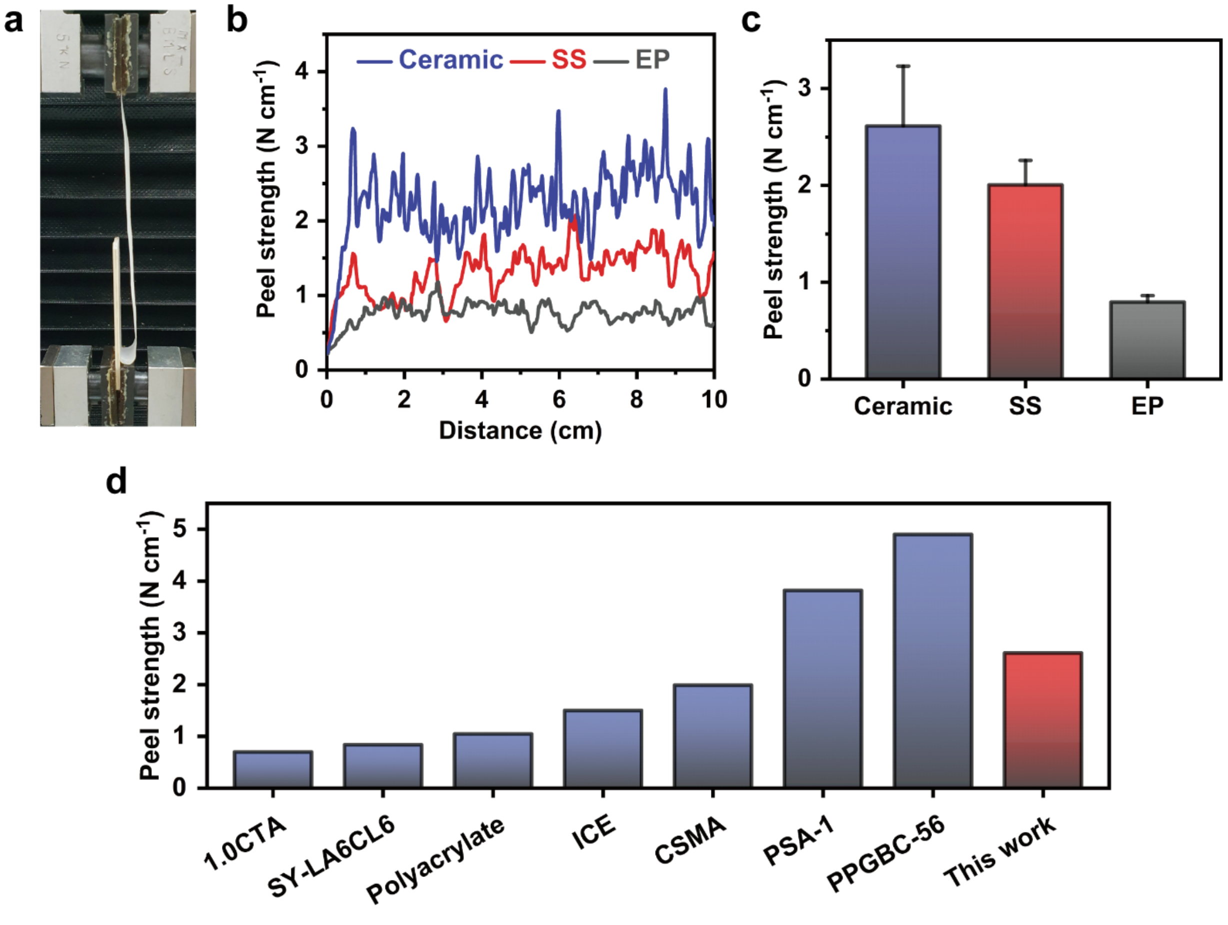

Supplementary Figure 26. 180° peeling test (speed: 360 mm min^{-1}) on different substrates. (a) 180° peeling test on ceramic substrate, (b) obtained peeling curves, and (c) average peel strength. Tri-HT was pre-coated on A4 paper from one side at 100 °C. After cooling to room temperature, pressing the paper tape on a ceramic substrate from another side by hand and start testing. All tests were carried out at 25 °C. (d) Comparison of the adhesion properties of Tri-HT and reported pressure-sensitive adhesives, such as 1.0CTA[1], SY-LA6CL6[2], Polyacrylate[3], ICE[4], CSMA[5], PSA-1[6], and PPGBC-56[7].

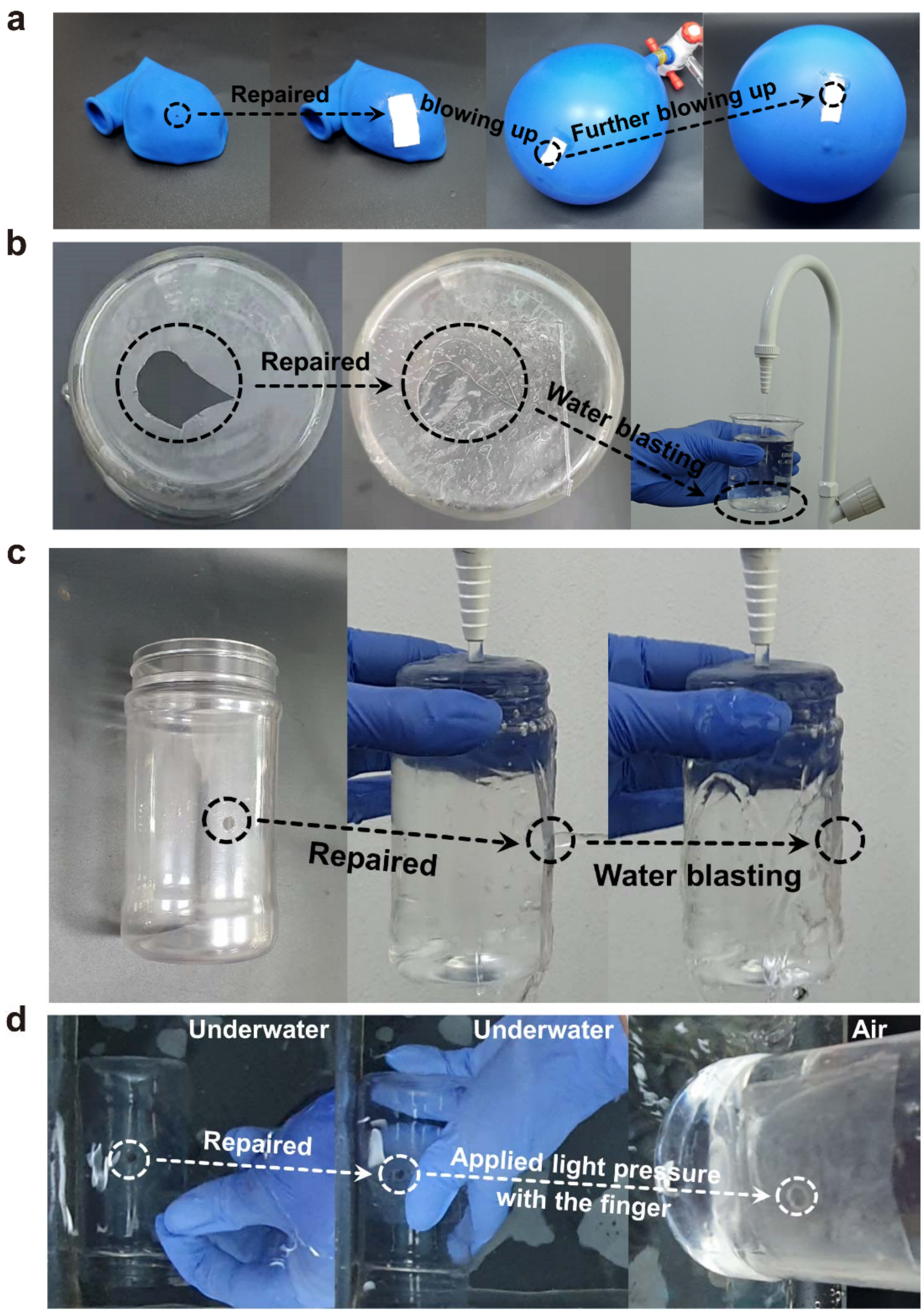

Supplementary Figure 27. Macroscopic adhesion. (a) Damaged balloon with a hole of 1~2 mm diameter and the hole completely repaired by a rubber sheet pre-coated with Tri-HT. In the following air-blowing process, no leakage was found. (b) Broken beaker and (c) damaged bottle repaired by a polyethylene film pre-coated with Tri-HT, and corresponding holes were sealed and

no water flows out again (hydraulic pressure: ~0.20 MPa). Besides, due to the water-insoluble nature of Tri-HT, direct underwater adhesion (d) is also feasible.

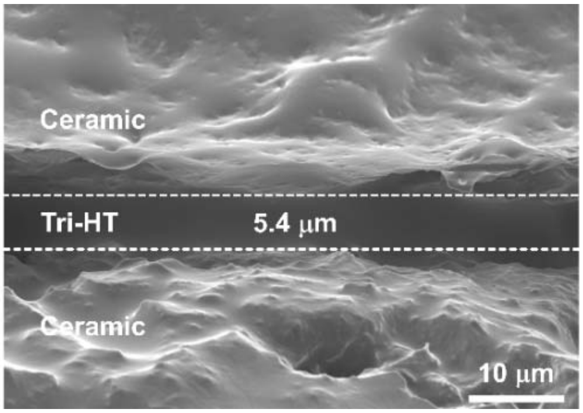

Supplementary Figure 28. The thickness of two adhered ceramic substrates using Tri-HT.

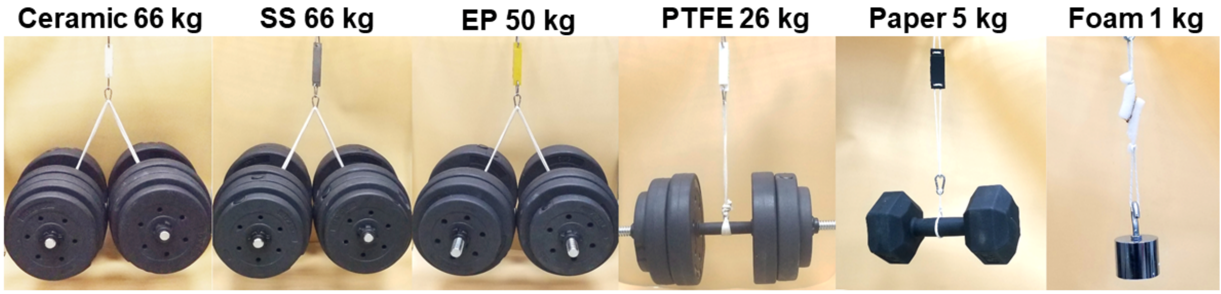

Supplementary Figure 29. Macroscopic adhesion tests of Tri-HT on various substrates under ambient conditions (adhesion areas are 12, 15, 12, 12, 10, and 10 cm^2 on ceramic, SS, EP, PTFE, paper, and foam, respectively).

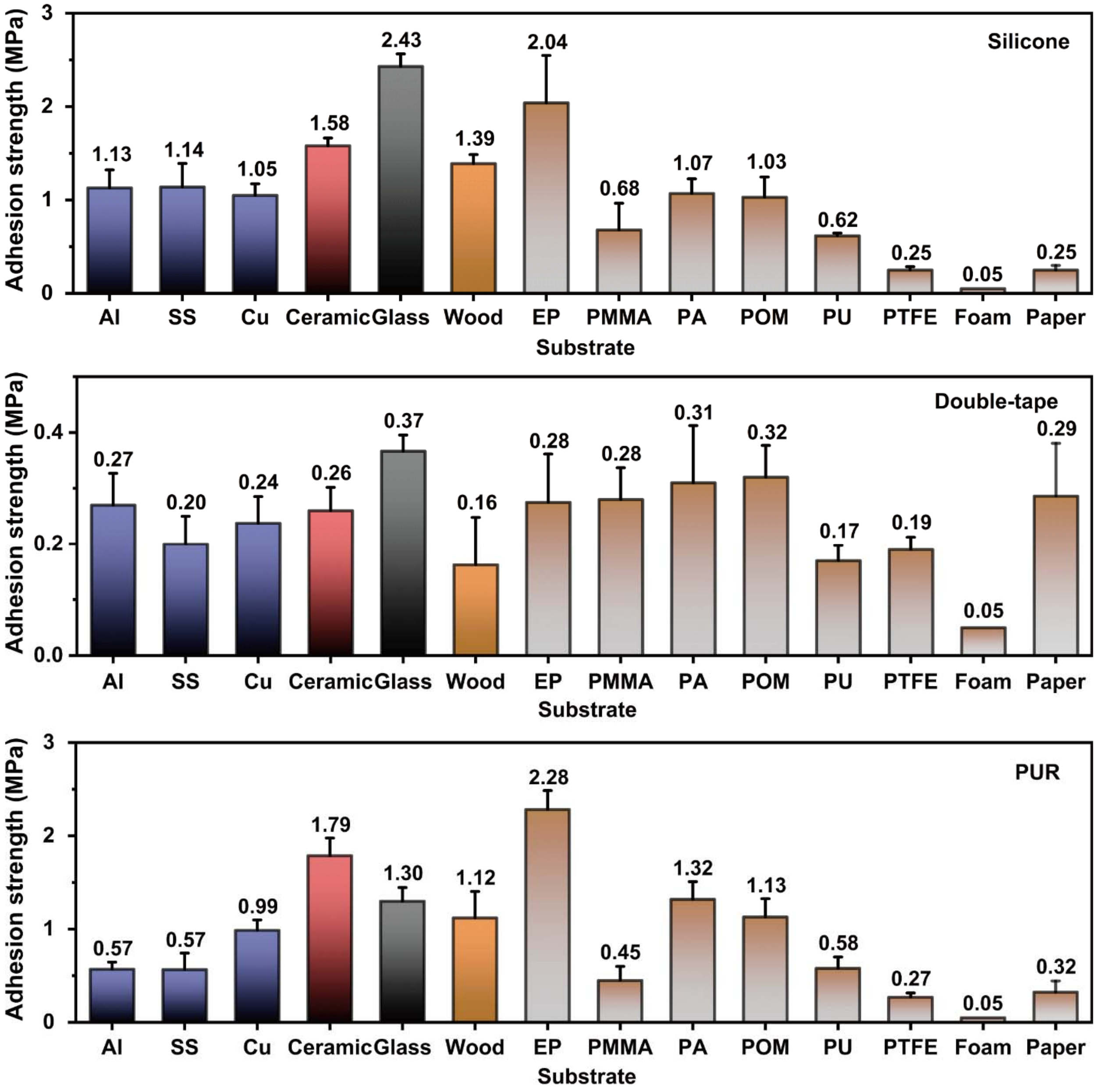

Supplementary Figure 30. Adhesion strengths of commercially available adhesives. Silicone, double-tape, and polyurethane resin (PUR) adhesives are bought from 3M. All tests were carried out at 25 °C. Error bars are standard deviation for n=3~5 measurements.

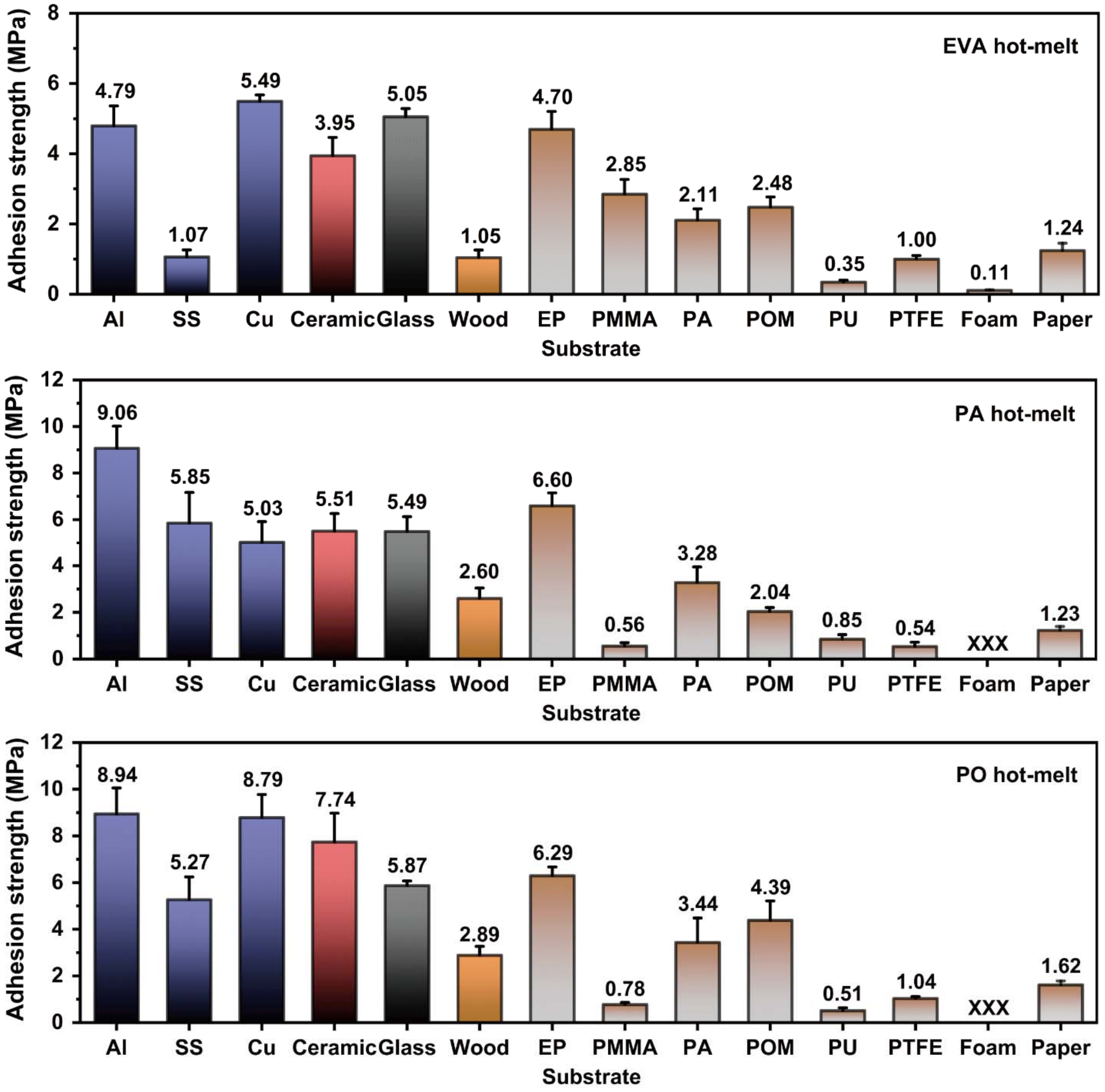

Supplementary Figure 31. Adhesion strengths of commercially available hot melt adhesives including poly(ethylene-co-vinyl acetate (EVA), polyamide (PA), and polyolefins (PO). It should be noted that a high temperature of 130 ºC was required to soften and melt PA and PO for adhesion. All tests were carried out at 25 °C. Error bars are standard deviation for n=3~5 measurements.

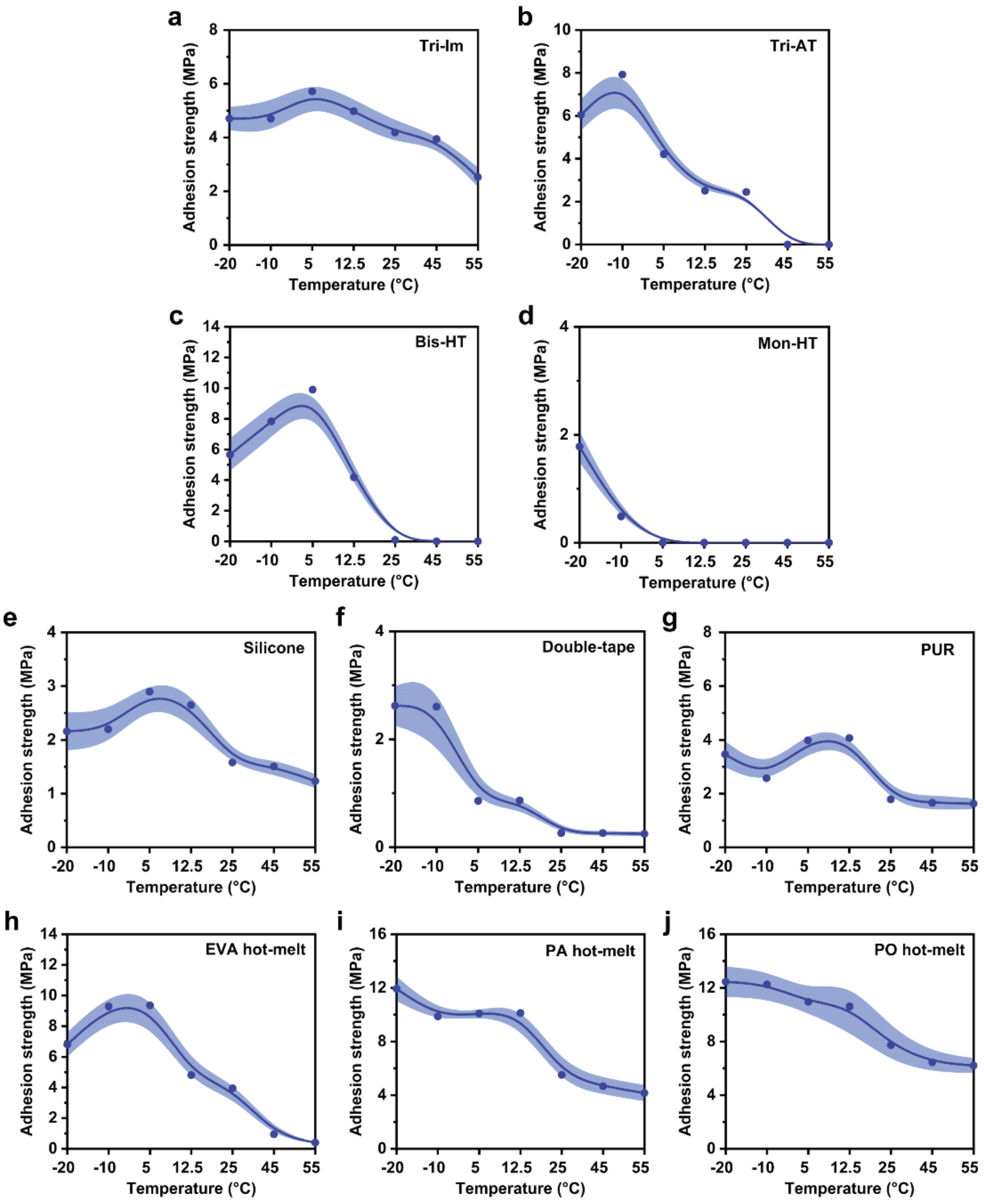

Supplementary Figure 32. Temperature-dependent adhesion strength of investigated adhesives on ceramic substrate. (a) Tri-Im, (b) Tri-AT, (c) Bis-HT, (d) Mon-HT, (e) silicone, (f) double-tape,

(g) Polyurethane (PUR), (h) poly(ethylene-co-vinyl acetate) (EVA) hot melt, (i) Polyamide (PA) hot melt, (j) Polyolefins (PO) hot melt.

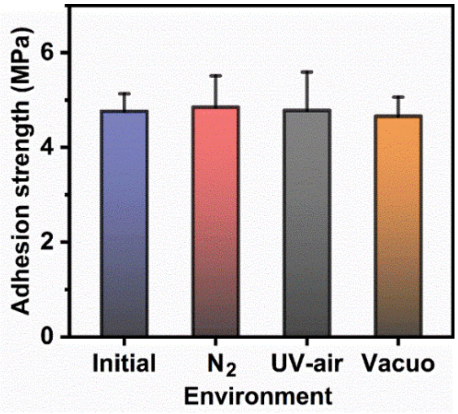

Supplementary Figure 33. Adhesion strength of adhered glass substrate after storing in different environmental conditions for 1 month. All tests were carried out at 25 °C. Error bars are standard deviation for n=3~5 measurements.

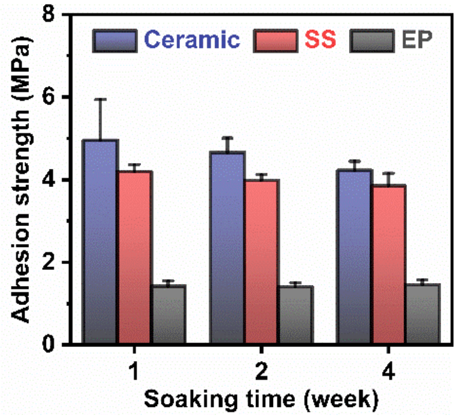

Supplementary Figure 34. Adhesion strengths of Tri-HT soaking in deionized water for 1, 2, and 4 weeks. All tests were carried out at 25 °C. Error bars are standard deviation for n=3~5 measurements.

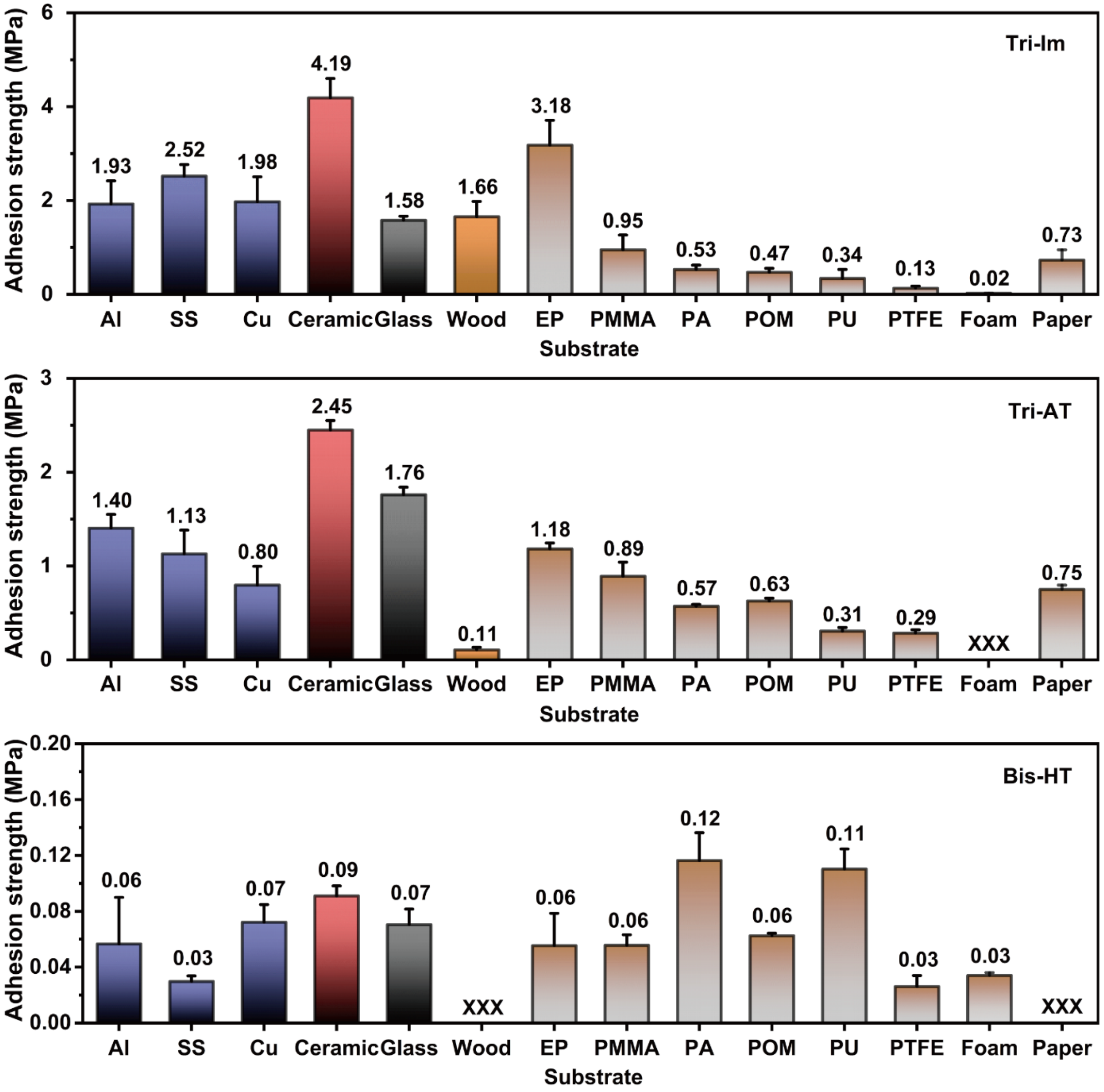

Supplementary Figure 35. Adhesion strengths of reference compounds (e.g., Tri-Im, Tri-AT and Bis-HT). All tests were carried out at 25 °C. Error bars are standard deviation for n=3~5 measurements (XXX represents no observable adhesion).

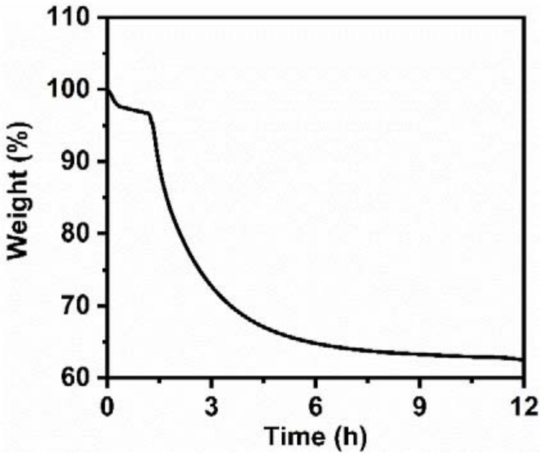

Supplementary Figure 36. Isothermal TGA curves of Tri-OH at 170°C.

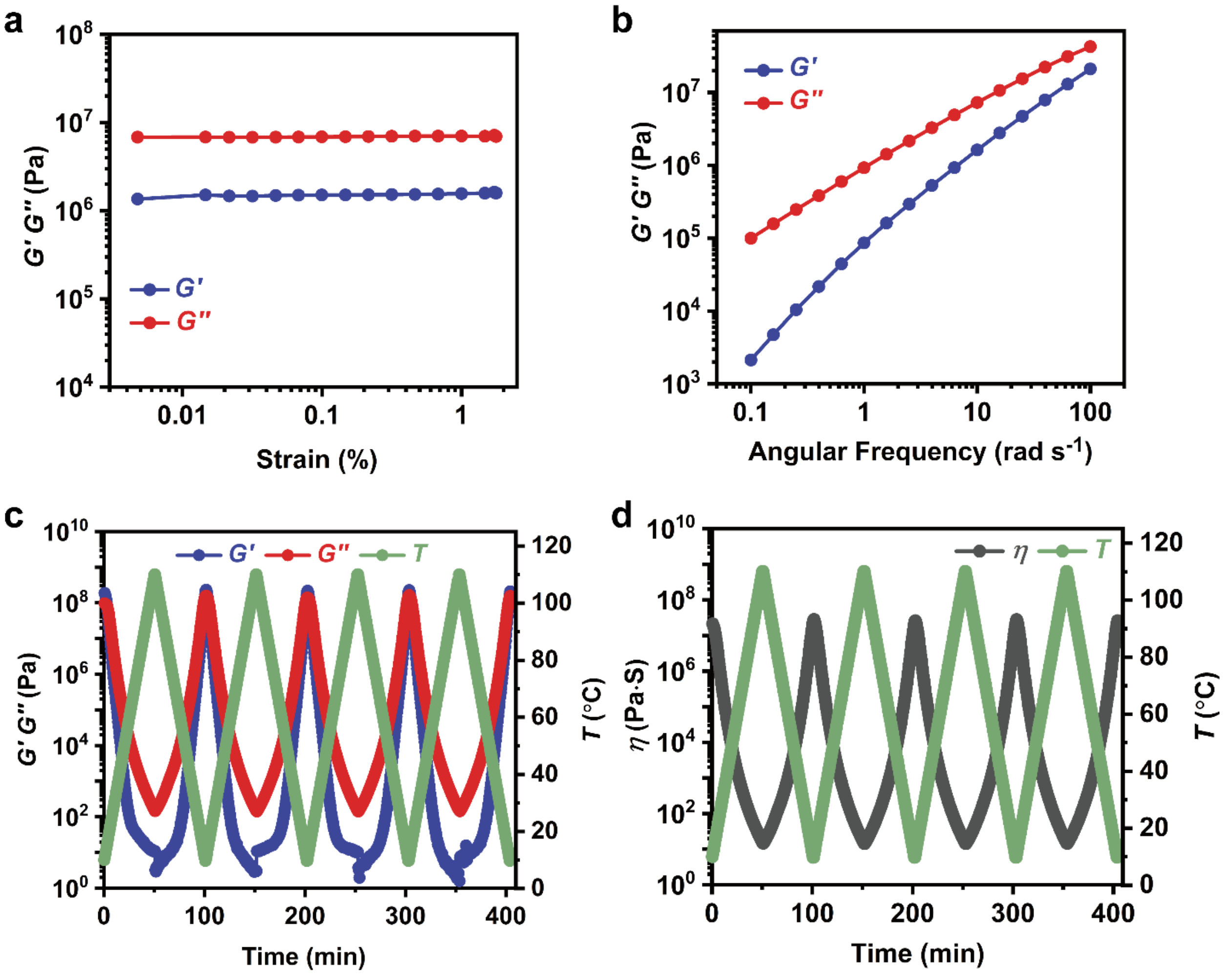

Supplementary Figure 37. Rheological measurements of Tri-Im. The angular frequency for strain sweep (a) is 10 rad s^{-1} and the strain for the dynamic frequency sweep (b) is 1% (temperature: 25 ºC). (c) Storage modulus (*G'*), loss modulus (*G''*), and (d) complex viscosity (*η*) values at reversible temperature-dependent rheological tests.

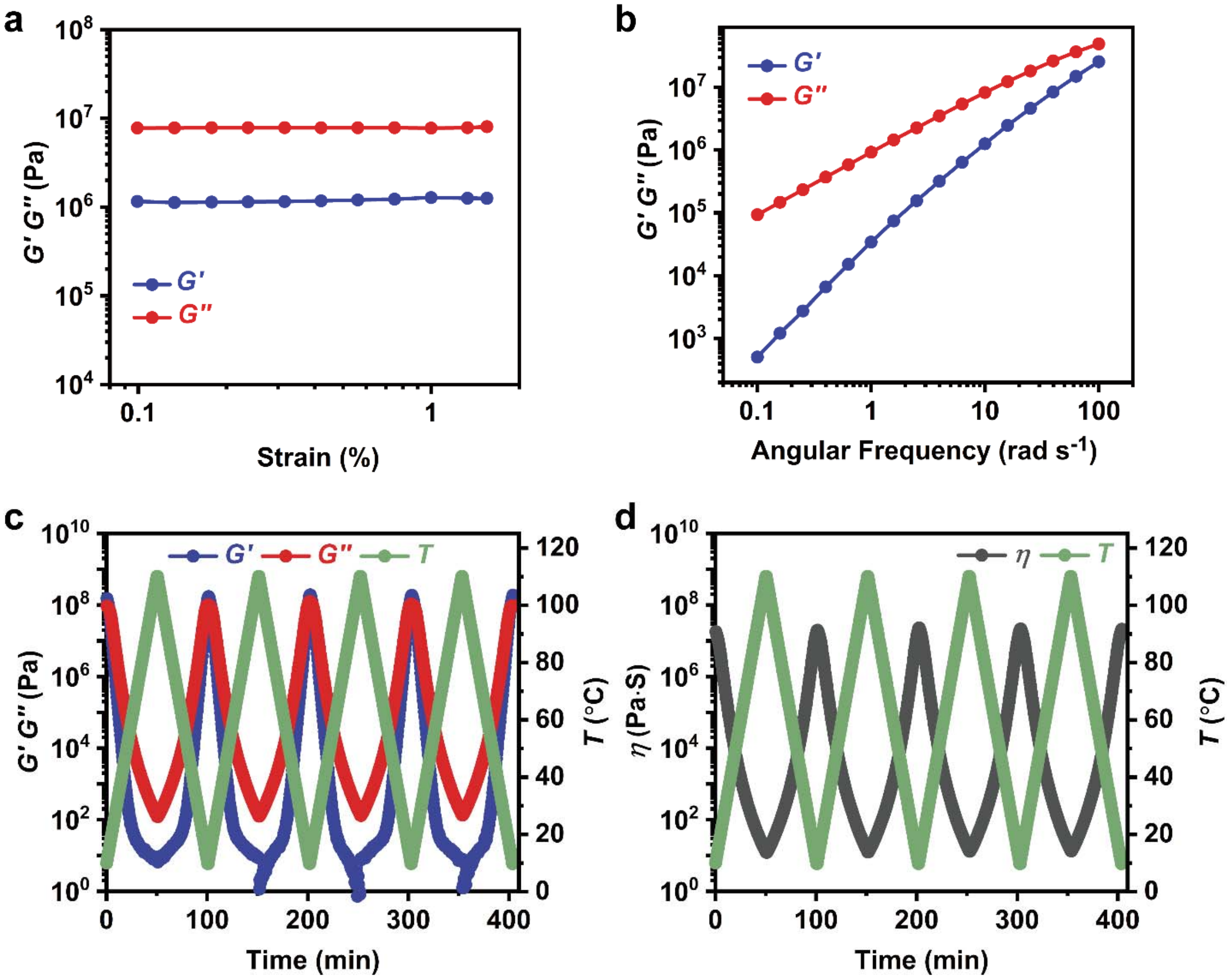

Supplementary Figure 38. Rheological measurements of Tri-HT. The angular frequency for strain sweep (a) is 10 rad s^{-1} and the strain for the dynamic frequency sweep (b) is 1% (temperature: 25 ºC). (c) Storage modulus (*G'*), loss modulus (*G''*), and (d) complex viscosity (*η*) values at reversible temperature-dependent rheological tests.

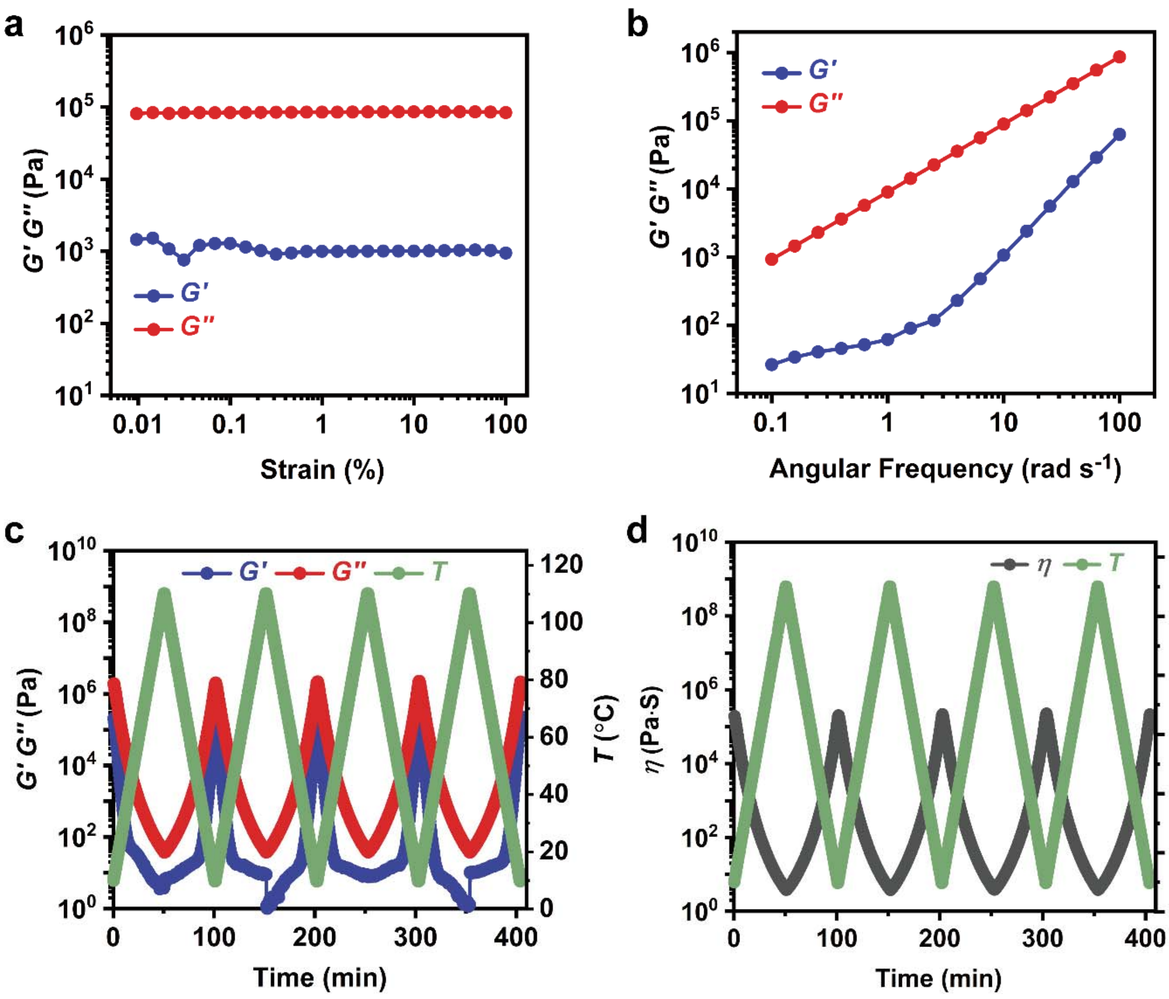

Supplementary Figure 39. Rheological measurements of Tri-AT. The angular frequency for strain sweep (a) is 10 rad s^{-1} and the strain for the dynamic frequency sweep (b) is 1% (temperature: 25 ºC). (c) Storage modulus (*G'*), loss modulus (*G''*), and (d) complex viscosity (*η*) values at reversible temperature-dependent rheological tests.

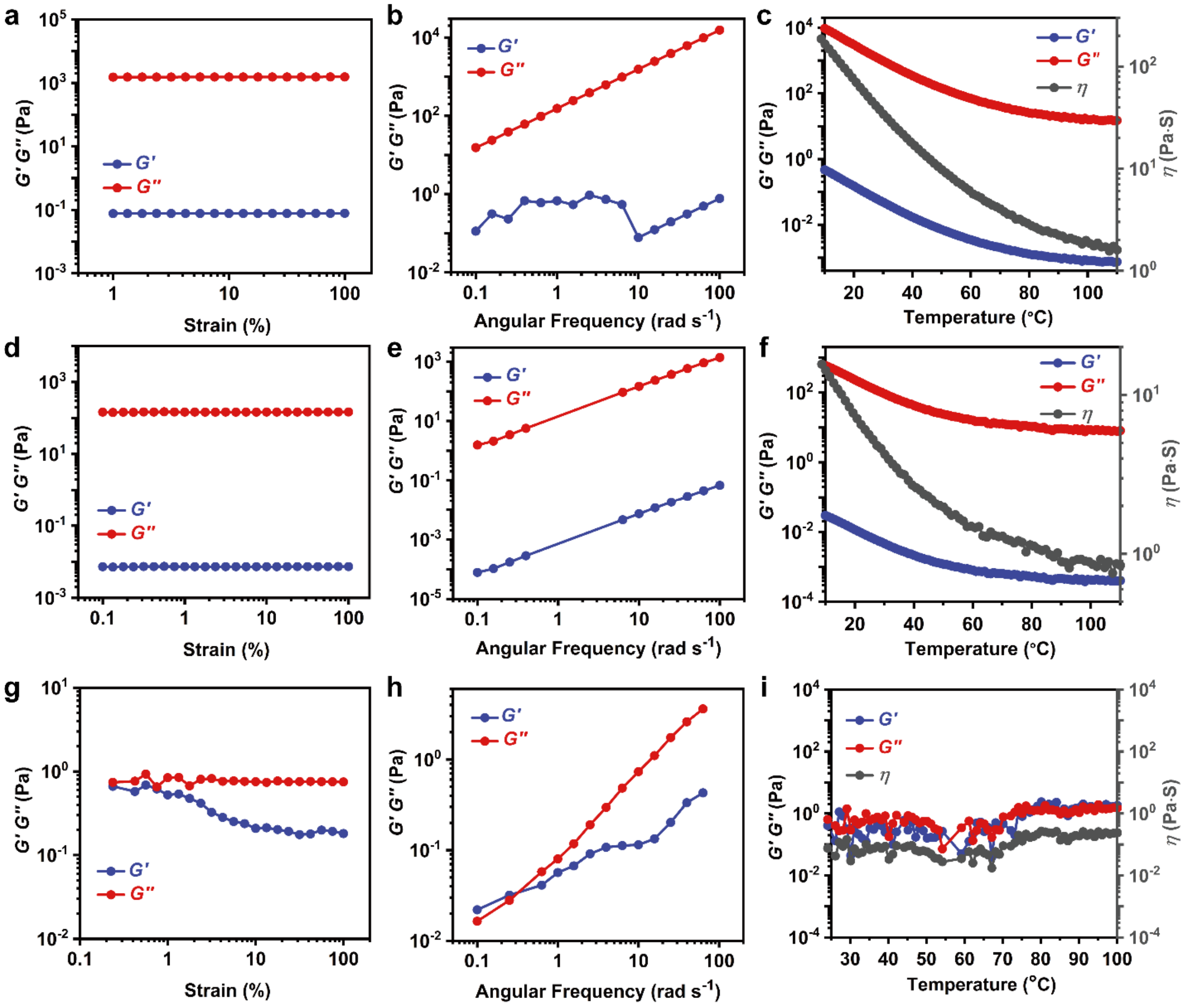

Supplementary Figure 40. Rheological measurements of other reference compounds. (a-c) Bis-HT, (d-f) Mon-HT and (g-i) BMImTFSI. The angular frequency for strain sweep is 10 rad s^{-1} and the strain for the dynamic frequency sweep is 1% (temperature: 25 ºC). (c), (f), and (i) Storage modulus (*G'*), loss modulus (*G''*), and complex viscosity (η) values at temperature-dependent rheological tests.

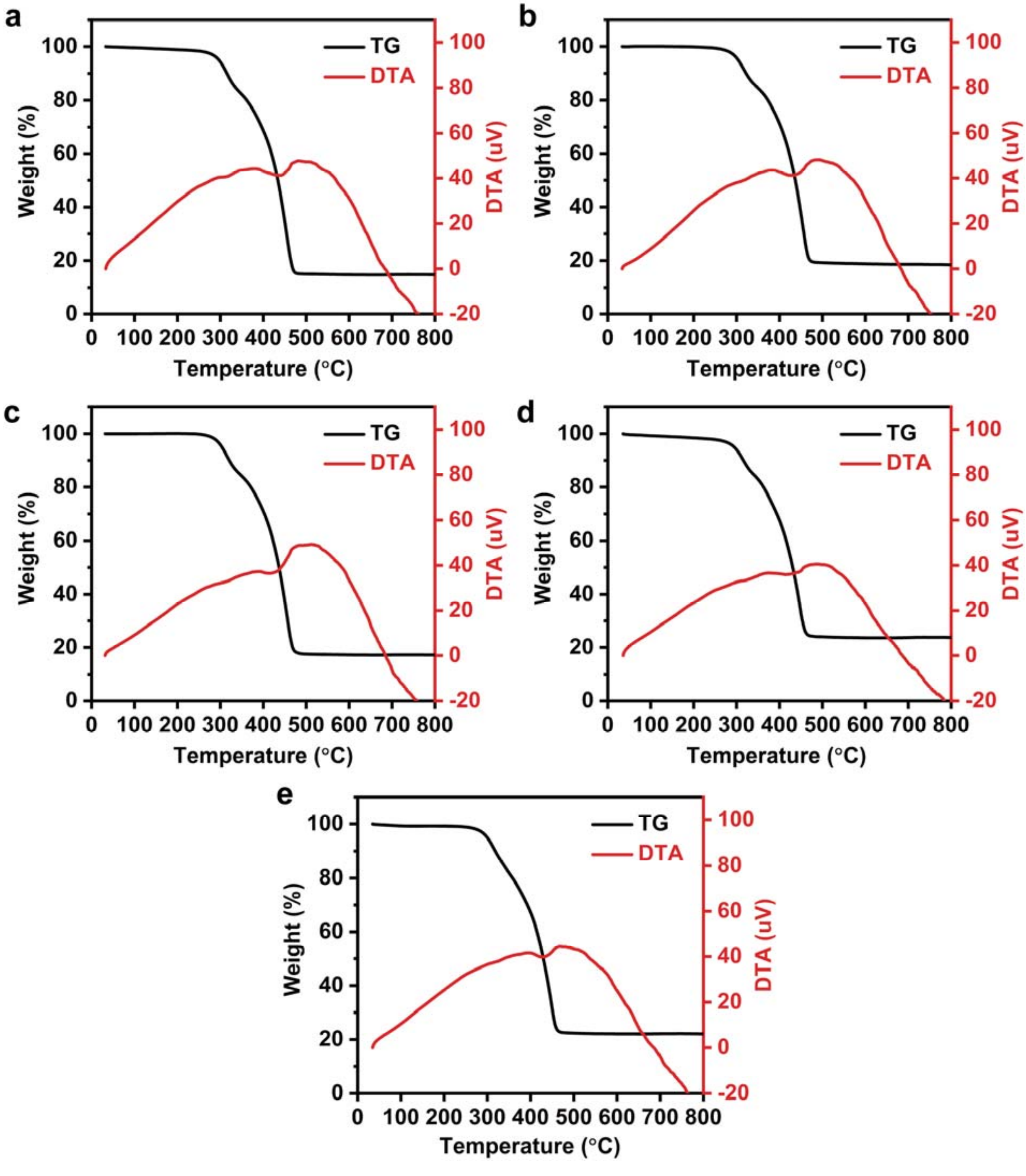

Supplementary Figure 41. TGA curves of Tri-HT/MWCNTs with different MWCNTs contents. (a) 6 wt%, (b) 7 wt%, (c) 8 wt%, (d) 9 wt%, (e) 10 wt%.

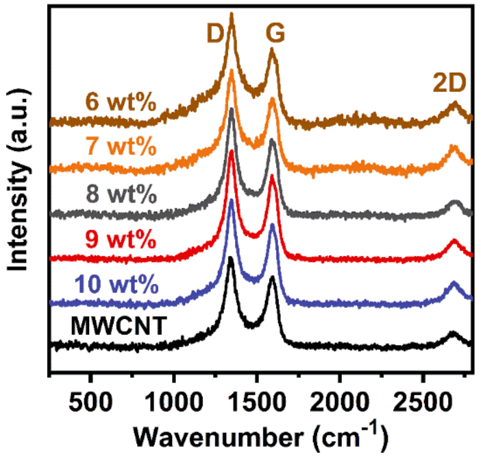

Supplementary Figure 42. Raman spectra of Tri-HT/MWCNTs with different MWCNTs contents. Well preserved characteristic peaks (D, G, and 2D) of MWCNTs indicated that the simple preparation process of Tri-HT/MWCNTs has little influence on MWCNTs. Thus, the well electronic conductivity of MWCNTs in the composite can be ensured.

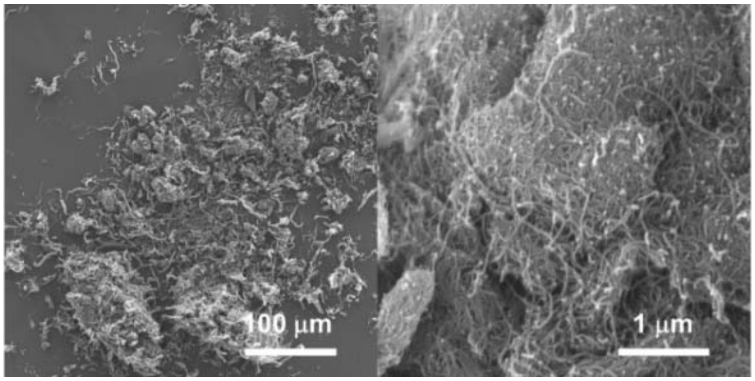

Supplementary Figure 43. SEM images of pure MWCNTs at different magnification scales.

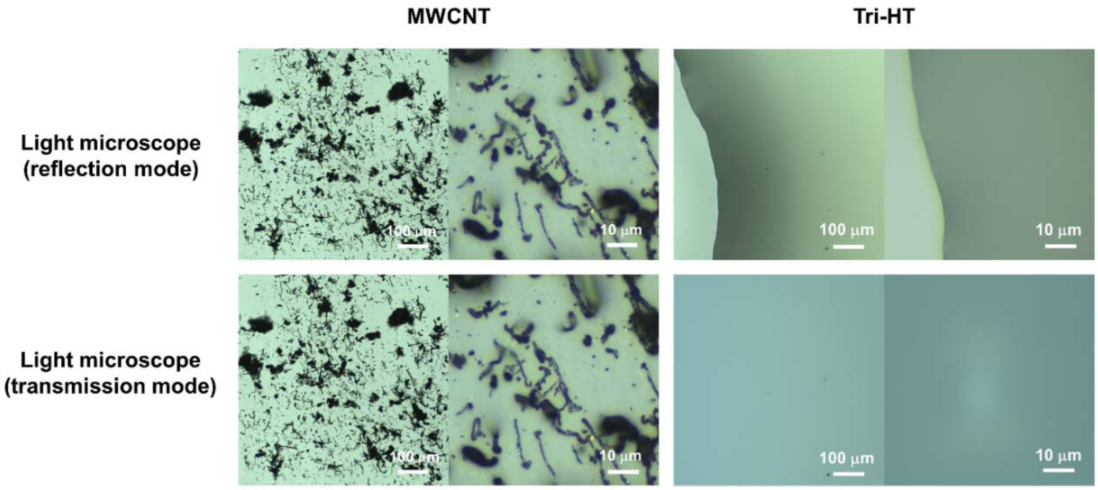

Supplementary Figure 44. LM (reflection and transmission modes) images of MWCNTs and Tri-HT at different magnification scales. For clarity, the boundary of Tri-HT was contrasted, showing it was transmittable under visible light.

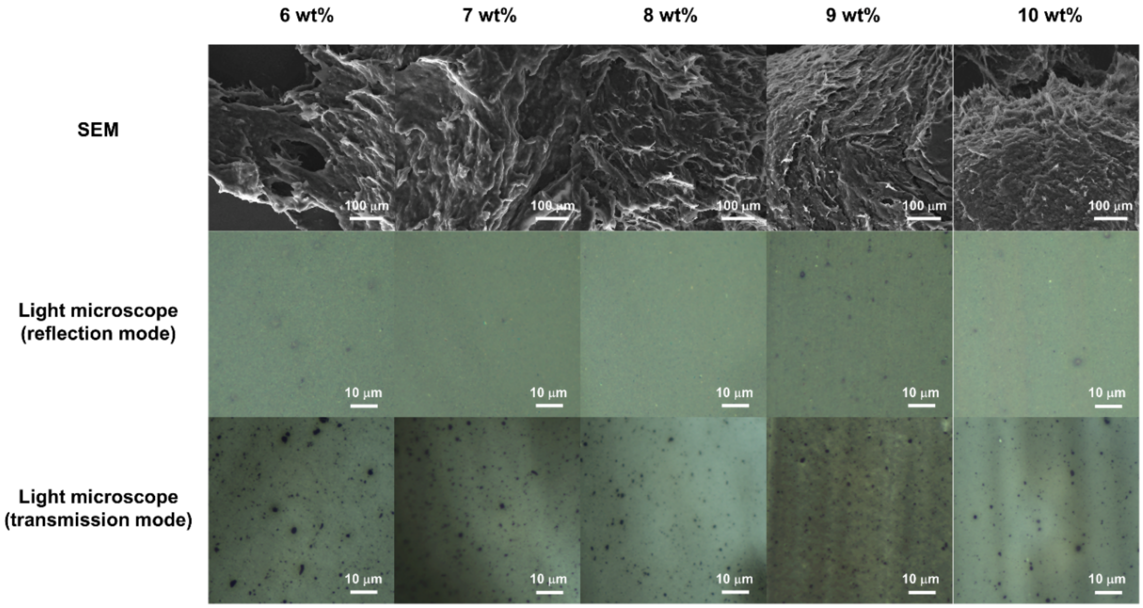

Supplementary Figure 45. SEM and LM (reflection and transmission modes) images of Tri-HT/MWCNTs with variable MWCNTs contents. MWCNTs were shown as black dots in LM images.

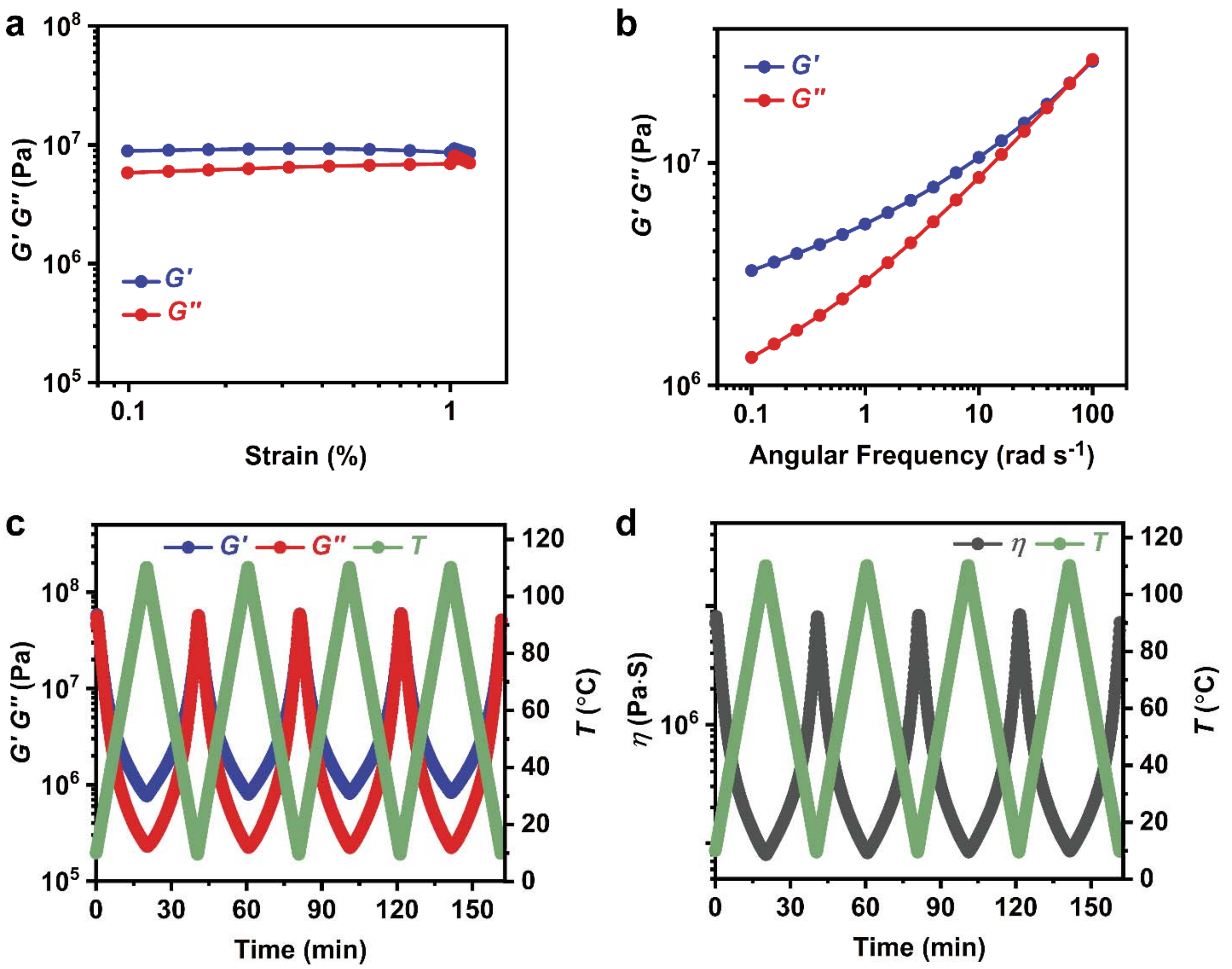

Supplementary Figure 46. Rheological measurements of Tri-HT/MWCNTs (6 wt%). The angular frequency for strain sweep (a) is 10 rad s^{-1} and the strain for the dynamic frequency sweep (b) is 1% (temperature: 25 ºC). (c) Storage modulus (*G'*), loss modulus (*G''*), and (d) complex viscosity (*η*) values at reversible temperature-dependent rheological tests.

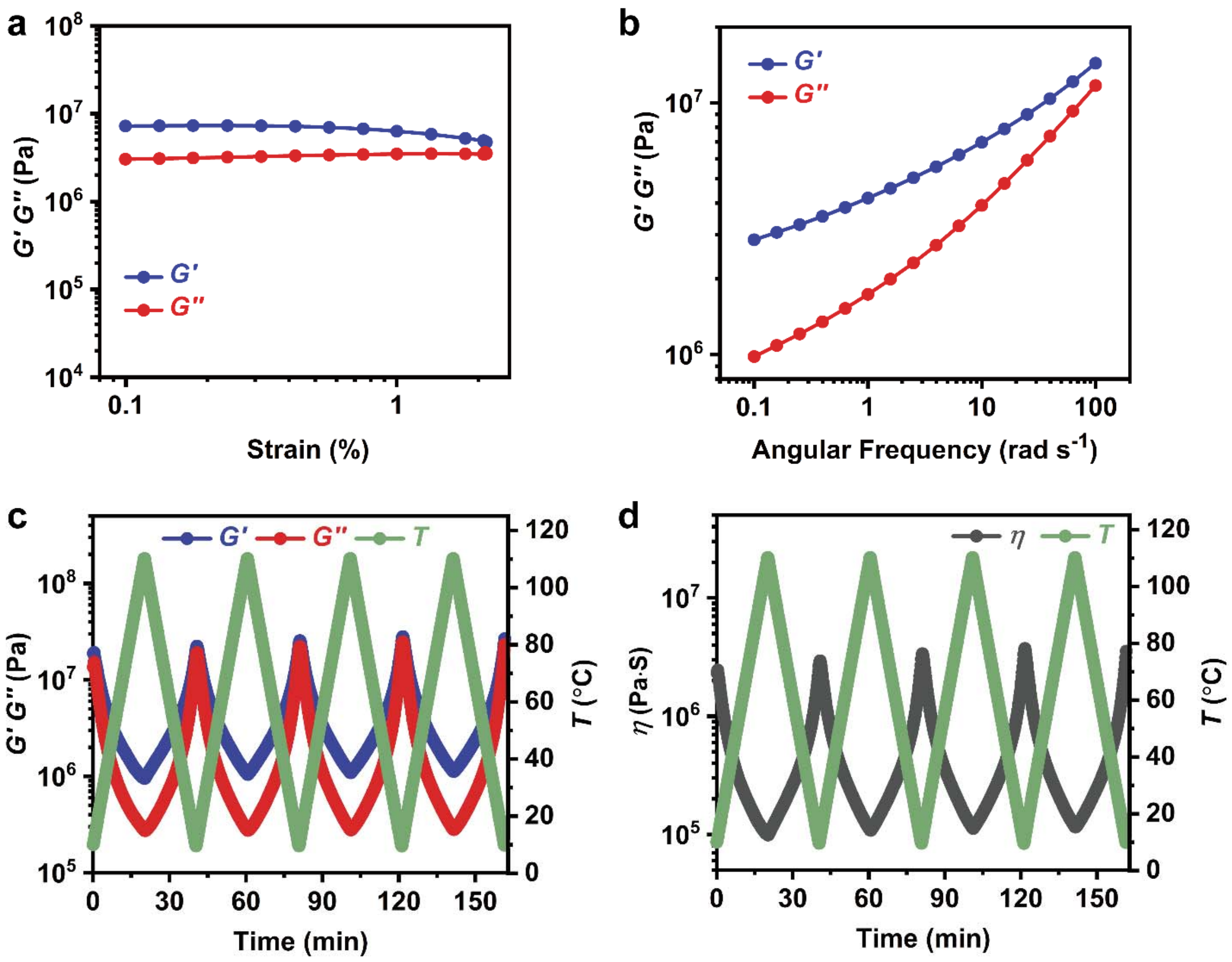

Supplementary Figure 47. Rheological measurements of Tri-HT/MWCNTs (7 wt%). The angular frequency for strain sweep (a) is 10 rad s^{-1} and the strain for the dynamic frequency sweep (b) is 1% (temperature: 25 ºC). (c) Storage modulus (*G'*), loss modulus (*G''*), and (d) complex viscosity (*η*) values at reversible temperature-dependent rheological tests.

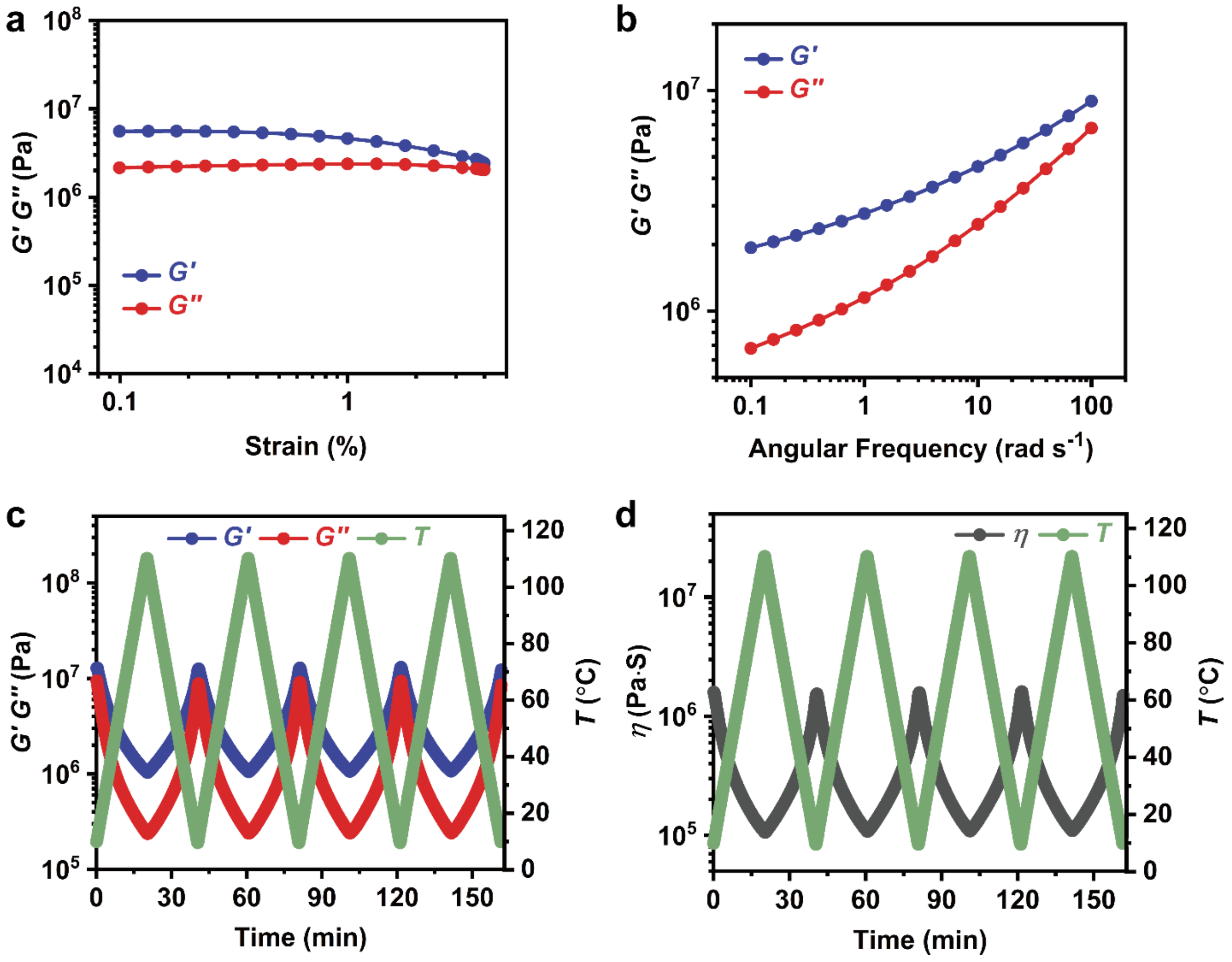

Supplementary Figure 48. Rheological measurements of Tri-HT/MWCNTs (8 wt%). The angular frequency for strain sweep (a) is 10 rad s^{-1} and the strain for the dynamic frequency sweep (b) is 1% (temperature: 25 ºC). (c) Storage modulus (*G’*), loss modulus (*G’’*), and (d) complex viscosity (η) values at reversible temperature-dependent rheological tests.

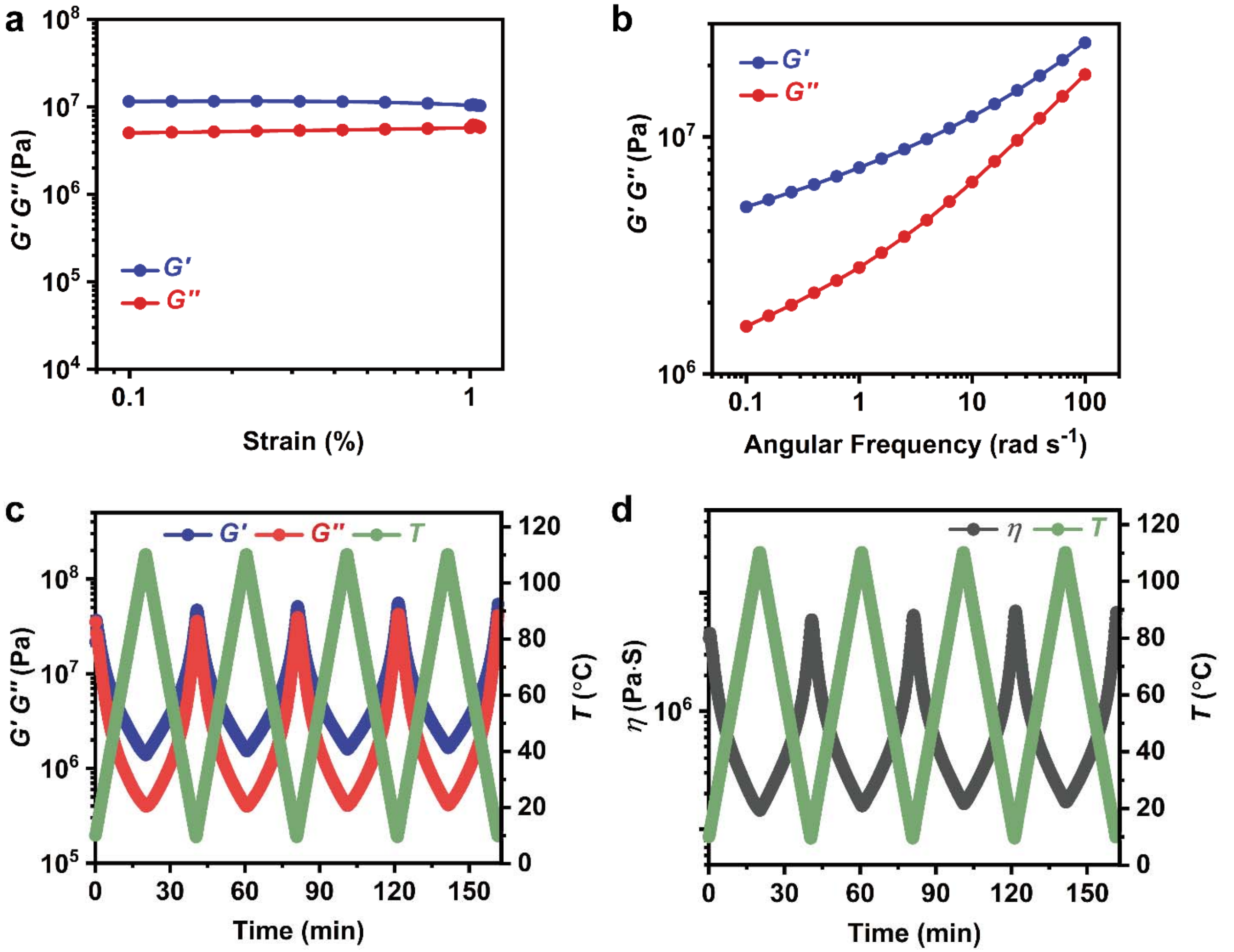

Supplementary Figure 49. Rheological measurements of Tri-HT/MWCNTs (9 wt%). The angular frequency for strain sweep (a) is 10 rad s^{-1} and the strain for the dynamic frequency sweep (b) is 1% (temperature: 25 ºC). (c) Storage modulus (G'), loss modulus (G''), and (d) complex viscosity (η) values at reversible temperature-dependent rheological tests.

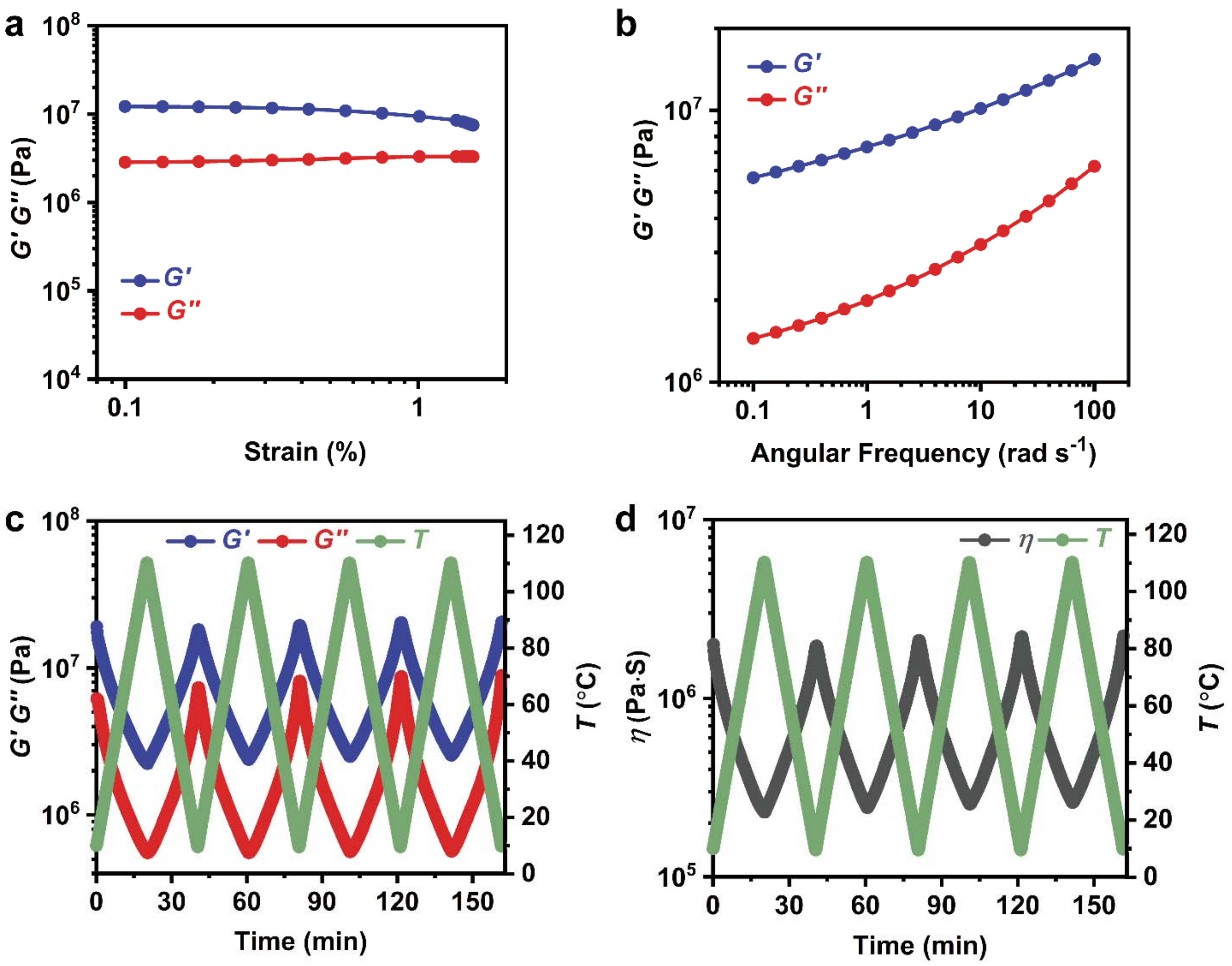

Supplementary Figure 50. Rheological measurements of Tri-HT/MWCNTs (10 wt%). The angular frequency for strain sweep (a) is 10 rad s^{-1} and the strain for the dynamic frequency sweep (b) is 1% (temperature: 25 ºC). (c) Storage modulus (*G'*), loss modulus (*G''*), and (d) complex viscosity (η) values at reversible temperature-dependent rheological tests.

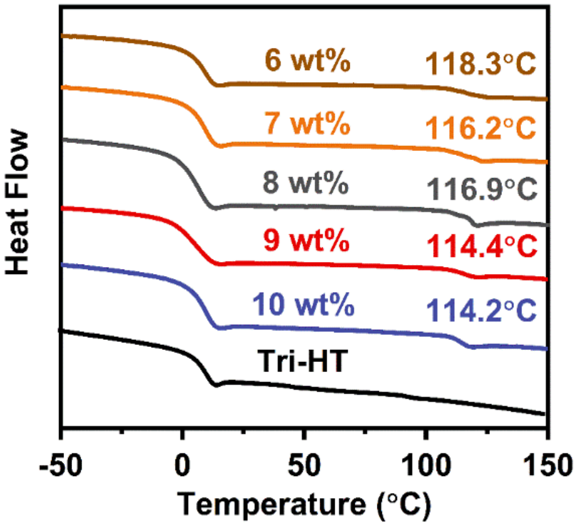

Supplementary Figure 51. DSC spectra of Tri-HT/MWCNTs with variable MWCNTs contents.

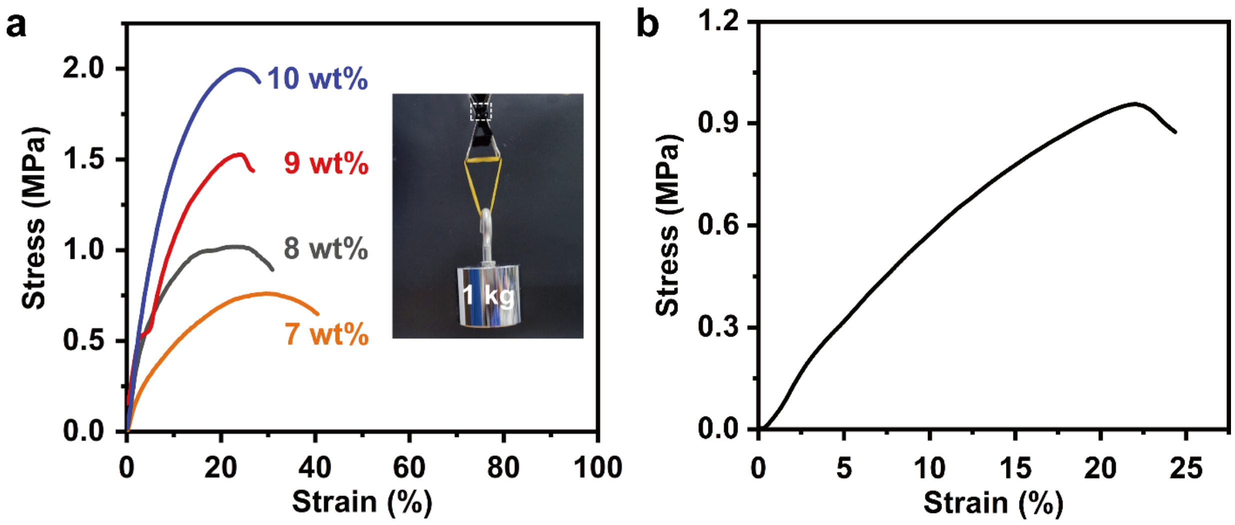

Supplementary Figure 52. Mechanical properties of Tri-HT/MWCNTs with variable MWCNTs contents. (a) Stress-strain curve of Tri-HT/MWCNTs with variable MWCNTs contents. The inset photograph illustrates the high mechanical strength of Tri-HT/MWCNTs (cross section area=0.5 cm^2). Note that Tri-HT itself and composite adhesives containing 6 wt% MWCNTs have weak mechanical strength and cannot be shaped for stress-strain test. (b) The Stress-strain curve of self-

healed (100 °C, 24 h) T Tri-HT/MWCNTs (10 wt%) (speed: 100 mm min^{-1}). All tests were carried out at 25 °C.

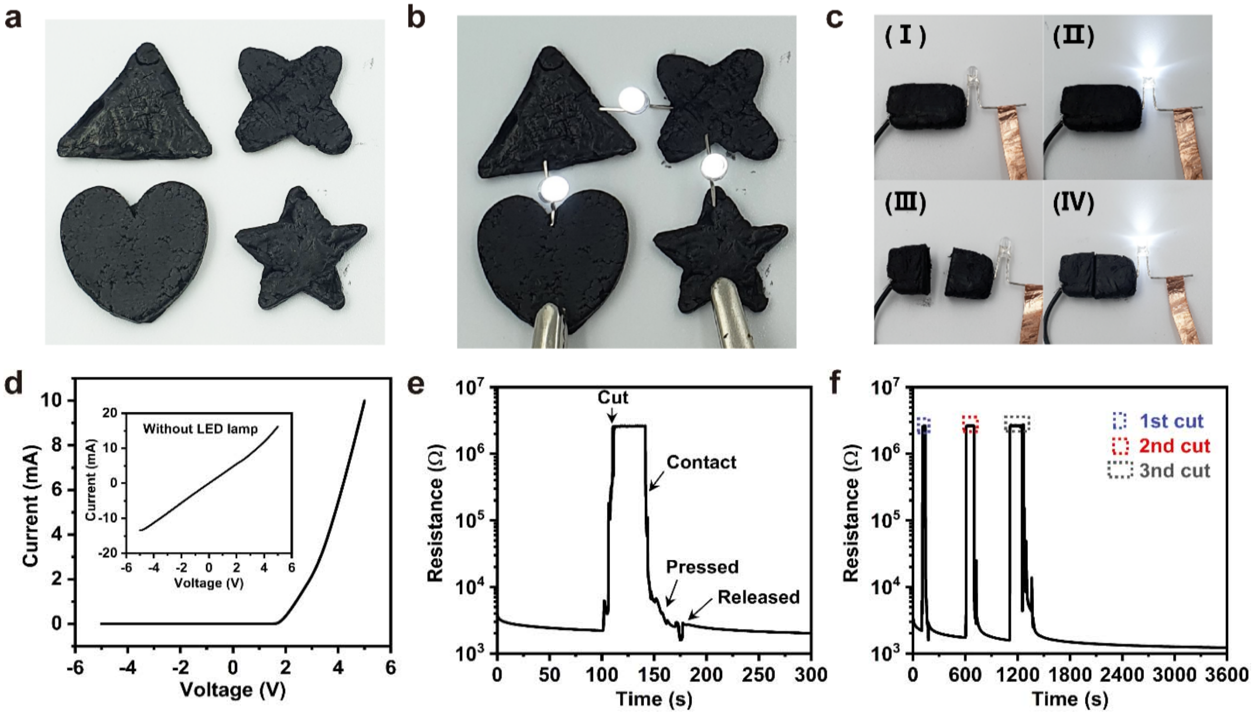

Supplementary Figure 53. Electrical healing characterization of Tri-HT/MWCNTs (10 wt%). (a) Molded Tri-HT/MWCNTs and (b) circulated using a commercial LED lamp (voltage: 3 V, temperature: 25 °C). (c) Illustration of the healing process for Tri-HT/MWCNTs with an LED. Initial state (I) before and (II) after circulated. (III) Cut off the electrical conductor (open circuit) and (IV) electrical healing in the contact process. (d) Current–voltage curve of Tri-HT/MWCNTs with a LED lamp. The inset shows the current–voltage curve of Tri-HT/MWCNTs without LED lamp, indicating the conductivity of Tri-HT/MWCNTs was constant in the tested voltage range. (e) Time evolution of the electrical healing process (healing time: 30 s) at room temperature. (f) Repeated electrical healing 3 times at the same location.

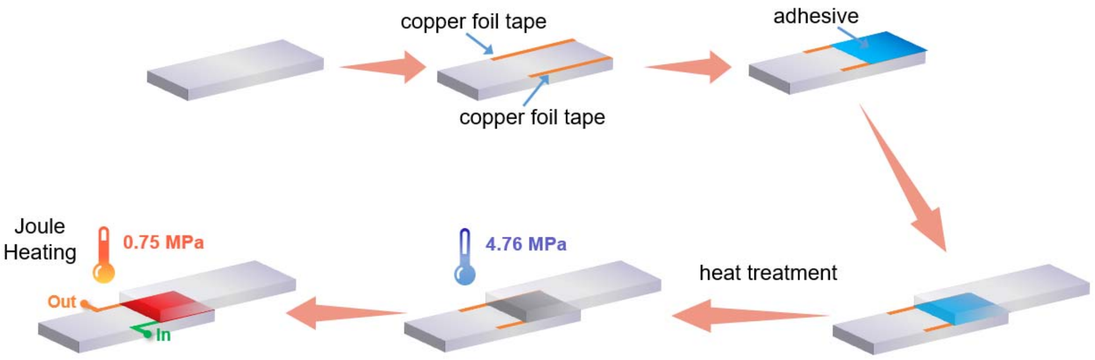

Supplementary Figure 54. Detailed procedure to measure the Joule-heating effect of Tri-HT/MWCNT adhesive.

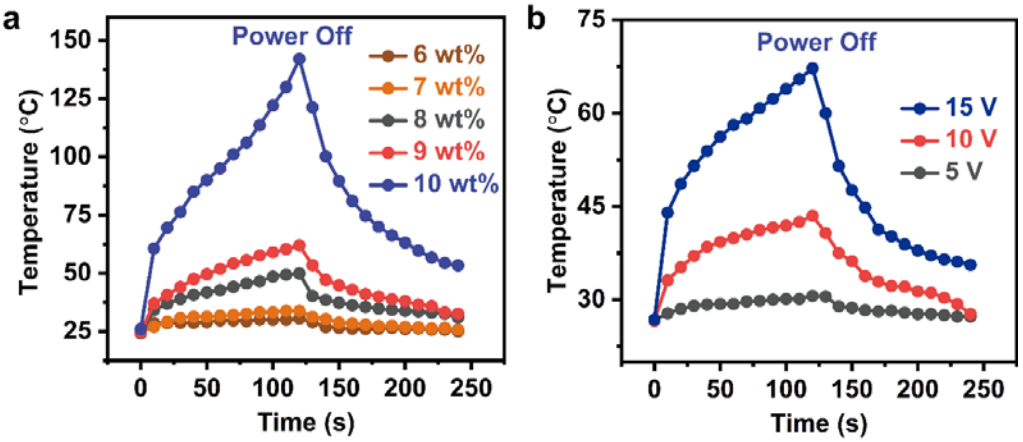

Supplementary Figure 55. The temperature evolving curves of Tri-HT/MWCNTs with variable MWCNTs contents. (a) Tri-HT/MWCNTs with variable MWCNTs contents under an applied voltage of 20 V and (b) Tri-HT/MWCNTs (10 wt%) under different voltages.

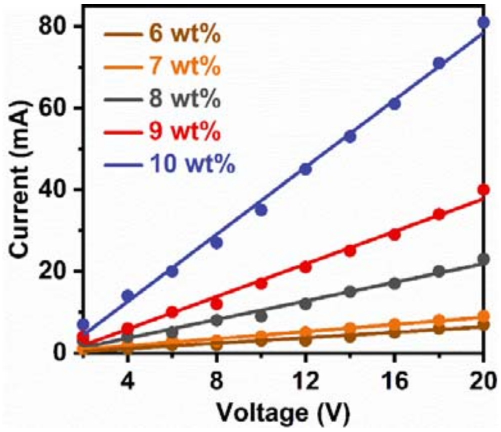

Supplementary Figure 56. The current-voltage curves of Tri-HT/MWCNTs with variable MWCNTs contents.

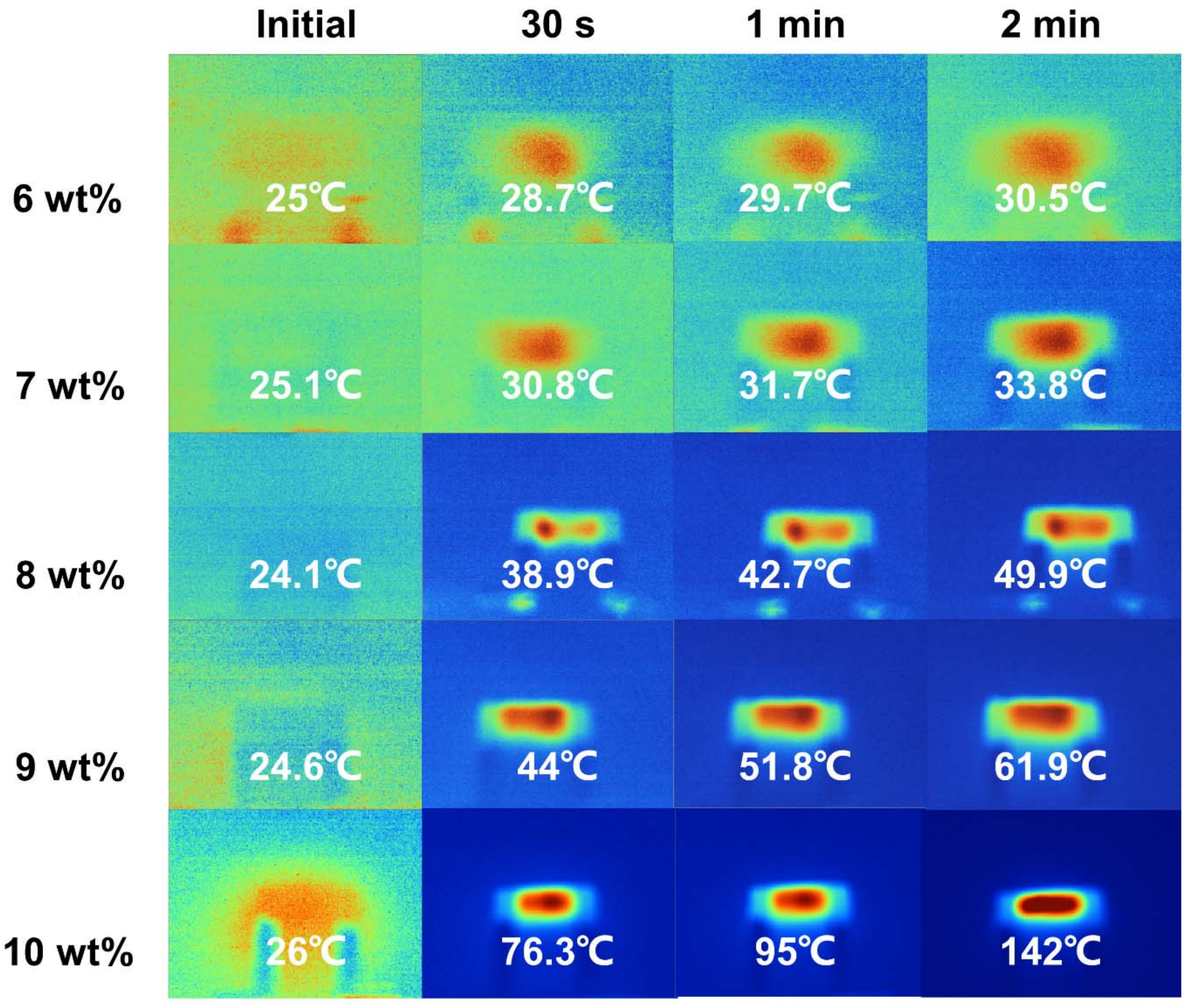

Supplementary Figure 57. The temperature detected by IR thermal imager for Tri-HT/MWCNTs with variable MWCNTs contents under an applied voltage of 20 V.

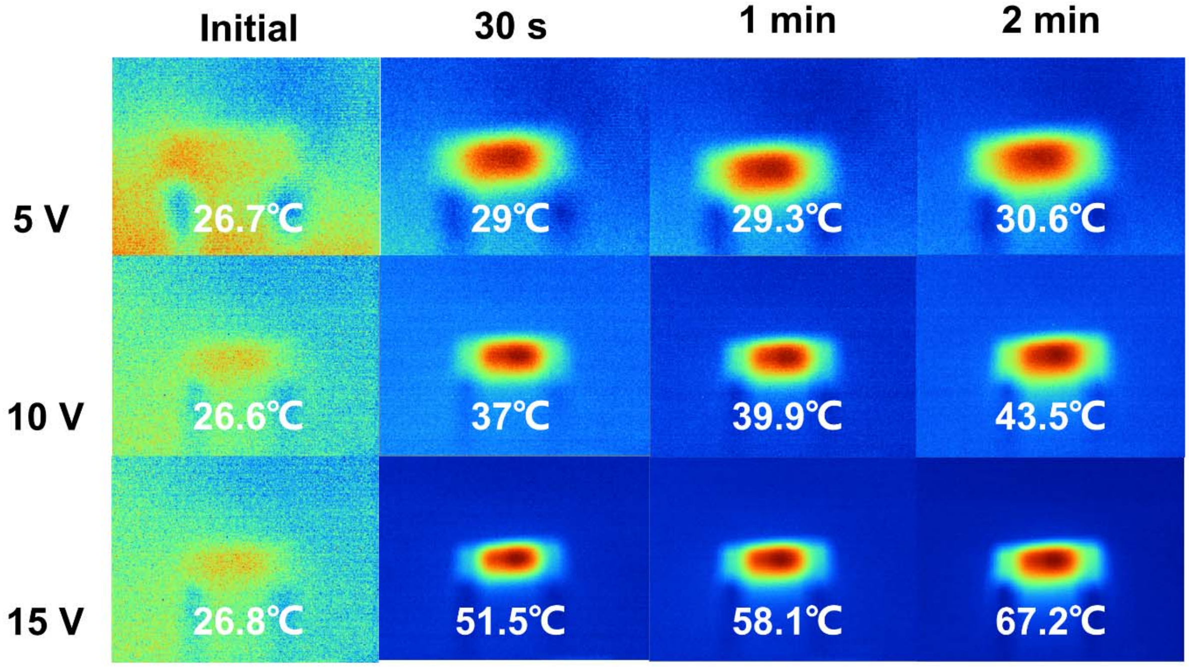

Supplementary Figure 58. The temperature detected by IR thermal imager for Tri-HT/MWCNTs (10 wt%) under different voltages.

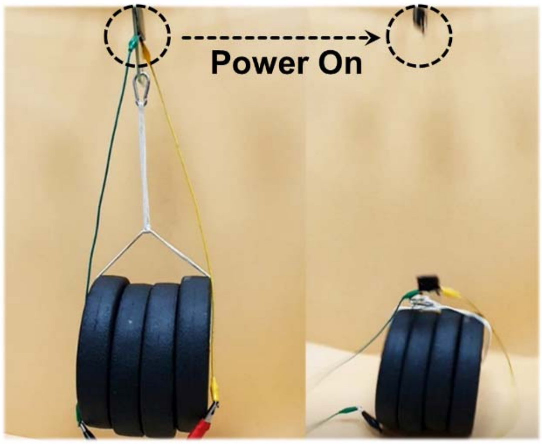

Supplementary Figure 59. Macroscopic adhesion test of Tri-HT/MWCNTs (10 wt%) on glass substrate. After power on 1 min, de-bonding occurs (6 kg weight).

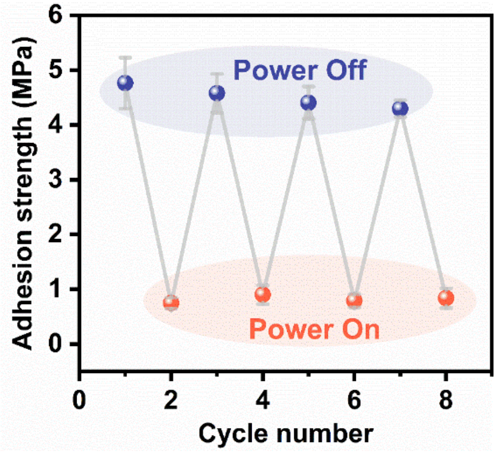

Supplementary Figure 60. The reversible adhesion strength changes of Tri-HT/MWCNTs (10 wt%) on glass substrate for 4 cycles. All tests were carried out at 25 °C. Error bars are standard deviation for n=3~5 measurements.

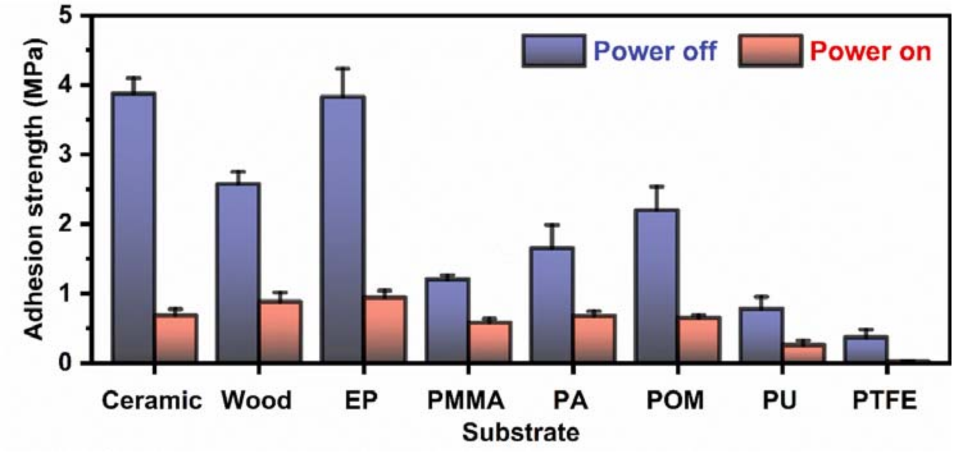

Supplementary Figure 61. Differences in adhesion strength of Tri-HT/MWCNTs (10 wt%) on different substrates. All tests were carried out at 25 °C. Error bars are standard deviation for n=3~5 measurements.

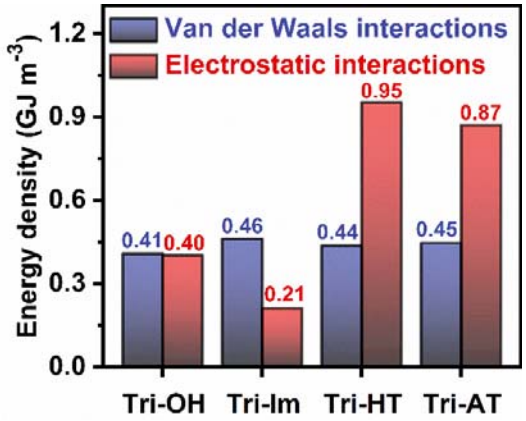

Supplementary Figure 62. The composition of theoretically calculated CED. Van der Waals force is the reflection of the attractive and the repulsive non-bond forces between molecules. Electrostatic interactions describe the attraction between polar or completely ionized groups, including ionic interactions, H-bonding interactions. Electrostatic interactions of ILs (e.g., Tri-HT and Tri-AT) were much higher than that of nonionic analogs (e.g., Tri-OH and Tri-Im). In addition, each model holds an additional uncertain energy density of ~0.02 GJ m^{-3}.

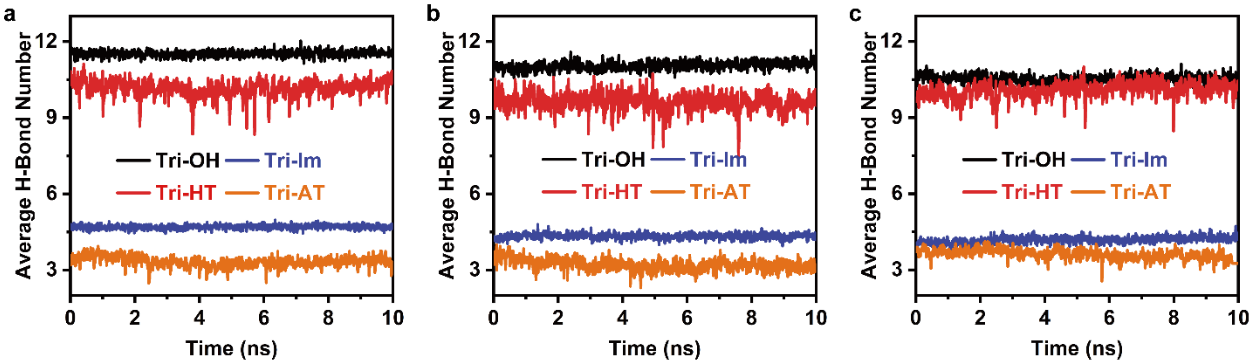

Supplementary Figure 63. Average H-bond number of Tri-OH, Tri-Im, Tri-HT and Tri-AT at different temperatures. (a) -100 ºC, (b) 25 ºC and (c) 100 ºC.

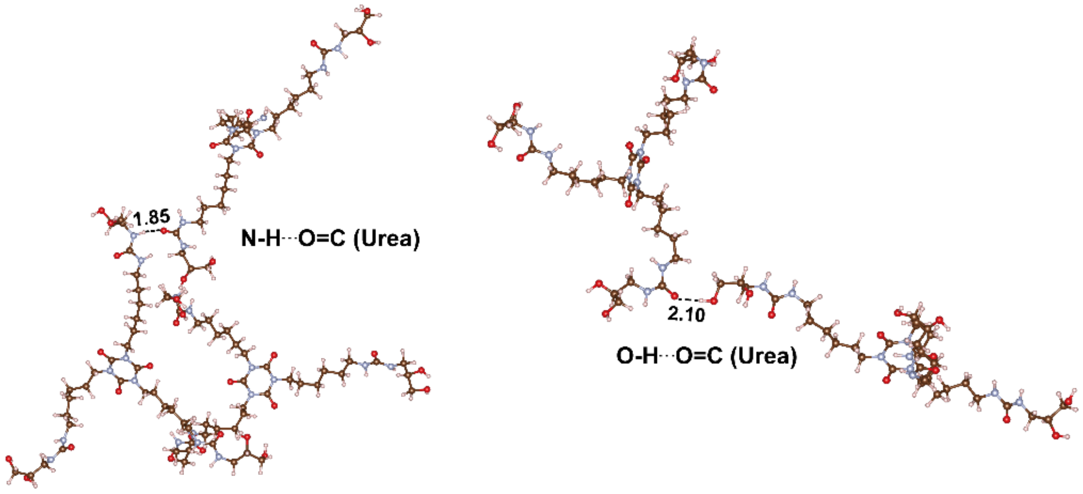

Supplementary Figure 64. The most stable conformation with minimum energy of Tri-OH. H-bonding interactions are presented by dotted lines. The unit of interatomic distance is Å.

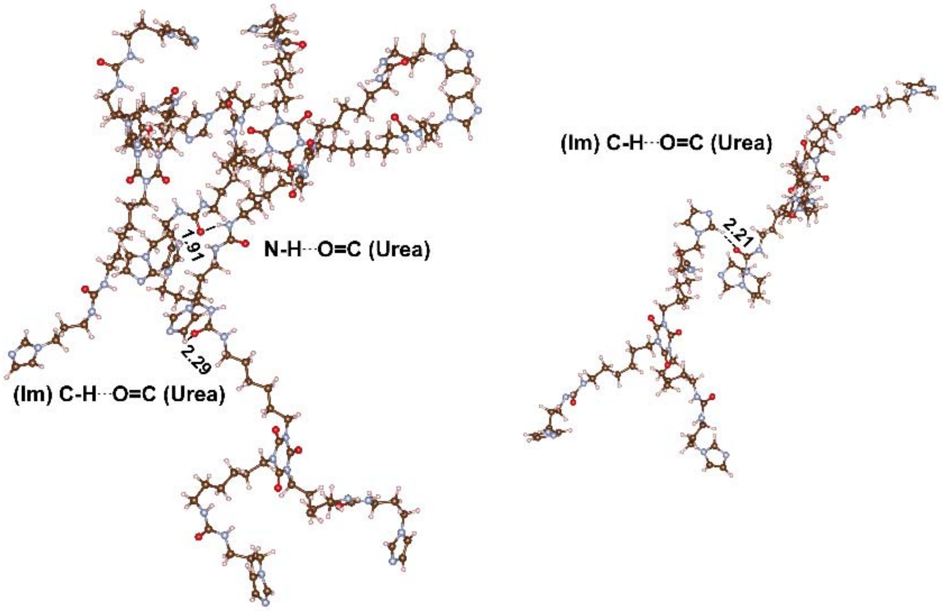

Supplementary Figure 65. The most stable conformation with minimum energy of Tri-Im. H-bonding interactions are presented by dotted lines. The unit of interatomic distance is Å.

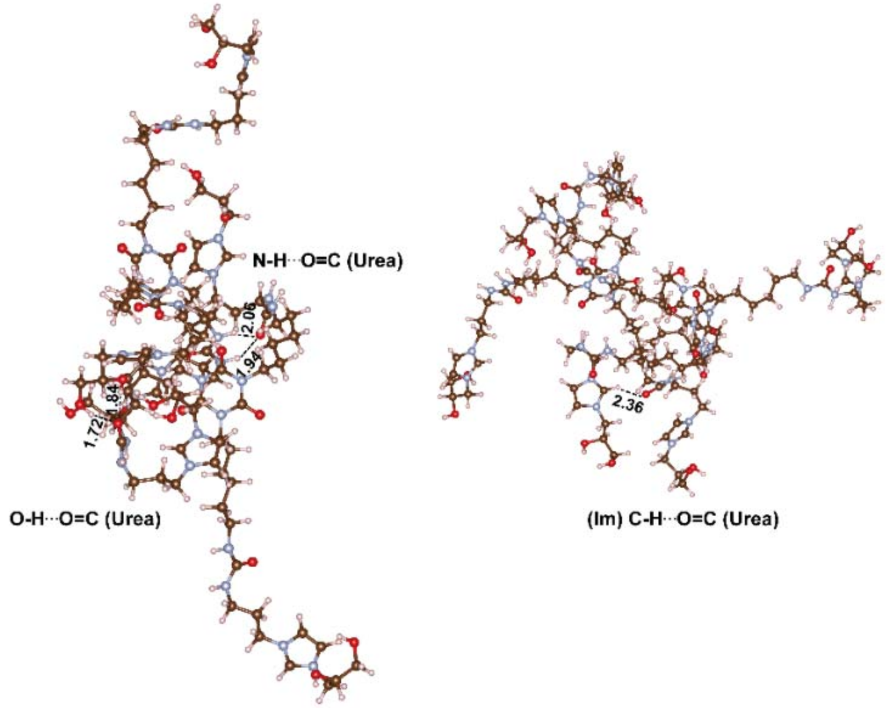

Supplementary Figure 66. The most stable conformation with minimum energy of Tri-HT. H-bonding interactions are presented by dotted lines. The unit of interatomic distance is Å.

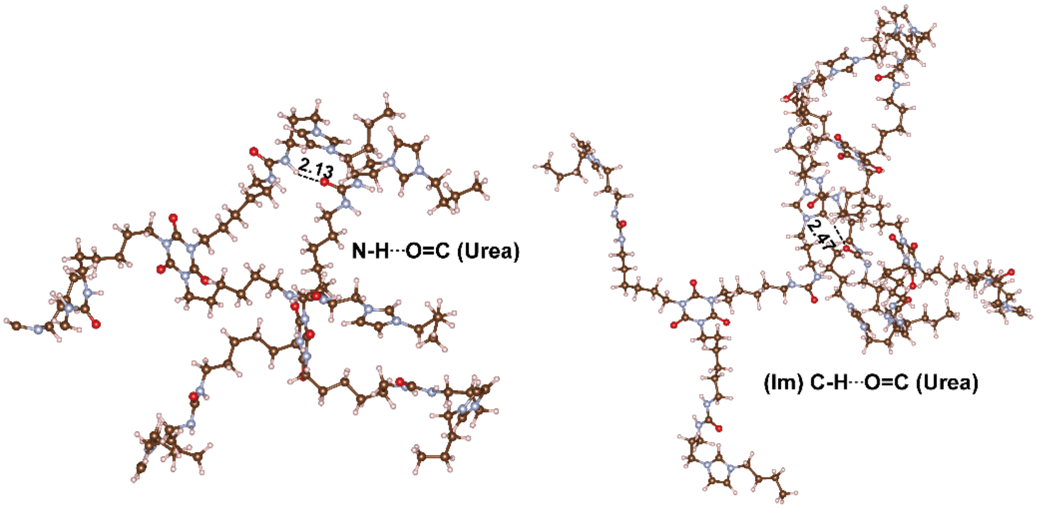

Supplementary Figure 67. The most stable conformation with minimum energy of Tri-AT. H-bonding interactions are presented by dotted lines. The unit of interatomic distance is Å.

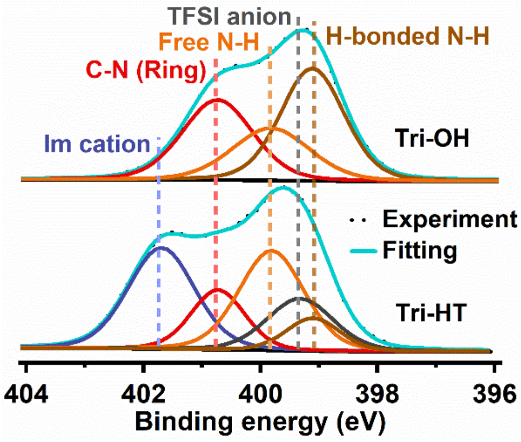

Supplementary Figure 68. High-resolution N1s XPS spectra of Tri-HT and nonionic Tri-OH.

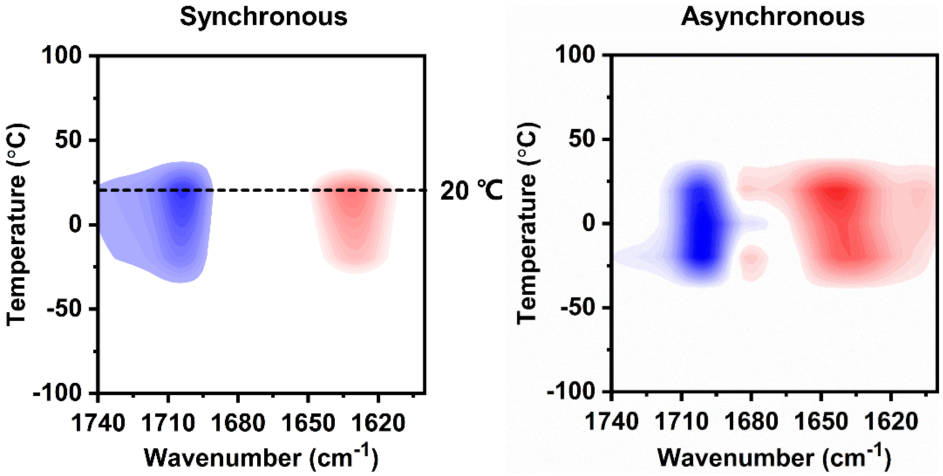

Supplementary Figure 69. PCMW synchronous and asynchronous spectra of Tri-HT. In the contour maps, red colors are defined as positive intensities, and blue colors are defined as negatives.

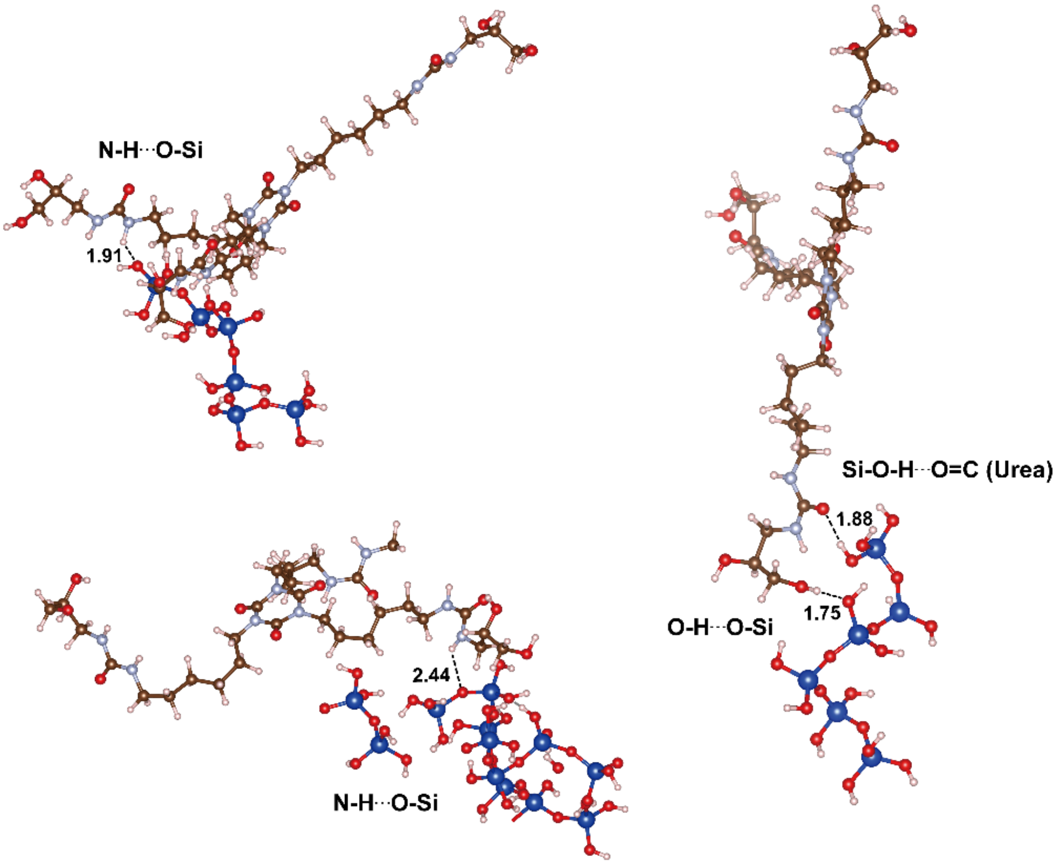

Supplementary Figure 70. The most stable conformation with minimum energy of Tri-OH on polyhydric substrate surface. H-bonding interactions are presented by dotted lines. The unit of interatomic distance is Å.

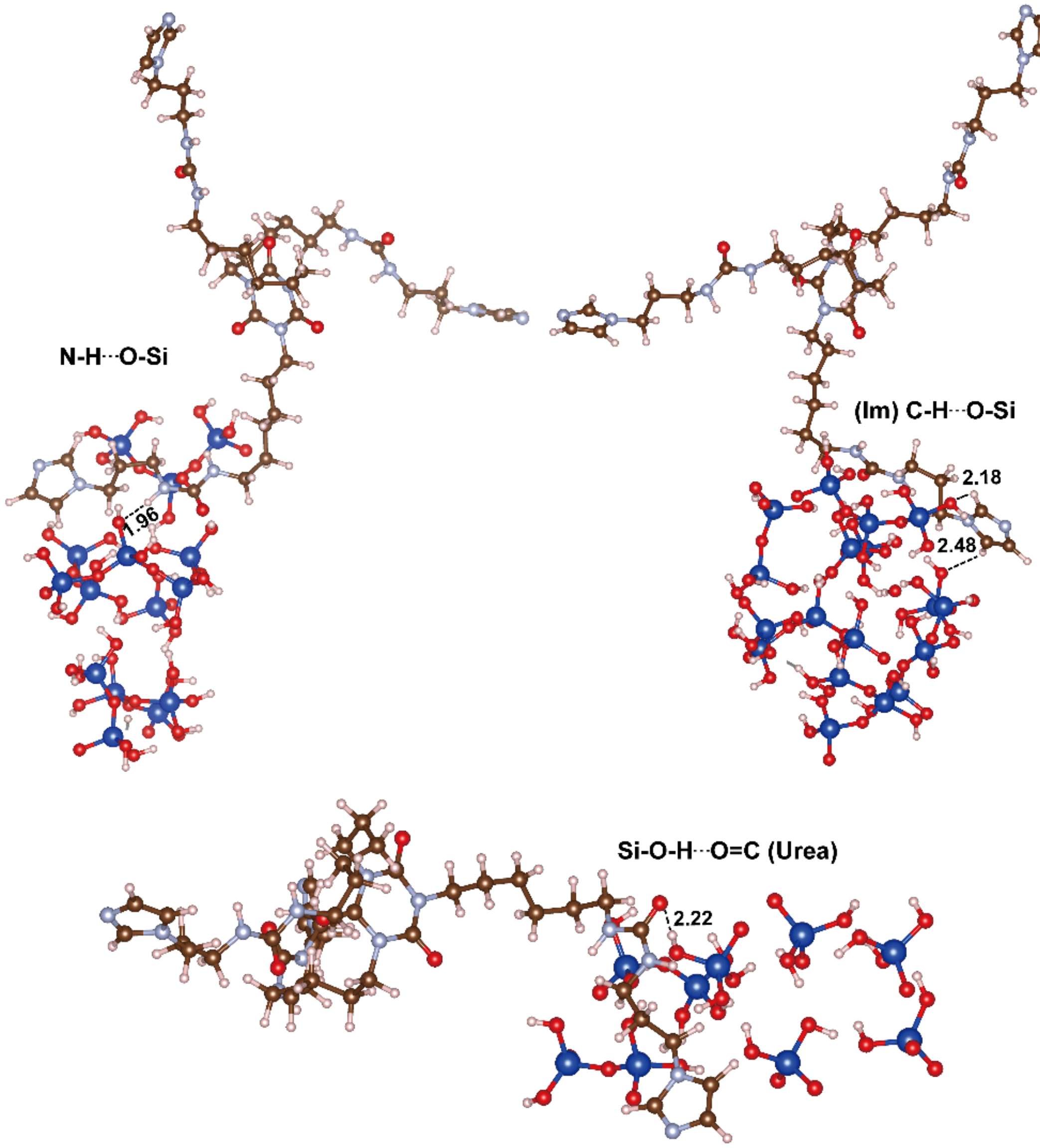

Supplementary Figure 71. The most stable conformation with minimum energy of Tri-Im on polyhydric substrate surface. H-bonding interactions are presented by dotted lines. The unit of interatomic distance is Å.

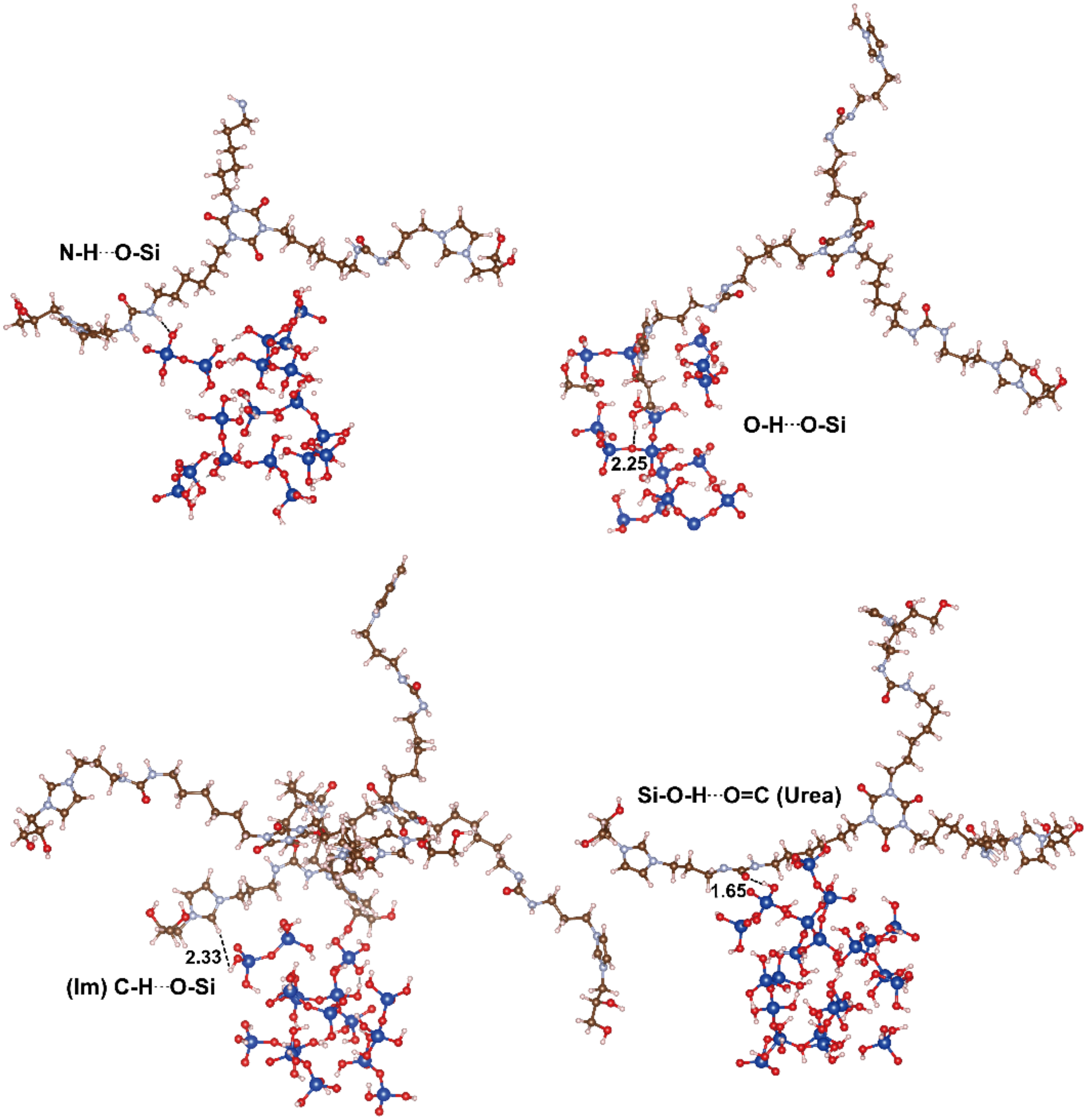

Supplementary Figure 72. The most stable conformation with minimum energy of Tri-HT on polyhydric substrate surface. H-bonding interactions are presented by dotted lines. The unit of interatomic distance is Å.

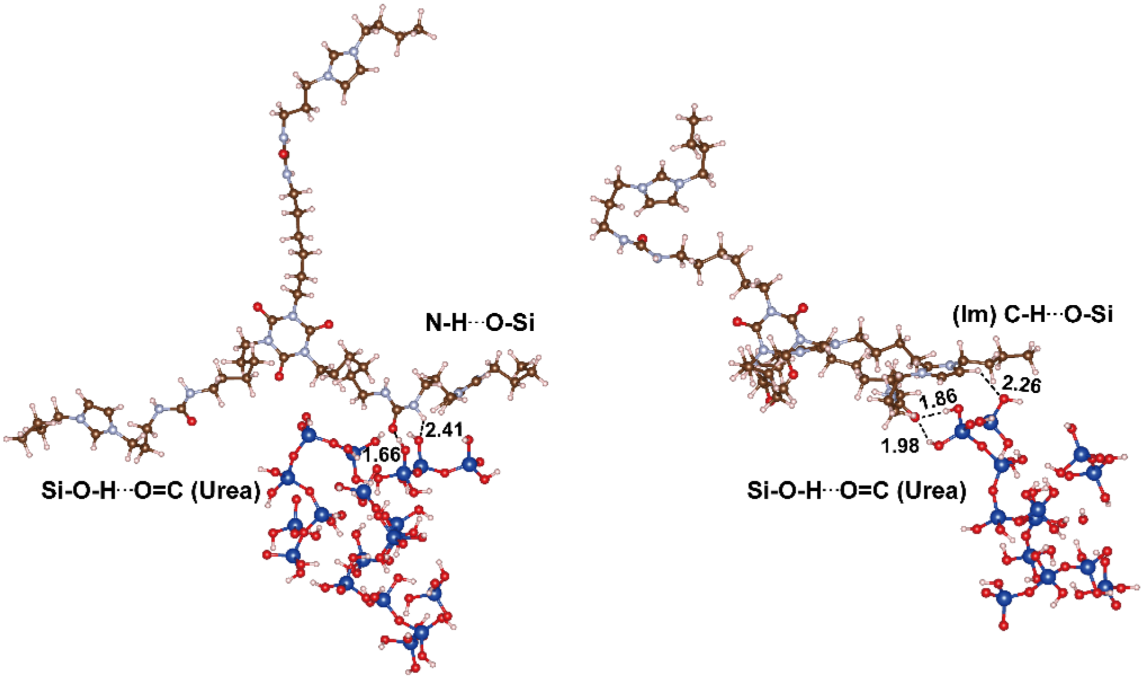

Supplementary Figure 73. The most stable conformation with minimum energy of Tri-AT on polyhydric substrate surface. H-bonding interactions are presented by dotted lines. The unit of interatomic distance is Å.

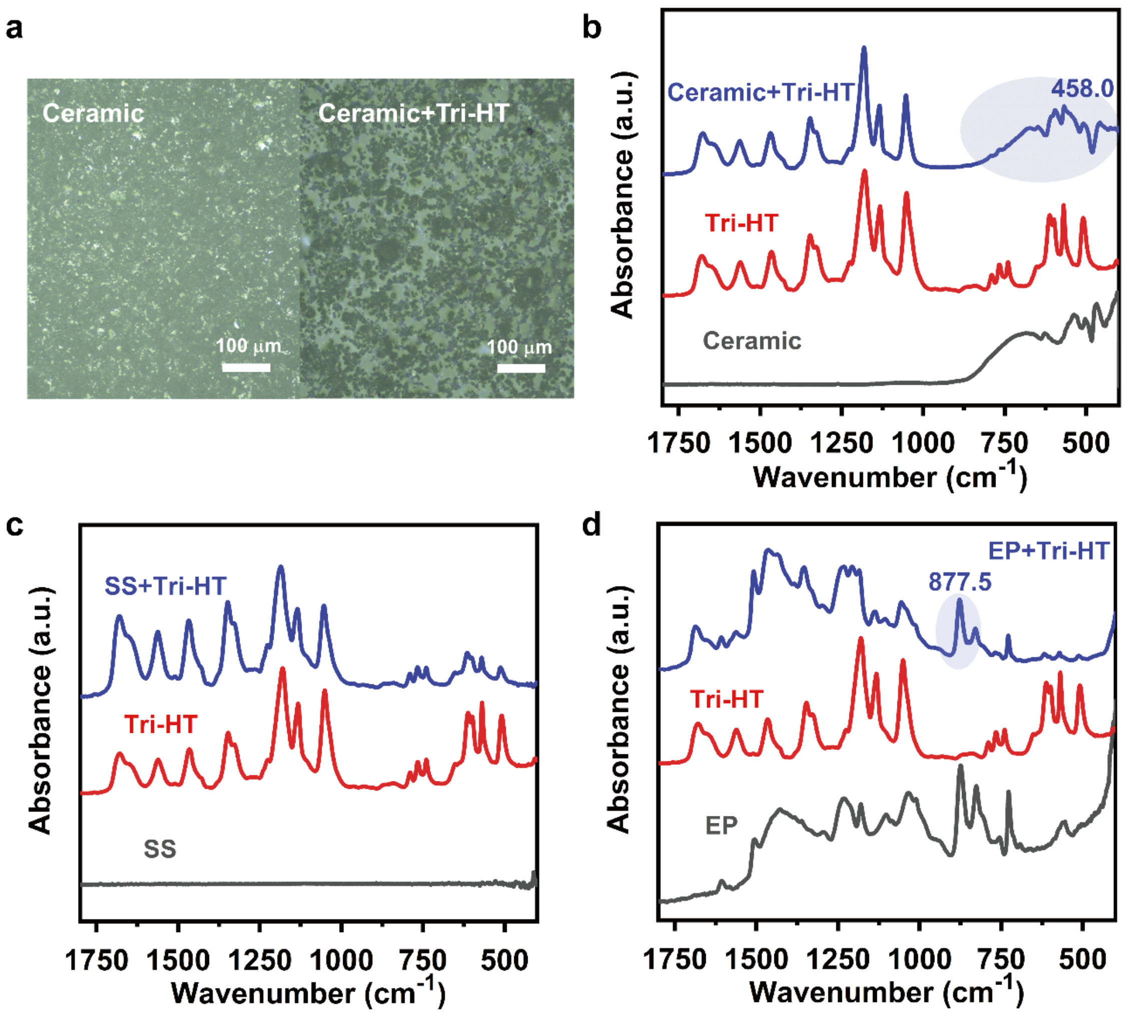

Supplementary Figure 74. Comparison of the substrate surface before and after being coated with Tri-HT. (a) Light microscope (reflection mode) images of the ceramic substrate surface before and after being coated with Tri-HT by spray. (b-d) ATR-IR spectra of substrate surface, Tri-HT, and substrate surface coated with Tri-HT. The substrate peak at 458.0 and 877.5 confirmed the thickness of Tri-HT layer was lower than the depth of detection.

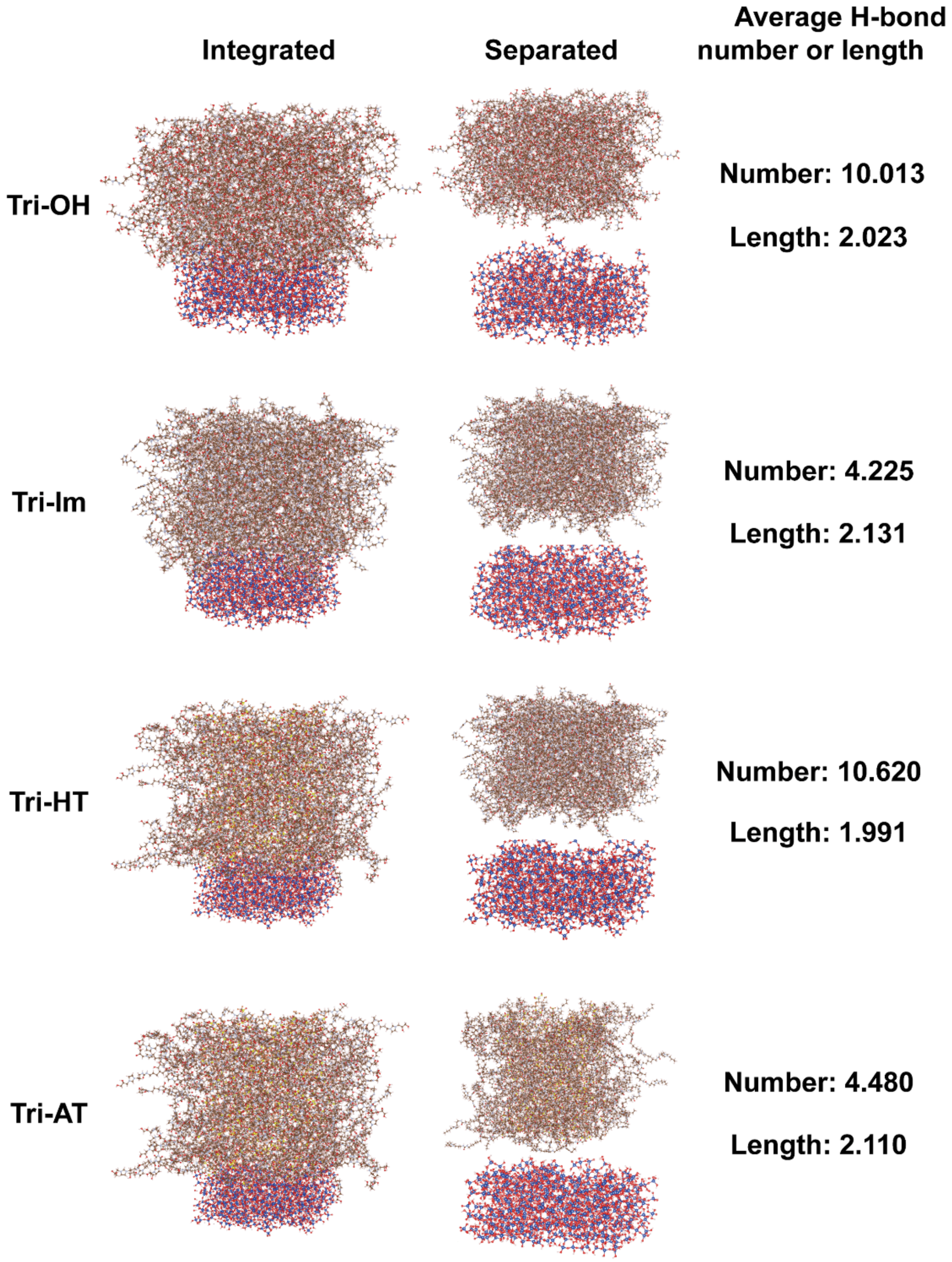

Supplementary Figure 75. The most stable conformations of molecular models: Tri-OH, Tri-Im, Tri-HT and Tri-AT on polyhydric substrate surface. Separated the adhesive models from the integrated models, the average H-bond number or length of the new re-calculated models was calculated. Compared with the molecular models without substrate, the differences of H-bonding interactions (including average H-bond number and length) were used to investigate the influences

of the substrate. To unveil the influence of the presence of the additional substrate on internal H-bonding interactions, the optimized theoretical models were further deliberately separated into two parts.

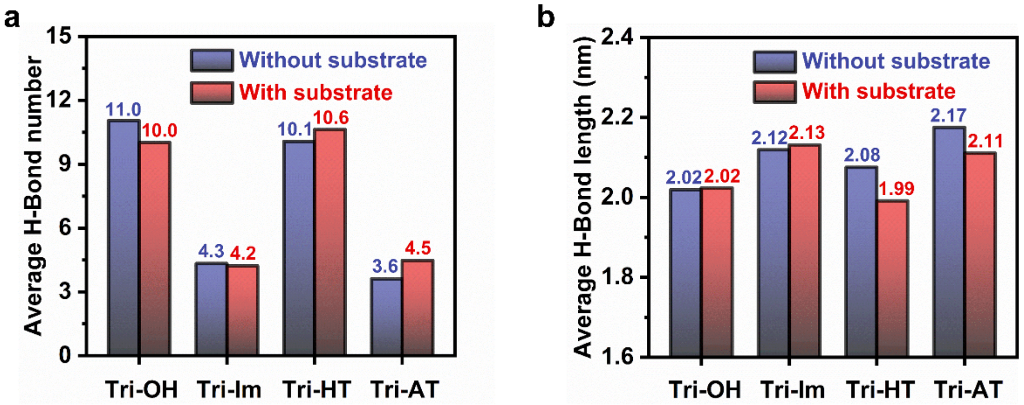

Supplementary Figure 76. Comparison of H-bonding interactions on the condition of without or with polyhydric substrate surface. (a) Average H-bond number and (b) average H-bond length of Tri-HT layer calculated from the most stable theoretical models on the condition of without or with polyhydric substrate surface.

Supplementary Table 1. Comparison of adhesion strength of Tri-HT and other reported low molecular-weight adhesives.

Adhesive	Molecular weight (g mol^{-1})	Adhesion strength (MPa)	Reference
His/SiW	3534.82	0.49	*Angew. Chem. Int. Ed.* 2017, 56, 8731
P1	857.06	1.34	*J. Am. Chem. Soc.* 2019, 141, 7385
LC	2415.63	1.60	*Nat. Commun.* 2016, 7, 12094
AESOIPA	1744.22	2.40	*ACS Appl. Poly. Mater.* 2019, 1, 1399
$(A)_n$	3541.85	4.20	*J. Am. Chem. Soc.* 2020, 142, 2579
Molecular solids	152.24	2.12	*Chem. Mater.* 2020, 32, 9882
P1	2034.17	4.17	*J. Am. Chem. Soc.* 2020, 142, 5371
DESPs	2475.88	6.57	*Angew. Chem. Int. Ed.* 2020, 59, 11871
Tri-HT	1945.78	10.50~12.20	This work

Supplementary Table 2. Temperature-dependent storage modulus (*G'*) and loss modulus (*G''*) of Tri-HT and reported supramolecular network polymers.

Supramolecular network polymers	Temperature (°C)	*G'* or *G"*	Reference
SHR	20 to 160	*G'* 20 MPa to 10 KPa<br>*G"* 20 MPa to 1 KPa	*Macromolecules* 2013, 46, 1841
Tr-PIB	-20 to 120	*G'* 3 MPa to 100 KPa<br>*G"* 4 MPa to 2 KPa	*Macromolecules* 2014, 47, 2122
polyurethane	0 to 120	*G'* 3 MPa to 40 Pa<br>*G"* 0.5 MPa to 1 KPa	*Chem. Sci.* 2016, 7, 4291
poly(TA-DIB-Fe)	25 to 120	*G'* 0.2 MPa to 7 KPa<br>*G"* 0.1 MPa to 7 KPa	*Sci. Adv.* 2018, 4, eaat8192
PDMS-Cat1-Zn	20 to 120	*G'* 10 MPa to 2 KPa<br>*G"* 1 MPa to 10 KPa	*ACS Appl. Mater. Interfaces* 2019, 11, 47382
DESPs	20 to 80	*G'* 10 MPa to 5 KPa<br>*G"* 10 MPa to 3 KPa	*Angew. Chem. Int. Ed.* 2020, 59, 11871
poly(TA)	20 to 100	*G'* 1 MPa to 100 Pa<br>*G"* 1 MPa to 500 Pa	*ACS Appl. Mater. Interfaces* 2021, 13, 44860
IC-1	25 to 100	*G'* 1.6 MPa to 500 Pa<br>*G"* 1 MPa to 400 Pa	*Angew. Chem. Int. Ed.* 2021, 133, 9030

SEA-0.2	20 to 60	*G’* 0.1 GPa to 10 KPa<br>*G”* 0.1 GPa to 10 KPa	*ACS Mater. Lett.*<br>2021, 3, 1003
P(T0.7-co-A0.3)	60 to 130	*G’* 0.18 MPa to 4.8 KPa<br>*G”* 0.12 MPa to 4.8 KPa	*Adv. Funct. Mater.*<br>2022, 2112741
SMP0.9GNs0.1	20 to 120	*G’*0.6 GPa to 2 MPa<br>*G”*0.2 GPa to 1 MPa	*Chem. Eng. J.* 2022,<br>433, 133840
Tri-HT	10 to 110	*G’*0.2 GPa to 3 Pa<br>*G”* 0.1 GPa to 150 Pa	This work

Supplementary Table 3. Cohesive energy density of adhesive molecules at different temperatures.

Molecules	Cohesive energy density (GJ m^{-3})		
	-100 °C	25 °C	100 °C
Tri-OH	0.91	0.83	0.79
Tri-Im	0.76	0.69	0.66
Tri-HT	1.41	1.41	1.31
Tri-AT	1.33	1.34	1.24

Meanwhile, the significant change of the CED at variable temperature may account for the observed temperature-sensitive viscoelasticity for Tri-HT.

Supplementary Table 4. Average H-bond number of adhesive molecules at different temperatures.

Molecules	Average H-bond number		
	-100 °C	25 °C	100 °C
Tri-OH	11.5	11.0	10.5
Tri-Im	4.7	4.3	4.2
Tri-HT	10.2	10.1	9.6
Tri-AT	3.6	3.3	3.2

Supplementary Table 5. Results of the multiplication of the signs of each cross-peak in 2DCOS synchronous and asynchronous spectra of Tri-HT.

1629	Simultaneous	-	-	+	+	
1675	Simultaneous	-	-	+		
1702	-	-	-			
3153	+	+				
3320	+					
3567						
	3567	3320	3153	1702	1675	1629

According to Noda's rule, the final specific order for Tri-HT during cooling is given as follows: 1702 → (3567, 1675) → (3567, 1629) → 3320 → 3153 cm^{-1} (→ means earlier than), i.e. Free C=O → Weak H-bonded OH···O=C → Strong H-bonded OH···O=C → H-bonded N-H → Im-C-H.

Supplementary Table 6. Interfacial adhesion energy between adhesive molecules and polyhydric substrate surface.

Molecules	Interfacial adhesion energy (kcal mol^{-1})			
	E_{total}	$E_{molecules}$	$E_{substrate}$	$E_{interfacial}$
Tri-OH	-122617.35	-36175.53	-85334.56	-1107.26
Tri-Im	-126681.74	-40351.00	-85245.98	-1084.76
Tri-HT	-118654.54	-28522.35	-88928.56	-1203.63
Tri-AT	-73721.62	16520.36	-89180.94	-1061.04

Supplementary references

1. Yao, X.*, et al.* Hydrogel Paint. *Adv. Mater.* **31**, e1903062 (2019).
2. Wenzel, F., Agirre, A., Aguirre, M., Leiza, J. R. Incorporation of novel degradable oligoester crosslinkers into waterborne pressure sensitive adhesives: towards removable adhesives. *Green Chem.* **22**, 3272-3282 (2020).
3. Chopin, J.*, et al.* Nonlinear Viscoelastic Modeling of Adhesive Failure for Polyacrylate Pressure-Sensitive Adhesives. *Macromolecules* **51**, 8605-8610 (2018).
4. Shi, L.*, et al.* Highly stretchable and transparent ionic conducting elastomers. *Nat. Commun.* **9**, 2630 (2018).
5. Shang, Y. H., Wu, C., Hang, C. Z., Lu, H. L., Wang, Q. G. Hofmeister-Effect-Guided Ionohydrogel Design as Printable Bioelectronic Devices. *Adv. Mater.* **32**, 2000189 (2020).
6. Ju, Y. H.*, et al.* Pressure-Sensitive Adhesive with Controllable Adhesion for Fabrication of Ultrathin Soft Devices. *ACS Appl. Mater. Interfaces* **12**, 40794-40801 (2020).
7. Beharaj, A., McCaslin, E. Z., Blessing, W. A., Grinstaff, M. W. Sustainable polycarbonate adhesives for dry and aqueous conditions with thermoresponsive properties. *Nat. Commun.* **10**, 5478 (2019).
