# Supplementary material for: Small-molecule ionic liquid-based adhesive with strong room-temperature adhesion promoted by electrostatic interaction
Source: Nat Commun. 2022 Sep 5;13:5214. doi: 10.1038/s41467-022-32997-4 (PMC9445047; doi:10.1038/s41467-022-32997-4)
Supplement: Supplementary file 2 — Description of Additional Supplementary Files [file 41467_2022_32997_MOESM2_ESM.pdf]

Description of Additional Supplementary Files

File Name: Supplementary Movie 1
Description: Flammability test.

File Name: Supplementary Movie 2
Description: Broken bottles repaired using Tri-HT.

File Name: Supplementary Movie 3
Description: Broken bottles repaired using Tri-HT underwater.

File Name: Supplementary Movie 4
Description: Macroscopic adhesion test of TriHT on ceramic substrate.

File Name: Supplementary Movie 5
Description: Macroscopic adhesion test using Joule-heating effect on glass substrate.
